# Integrative Analyses of Circulating Proteins and Metabolites Reveal Sex Differences in the Associations with Cardiac Function among DCM Patients

**DOI:** 10.3390/ijms25136827

**Published:** 2024-06-21

**Authors:** Anke Hannemann, Sabine Ameling, Kristin Lehnert, Marcus Dörr, Stephan B. Felix, Matthias Nauck, Muna N. Al-Noubi, Frank Schmidt, Jan Haas, Benjamin Meder, Uwe Völker, Nele Friedrich, Elke Hammer

**Affiliations:** 1 Institute of Clinical Chemistry and Laboratory Medicine, University Medicine Greifswald, Ferdinand-Sauerbruch-Strasse, D-17475 Greifswald, Germany; matthias.nauck@uni-greifswald.de (M.N.); nele.friedrich@uni-greifswald.de (N.F.); 2 German Centre for Cardiovascular Research (DZHK), Partner Site Greifswald, D-17475 Greifswald, Germany; amelings@uni-greifswald.de (S.A.); kristin.lehnert@uni-greifswald.de (K.L.); marcus.doerr@uni-greifswald.de (M.D.); felix@uni-greifswald.de (S.B.F.); voelker@uni-greifswald.de (U.V.); hammer@uni-greifswald.de (E.H.); 3 Interfaculty Institute for Genetics and Functional Genomics, University Medicine Greifswald, Felix-Hausdorff-Strasse 8, D-17475 Greifswald, Germany; 4 Department of Internal Medicine B, University Medicine Greifswald, D-17475 Greifswald, Germany; 5 Proteomics Core, Weill Cornell Medicine-Qatar, Doha 24144, Qatar; mna2002@qatar-med.cornell.edu (M.N.A.-N.); frs4001@qatar-med.cornell.edu (F.S.); 6 Institute for Cardiomyopathies Heidelberg (ICH), Heart Centre Heidelberg, University of Heidelberg, D-69121 Heidelberg, Germany; jan.haas@med.uni-heidelberg.de (J.H.); benjamin.meder@med.uni-heidelberg.de (B.M.); 7 German Centre for Cardiovascular Research (DZHK), Partner Site Heidelberg/Mannheim, D-69121 Heidelberg, Germany; 8 Department of Medicine III, University of Heidelberg, INF 410, D-69120 Heidelberg, Germany

**Keywords:** DCM, proteomics, metabolomics, phenotype association, correlation, OLINK, LC MS/MS, Biocrates

## Abstract

Dilated cardiomyopathy (DCM) is characterized by reduced left ventricular ejection fraction (LVEF) and left or biventricular dilatation. We evaluated sex-specific associations of circulating proteins and metabolites with structural and functional heart parameters in DCM. Plasma samples (297 men, 71 women) were analyzed for proteins using Olink assays (targeted analysis) or LC-MS/MS (untargeted analysis), and for metabolites using LC MS/MS (Biocrates AbsoluteIDQ p180 Kit). Associations of proteins (n = 571) or metabolites (n = 163) with LVEF, measured left ventricular end diastolic diameter (LVEDD^measured^), and the dilation percentage of LVEDD from the norm (LVEDD^acc. to HENRY^) were examined in combined and sex-specific regression models. To disclose protein–metabolite relations, correlation analyses were performed. Associations between proteins, metabolites and LVEF were restricted to men, while associations with LVEDD were absent in both sexes. Significant metabolites were validated in a second independent DCM cohort (93 men). Integrative analyses demonstrated close relations between altered proteins and metabolites involved in lipid metabolism, inflammation, and endothelial dysfunction with declining LVEF, with kynurenine as the most prominent finding. In DCM, the loss of cardiac function was reflected by circulating proteins and metabolites with sex-specific differences. Our integrative approach demonstrated that concurrently assessing specific proteins and metabolites might help us to gain insights into the alterations associated with DCM.

## 1. Introduction

Dilated cardiomyopathy (DCM) is currently defined as a heart muscle disease with left ventricular dilatation and systolic dysfunction in the absence of pressure or volume overload or coronary artery disease that is sufficient to explain the cardiac dysfunction [1]. DCM highly contributes to the clinical syndrome of heart failure with reduced ejection fraction (HFrEF). It is a severe disease with substantial mortality [2].

Causes of DCM are manifold and include non-genetic causes such as viral infections and myocarditis, endocrine disorders, immune dysregulation and autoimmune diseases, toxic and metabolic conditions, as well as genetic causes [3]. A genetic determinant can be detected in up to 40% of DCM patients, and many genes involved in the pathogenesis of DCM have been identified. These include genes coding for sarcomere proteins, ion channels, and/or intercellular junction molecules, for example, titin and phospholamban mutations [4]. Identifying the underlying cause of DCM may thus help to select specific drug therapies or necessitate the screening and genetic testing of relatives [3]. Moreover, a thorough clinical workup of DCM patients is required to determine reversible causes [5]. The significant heterogeneity among DCM patients calls for intensified research on disease mechanisms and prognostic factors. In this context, the use of OMICs techniques, including proteomics and metabolomics, appears promising [6,7]. Proteins and metabolites are closely related to the phenotype [6,7], and alterations in the individual protein or metabolite profile have been suggested as indicators of pathophysiological changes associated with DCM [6,8]. Metabolites from the classes of acylcarnitines (ACs), branched-chain amino acids, and carboxylic acids, among others, have been shown to be related to mechanistic changes in DCM and to play a role as prognostic markers in DCM (for a review, see Ampong et al. [6]), but they are not yet used in clinical practice. The value of proteomics in the field was demonstrated in a recent study that developed and validated a protein risk score including eight known and novel proteins that predict death or worsening of heart failure in HFrEF patients [9].

One commonly used strategy to identify proteins or metabolites involved in the pathophysiology of DCM is the case–control approach. It allows for the identification of markers that differentiate between patients with DCM, those with other causes of heart failure, and healthy controls [10,11,12,13]. Altered protein or metabolite levels were mainly related to mitochondrial (dys-)function, lipid metabolism, or inflammation [6,9,10]. Another approach, which has not been applied in former studies, is to assess inter-individual differences across the disease spectrum. Such analyses may be valuable as they allow for the determination of continuous alterations from early- to late-stage disease. Thus far, integrative analyses using proteomics and metabolomics data from a single patient cohort are missing. A combined interpretation of results from both OMICs layers may yield more detailed insights into the complex regulations of DCM. Finally, previous studies have failed to evaluate potential sex differences, despite the notable discrepancy in the proportion of men (about 75%) and women impacted by DCM [5]. The purpose of this study is, therefore, to evaluate sex-specific relationships between proteins and metabolites with structural and functional heart parameters among a carefully selected cohort of DCM patients.

## 2. Results

### 2.1. Characteristics of the DCM Patients in the Discovery Cohort

Table 1 presents the clinical characteristics of the 368 DCM patients (297 men and 71 women) from the discovery cohort. While the age range of the patients was large (16–82 years), half of all patients were middle-aged (1st–3rd quartile of age: 47.9–63.7 years), about 10% were younger than 40 years, and another 10% were aged 70 years or older. The average DCM patient was overweight, with a median BMI of 28.0 kg/m^2^, and about 31% were smokers. Median LVEF values were 30.0% (men) and 33.0% (women), median LVEDD^measured^ values were 69 mm (men) and 63 mm (women), and median LVEDD^acc. to HENRY^ values were 140% (men) and 135% (women), respectively. About 89% of DCM patients had a LVEF ≤ 40%, and 77% of DCM patients had HFrEF, defined as LVEF ≤ 40% and NYHA II-IV (Table 1).

### 2.2. Lack of Association between LVEDD and Proteins or Metabolites

The results of the analyses for LVEDD^measured^ and LVEDD^acc. to HENRY^ were comparable. We, therefore, will only report the LVEDD^acc. to HENRY^. A total of 266 targeted proteins, 307 untargeted proteins, 166 metabolites, and 3 metabolite ratios were assessed. In combined models including men and women, only 3 of the 573 proteins and none of the metabolites or metabolite ratios demonstrated significant associations with LVEDD^acc. to HENRY^. In sex-specific analyses, it became evident that the three with LVEDD^acc. to HENRY^-associated proteins were only significant in men. Further associations in men were not observed. In women, associations were completely absent. Respective results are given in Supplemental Figures S2–S4 and Supplemental Tables S3, S5 and S7.

### 2.3. Associations between LVEF and Proteins or Metabolites

A total of 95 proteins (LC-MS/MS n = 42; OLINK n = 43) and 32 metabolites or metabolite ratios were significantly related with LVEF after adjustment for age, sex, and BMI in the combined models. In sex-specific analyses, adjusted for age and BMI, 115 proteins and 35 metabolites or metabolite ratios were associated with LVEF in men, but no proteins and only 1 metabolite were associated with LVEF in women (Supplemental Figures S2–S4, Supplemental Tables S2–S7). Hence, the male patients in the discovery cohort determined the observed associations with LVEF in the whole sample. The lower number of female DCM patients in our discovery cohort (about 1/3 of men) might have contributed to a lower power of the analysis in women and, therefore, to the lack of observed effects. To distinguish the power driven from true sex-specific differences, the effect sizes observed in men and women were compared (Supplemental Figure S5). The comparisons showed similar effects in men and women for a low number of proteins and metabolites (especially for lysoPCs). These results imply true sex-related differences for most of the examined proteins and metabolites. Thus, the focus of the following analyses was put on male DCM patients.

### 2.4. Associations between LVEF and Proteins in Men

A combination of non-targeted analyses by LC-MS/MS and targeted analysis by PEA was employed to investigate a wide concentration range of circulating plasma proteins (Supplemental Figures S2 and S3, Supplemental Tables S2 and S4). Hence, 115 significant associations with LVEF in men represented 58 untargeted proteins with higher concentrations in blood plasma, as well as 57 targeted proteins quantified by PEA with higher sensitivity.

**Figure 1:**
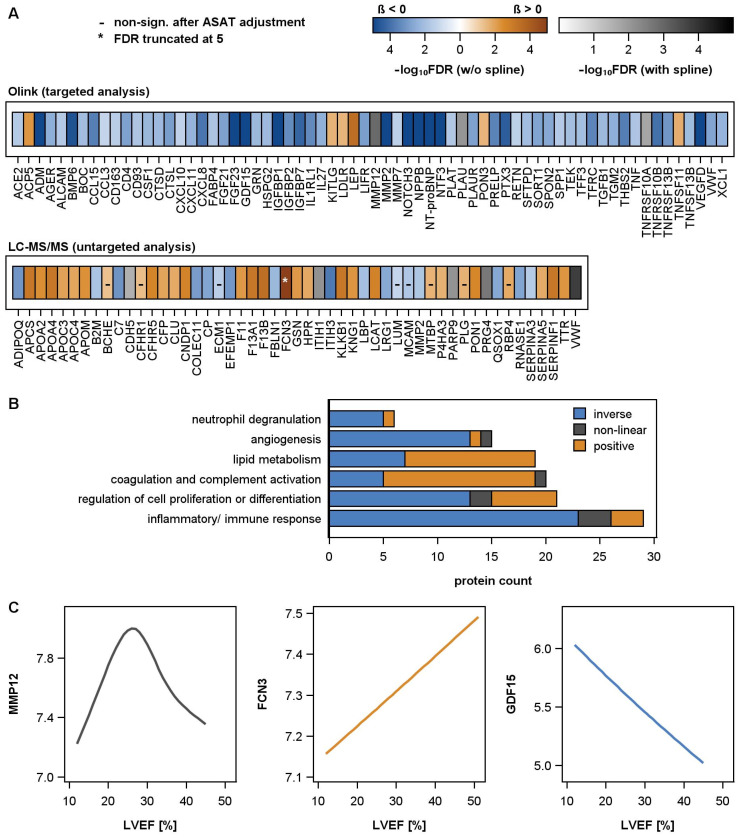
(**A**) Heatmap illustrating the significant associations of left ventricular ejection fraction (LVEF) and the plasma proteins in male DCM patients. Results from regression models were adjusted for age and BMI. The color gradient illustrates the false discovery rate (FDR) obtained after correction for multiple testing. A blue-to-orange color gradient highlights a linear association, with blue color indicating an inverse and orange color a positive association. A white-to-black color gradient highlights a non-linear association using restricted cubic splines. A black line within a box implies that the association is no longer significant after additional adjustment for aspartate amino transferase (ASAT) activity. An asterisk within a box implies that the FDR was truncated at a value of 5. In the targeted analyses, ten proteins were present on the inflammation and CVD II or III panel. To reduce redundancies, the results from the inflammation panel are not illustrated. (**B**) Categorization of 115 unique proteins by Gene Ontology terms. (**C**) Illustration of an exemplary non-linear, positive, and inverse association between selected proteins (MMP12, FCN3, GDF15) and LVEF.

Overall, more proteins had inverse and fewer had positive associations with LVEF (Figure 1A,B). Non-linear alterations between circulating proteins and LVEF were rare (5.2% of the significant associations, (Figure 1C).

Proteins associated with LVEF were assigned to Gene Ontology terms, and they belonged to six major categories (Figure 1B). The majority of proteins involved in lipid metabolism showed decreasing amounts with reduced LVEF, similarly to proteins involved in coagulation and complement activation. In contrast, higher portions of proteins assigned to all the other categories increased with the loss of systolic heart function. The category of inflammatory proteins and immune response included, among others, angiotensin-converting enzyme 2 (ACE2) and chemokines (CCL15, CXCL11), but also tumor necrosis factor (TNF), TNF-related receptors (10A and B), and TNF-related ligands (TRANCE (TNFSF11), BAFF (TNFSF13B)). The category of angiogenesis presented the well-known cardiac stress markers BNP (NPPB) and NT-proBNP, as well as further factors involved in regulation of angiogenesis, like vascular endothelial growth factor (VEGFD), pigment epithelium-derived factor (SERPINF1), angiopoetin-1 receptor (ANGPT1), cadherin-5 (CDH5), and IL8 (CXCL8), but also proteins promoting cellular responses to stimuli (CXCL10 or MMP2 and thrombospondin-2(THBS2)).

### 2.5. Associations between LVEF and Metabolites in Men

**Figure 2:**
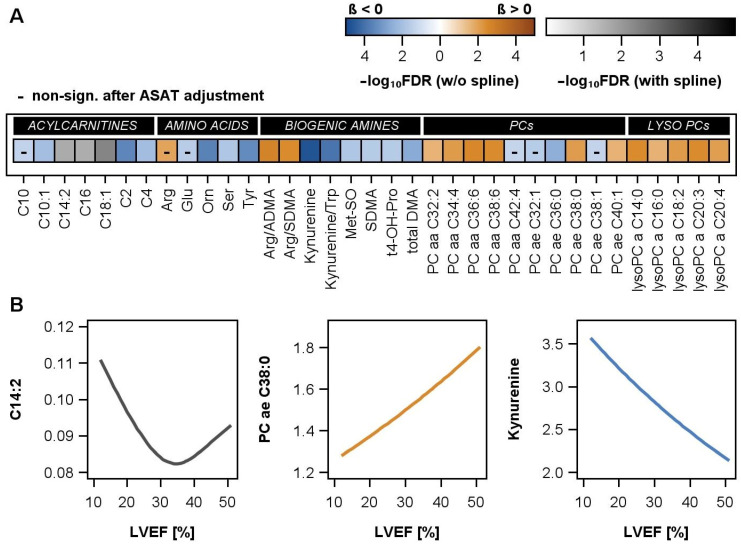
(**A**) Heatmap illustrating the significant associations of left ventricular ejection fraction (LVEF) and the plasma metabolites with their respective substance classes in male DCM patients. Results from regression models were adjusted for age and BMI. The color gradient illustrates the false discovery rate (FDR) obtained after correction for multiple testing. A blue-to-orange color gradient highlights a linear association, with blue color indicating an inverse and orange color a positive association. A white-to-black color gradient highlights a non-linear association using restricted cubic splines. A black line within a box implies that the association is no longer significant after additional adjustment for aspartate amino transferase (ASAT) activity. (**B**) Illustration of an exemplary non-linear, positive, and inverse association between selected metabolites (C14:2, PC ae C38:0, kynurenine) and LVEF. PC = phosphatidylcholine.

In the large group of phosphatidylcholines (PCs) and lysophosphatidylcholines (lysoPCs), eleven metabolites were positively associated and four metabolites were inversely associated with LVEF in men (Figure 2A, Supplemental Figure S4, Supplemental Table S6). After an additional adjustment for ASAT activity, all positive associations, but only one of the inverse associations, remained statistically significant. PCs and lysoPCs, thus, mainly decreased with the progression of DCM. Analogue associations were found for the arginine/ADMA and arginine/SDMA ratio. Further metabolites in the classes of amino acids and biogenic amines were inversely associated with LVEF, e.g., kynurenine and the kynurenine/tryptophan ratio. Finally, four inverse and three non-linear associations between ACs and LVEF were present (Figure 2B), but none of the examined sphingomyelins or hexose demonstrated an association with LVEF (Supplemental Table S6).

### 2.6. Associations between LVEF and Metabolites in the Validation Cohort

In male DCM patients from the validation cohort, the effect estimates of the examined relations pointed largely in the same direction as in the discovery cohort, i.e., they were positive for most PCs and lysoPCs and inverse for several ACs, amino acids, and biogenic amines (Supplemental Figure S6). Also, the effect sizes in the discovery and replication cohort were, for the majority of metabolites, comparable (Supplemental Figure S7). A very good agreement was observed for kynurenine, the kynurenine/tryptophan ratio, SDMA, the arginine/SDMA ratio, and several PCs and lysoPCs. Above this, in the validation cohort, associations between increasing kynurenine or the kynurenine/tryptophan ratio with decreasing LVEF were nominally significant (*p* < 0.05), but failed the correction for multiple testing (FDR > 0.05). The lower number of patients in the validation cohort and the related smaller statistical power probably caused this observation of very similar effects, although statistical significance was missing.

### 2.7. Correlation between Proteins and Metabolites

**Figure 3:**
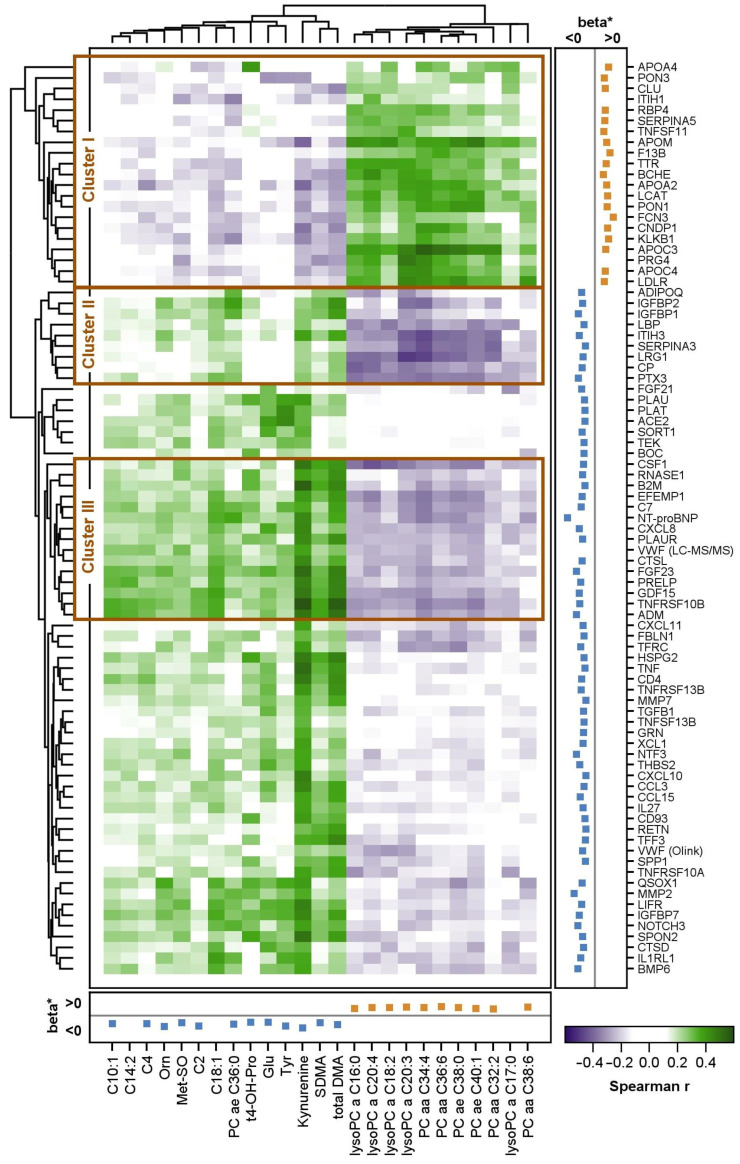
Heatmap illustrating the correlation between proteins and metabolites that were significantly associated with LVEF. Spearman correlation coefficients are illustrated for those proteins and metabolites that had at least one correlation coefficient above |0.3|. The clustering was based on the average linkage method. Positive correlation coefficients are illustrated in green; negative correlation coefficients in purple. * In addition to the correlation coefficients, the effect estimates (ß) from the associations between the proteins and metabolites with LVEF are presented next to the protein and metabolite names. Positive associations are colored in orange; inverse associations in blue. Note that effects for non-linear associations are not displayed. In the targeted protein analyses, ten proteins were present on the inflammation and CVD II or III panel. To reduce redundancies, the results from the inflammation panel are not illustrated.

In the discovery cohort, 85 proteins and 25 metabolites were associated with LVEF, had a Spearman correlation coefficient of |0.3| for at least one metabolite, and entered the cluster analysis (Figure 3). Here, kynurenine exhibited the highest number of correlations and the highest correlation coefficients (r −0.40–0.58) with the proteins. Most of the correlations were positive and present with proteins with higher levels at low LVEF. Strong correlations were found with members of the tumor necrosis factor (TNF)/TNF receptor superfamily (TNF, TNFSF13B, TNFRSF10A, TNFRSF10B, TNFRSF13B) and beta-2-microglobulin, a component of the MHC-I complex. In contrast, a group of lysoPCs showed predominantly inverse correlations with these proteins, which was due to their low levels at low LVEF. Surprisingly, a strong, positive correlation was observed between APOA4, which is involved in, e.g., reverse cholesterol efflux, and trans-4-hydroxyproline (t4-OH-Pro), whose increase in blood or urine indicates increased collagen degradation.

The overall results revealed three interesting clusters with high correlation coefficients between proteins and metabolites. The first cluster (cluster 1) included several proteins related to lipid metabolism. These were positively correlated with PCs and lysoPCs and inversely with kynurenine, SDMA, and total DMA. Cluster 2 included nine proteins with opposite correlations to the same metabolites as cluster 1. These proteins included insulin growth factor-binding proteins 1 and 2 (IGFBP1 and IGFBP2), proteins of the innate immune system, as well as proteins involved in acute phase response (PTX3, LBP, CP, SERPINA3, LRG1). Cluster 3 included 15 proteins. It included proteins like NT-proBNP (NPPB), growth differentiation factor 15 (GDF15), and fibroblast growth factor 23 (FGF23), markers that are known to be tightly related to heart failure and further systemic diseases, but also inflammatory proteins (CXCL8, CSF1).

A functional enrichment analysis of proteins in clusters 1, 2, or 3 revealed a significant result only for cluster 1. The protein set and the decrease in circulating levels at low LVEF point to a significant reduction in *plasma lipoprotein assembly, remodeling, and clearance* with the worsening of DCM. Analysis of the total protein set correlating with metabolite levels in plasma supported the hypothesis of an increased *inflammatory response and immune cell trafficking* at low LVEF.

## 3. Discussion

In contrast to published works that have described case–control studies and have usually focused on one omics level only, this work examined the relationships between functional and structural cardiac parameters as assessed by LVEF, LVEDD^measured^, and LVEDD^acc. to HENRY^ with circulating proteins and metabolites, and stratified them by sex.

Using mass spectrometry-based protein and metabolite profiling techniques, as well as the sensitive proximity extension assay Olink, we demonstrated that circulating proteins and metabolites are associated with LVEF, but not with LVEDD^measured^ nor with LVEDD^acc. to HENRY^. Besides linear modeling, the inclusion of restricted cubic splines disclosed a few non-linear associations of LVEF with plasma proteins and metabolites. The effects of LVEF on the metabolites in men were replicated in an independent, but smaller, validation cohort. Further, the observed significant associations of proteins and metabolites with LVEF point to a continuous adaptation of lipid metabolism, protective molecules, and an increase in severity indicators with a loss of systolic function.

The present study included a high number of precisely characterized DCM cases. As expected, a substantial number of associations between LVEF and circulating molecules was identified. Although there was some correlation between LVEF and LVEDD^acc. to HENRY^ (Supplemental Figure S8), surprisingly few associations were found with relative LV diameter. Yet, the observation of associations of LVEDD^acc. to HENRY^ with BNP and NT-proBNP, the most important biomarkers for heart failure, and vascular endothelial growth factor D (VEGFD), a novel biomarker for left pulmonary hypertension subsequent to left heart disease [15], can be regarded as general proof of the principles of the present analyses. The absence of further associations with LVEDD^acc. to HENRY^ might indicate that the proteins and metabolites investigated here are more closely associated with contractile function than with the structural properties of the heart muscle.

In DCM, the awareness of sex-specific aspects of disease-related changes is becoming increasingly important [16]. Men are not only more often affected by DCM than women, but they also present with lower LVEF, worse outcomes, and higher mortality [2,17]. The suspected reasons for these discrepancies include lifestyle and socioeconomic factors [18], but also the differential effects of estrogen and testosterone [19] and differences in immune response preceding DCM development [17]. Therefore, separate analyses were performed for male and female patients. In accordance with the higher frequency of DCM in men [5], only about 20% of the patients included in the discovery cohort were female. In the analysis of the female data set, which consisted of 71 patients, no significant associations, except for one metabolite, were detected. The low number of women led to larger confidence intervals and higher *p*-values than in men, while effect sizes were not compromised. The comparison of the effect sizes in men and women revealed only a few proteins and metabolites (especially lysoPCs) with similar estimates, while the majority of the results differed. This suggests that true sex-specific differences exist. However, these observations should be focus of future research taking meta-analyses of women from different cohorts into account.

Alterations of plasma proteins in heart failure have been studied extensively to identify disease-specific or prognostic signatures (reviewed by Michelhaugh et al. [20], Adamo et al. [21], and Liu et al. [22]). However, there are few studies that refer to well-defined cohorts of patients with cardiomyopathies [23,24,25], and the results of these studies depend on the methods used. Thus, MS-based studies tend to reflect changes in classical plasma proteins [23,24], whereas proximity extension assays are more likely to measure tissue leakage proteins [25] (Supplemental Figure S9). To overcome this discrepancy, both methods were applied in the current study.

The metabolic profiling was performed with a validated and commonly used kit. In the discovery cohort, associations between reductions in LVEF and several ACs, PCs, lysoPCs, kynurenine, the kynurenine/tryptophan ratio, as well as arginine, ADMA, SDMA, and its ratios were observed. As tendencies towards similar associations, or at least effect estimates with the same direction, were observed in the replication cohort, these metabolites seem to be particularly relevant to DCM.

### 3.1. Integration of Proteomic and Metabolomic Results

The proteins and metabolites associated with declining LVEF are consistent with previous reports and highlight diverse pathologic changes related to DCM. The novelty of our approach is the integrative analysis of different OMICS levels, which allows for a deeper characterization of the disease.

DCM patients experience alterations in cardiac energy metabolism, with a long-term shift away from the highly efficient fatty acid oxidation to increased glucose utilization [26]. In line with this, our analyses revealed a clustering of several proteins and metabolites related to lipid metabolism (cluster 1). Among the 21 proteins in cluster 1, at least 17 were HDL-associated [27]. These included several well-known HDL-associated apolipoproteins, such as APOA2, APOA4, APOC3, APOC4, APOJ, APOM, PON1, and PON3 [28]. Similarly to LCAT, an enzyme required for HDL particle growth and maturation [29], and the LDL receptor (LDLR), which mediates uptake of LDL in cells [28], the proteins demonstrated positive correlations with several PCs and lysoPCs. Inflammatory processes, fibrosis, and oxidative stress are well-known features of DCM [30]. The HDL-associated proteins PON1 and PON3 serve as important players protecting against oxidative stress [31]. The described decrease in PON1 and PON3 with worsening DCM is in line with this. Further, PON1 has previously been reported to be lower in non-ischemic DCM than in patients with HFpEF and healthy controls [23]. While data on the action of HDL-associated proteins in DCM are sparse, the aforementioned mechanisms might have contributed to our observations.

Changes in lipid metabolism are also linked to changes in phosphatidylcholine (PC) and lysophosphatidylcholine (lysoPCs) levels in DCM. A reduction in PC biosynthesis impairs the secretion and metabolism of triglyceride-rich lipoproteins, affects lipid droplet formation, and may provoke increased de novo lipogenesis (for a review see [32]). It is, thus, not surprising that low PC levels have been found in diseases related to disturbed energy metabolism, including heart failure [33,34,35]. In line with this, we observed generally lower PC levels with a declining LVEF.

A link between disturbances in lipid metabolism and inflammatory processes in DCM was suggested by the observation of inverse correlations between kynurenine and the proteins in cluster 1. Kynurenine is synthesized from tryptophan and related to inflammatory processes, which may represent a link to heart failure [36]. Our results, obtained in DCM patients presenting with 90% HFrEF, are consistent with previous findings of increasing kynurenine with decreasing LVEF, increasing severity of chronic heart failure (NYHA I-IV) [37], and a corresponding positive association with NT-proBNP [37]. Very recently, Wang and colleagues [38] found elevated kynurenine and indole amine 2,3-dioxygenase 1 (IDO1) levels in DCM patients. Even more interestingly, the depletion of IDO1 or inhibition of kynurenine in murine models was found to prevent the progression of cardiac hypertrophy and may represent a potential treatment target [38].

Cluster 2 presents proteins with strong inverse associations to lysoPCs and long-chain PCs and moderate positive correlations with kynurenine and total DMA. The complex effects of lysoPCs on human metabolism are incompletely understood. They are thought to exert several deleterious effects on the cardiovascular system [39], but are also discussed as anti-inflammatory molecules, and low levels might promote chronic inflammatory states. Recent clinical studies have demonstrated decreased lysoPC concentrations in heart failure [34,35,40], and our own data extend this to DCM patients. In line with this, inflammatory proteins (PTX3, LBP, CP) showed strong inverse correlations to selected lysoPCs. The usefulness of anti-inflammatory therapy in the treatment of cardiomyopathy is under debate [41], but positive effects could not be verified either for chronic inflammation, as observed by the circulating molecular pattern in our DCM patients, or for acute inflammatory disease states [42]. Conventional heart failure therapies, on the other side, might also contribute to immune modulation via the suppression of macrophage-derived cytokines or the reduction in lymphocyte activation markers (summarized by [43]). Our DCM patients partially displayed increased leukocyte infiltration (Table 1), but a general acute inflammatory signature was not observed on the OLINK panel. Nevertheless, several proteins of cluster 3 are related to inflammatory processes and macrophage origins like IL-8 (CXCL8) or CSF1, underlining the suspected chronic inflammatory status of DCM patients [43].

Cluster 3 includes proteins with strong positive correlations with kynurenine, SDMA, total DMA, and several ACs, as well as inverse correlations with PCs and lysoPCs. Among the proteins, NT-proBNP, GDF15, FGF23, B2M [44], TNFRSF10B [45], and uPAR (PLAUR) [46] represent known biomarkers for the prognosis of cardiovascular outcome and mortality risk prediction. The continuous increase in GDF15, FGF23, and soluble interleukin 1 receptor-like 1 (IL1RL1) with decreasing LVEF reflect the insufficient oxygen and nutrient supply of the body and the resulting injury to multiple organs. The strong correlation between those risk factors and the medium- and long-chain ACs C10:1, C14:2, and C18:1, especially when LVEF falls below 30%, once more highlights the interconnection between cardiac mitochondrial dysfunction and heart failure in DCM. Accumulation of circulating ACs [47,48] results from the switch from fatty acid oxidation to increased glucose utilization [26] under cardiac stress. The inverse associations between LVEF and several short- (C2, C4) and medium-chain (C10, C10:1) ACs identified here support these notions. A novel finding of our analysis was the observation of non-linear associations between LVEF and three long-chain ACs in the discovery cohort. These potential non-linear associations raise the question of whether circulating ACs start accumulating at different stages of disease severity. Yet, the lack of full validation and of other studies assessing non-linear associations prevents us from drawing firm conclusions from the present analyses.

The inverse associations of LVEF with SDMA and total dimethylarginine (DMA) point to the important role of endothelial dysfunction in DCM [49]. ADMA and SDMA are methylated forms of arginine that interfere with nitric oxide production, a potent vasodilator [50,51]. Arginine has been reported to be dramatically decreased in DCM patients vs. controls and positively correlated with LVEF [11]. Also, in the present analyses, arginine was positively associated with LVEF and positive correlations of total DMA with several proteins related to endothelial/vascular function. Thus, RNASE1 is crucial for the degradation of extracellular RNA (eRNA), which increases upon inflammation and injury [52]. Although eRNA levels in DCM have not been reported so far, the observed higher RNASE1 levels in DCM patients might be considered protective, since RNASE1 supplementation [53] or induction [54] exerts positive effects on the cardiovascular system by reducing cytokine release and antioxidant enzyme levels. Another regulator of the vascular system and endothelial integrity is the peptide hormone adrenomedullin (ADM). From recent results, it has been concluded that ADM acts as a functional antagonist to angiotensin II, thereby inhibiting aldosterone secretion and thus compensating for renin–angiotensin–aldosterone system escalation. Since high ADM plasma levels are protective, its degrading enzyme, the neutral endopeptidase neprilysin, has been recognized as new target for heart failure patients [55]. The positive correlation between these proteins and arginine, DMA, and SDMA, which are all involved in vasodilation, highlights endothelial dysfunction as an essential part of DCM and as a possible therapeutic target.

Further prominent members of cluster 3 are involved in collagen binding and deposition, like prolargin (PRELP) and cathepsin L (CTSL). Thus, the increases in these molecules with the loss of LVEF point to a systemic alteration of extracellular matrix remodeling.

The role of viral infections in autoimmune DCM has been a research focus for decades (for a review, see [56]). Primed T-cells that first destroy virus-infected cardiomyocytes may trigger secondary effects by later targeting auto-antigens with similarity to viral epitopes. We identified correlations between metabolites with proteins involved in TNF-alpha and cytokine pathways as well as in T-cell and B-cell regulation, which might act as immunological mediators. TNF-alpha itself, but also receptors and ligands of the TNF superfamily, point to a disturbed immune response in DCM patients despite the absence of bacterial or viral antigens. High levels of B-cell activating factor BAFF (TNFSF13B) and TNF receptor superfamily member 13B (TNFRSF13B), both involved in B-cell homeostasis and regulation, underline the hypothesized role of B-cell regulation in DCM [57], but have to be studied at the immune cell level in more detail.

### 3.2. Strengths and Limitations

The present study stands out due to its integrative design combining protein and metabolite measurements. A large number of proteins from a targeted and an untargeted approach, as well as metabolites from an established LC-MS/MS method, were evaluated with respect to their effects on cardiac function in DCM. The look-up of our results in a second DCM cohort using similar metabolite measurements is another strength of the present study. Indeed, the effects of LVEF demonstrated similar direction and strength in the discovery and validation cohorts. For these metabolites, the lack of statistical significance might be attributed to the smaller number of DCM patients in the validation cohort. In addition, it must be acknowledged that the analyses of women in the discovery cohort may have been underpowered, which is a limitation of the present study. Further, the cross-sectional data investigated herein prohibit an assessment of whether these observed effects are causes or consequences of DCM. Finally, our results show high similarity to previous data obtained from case–control or association studies on heart failure patients with different etiologies. Therefore, conclusions cannot be considered to be DCM-specific. Nevertheless, the present results expand our knowledge on molecular alterations related to loss of LV function.

### 3.3. Conclusions

In DCM, the loss of cardiac function is accompanied by alterations of circulating proteins and metabolites. The presented results disclose sex-specific differences in disease-related potential biomarkers and emphasize the need for comprehensive studies focusing on men and women separately to close the gaps in our understanding of DCM progression, as illustrated here. Our integrative approach highlights changes in lipid metabolism, characterized by mitochondrial dysfunction and impaired lipoprotein functionality, as well as inflammatory processes and endothelial dysfunction as major drivers associated with cardiac dysfunction. The LVEF-associated molecules are partially known from heart failure studies of different etiologies and are not fully DCM-specific. Nevertheless, it may prove useful to assess proteins and metabolites related to disturbed lipid metabolism, metabolic homeostasis and inflammatory status simultaneously in order to elucidate DCM progression and support the identification of potential therapeutic targets for the individual treatment of patients.

## 4. Materials and Methods

### 4.1. Discovery Cohort

The present study includes adult DCM patients enrolled in a registry cohort between 2005 and 2014 in Northeast Germany. All enrolled patients had suspected DCM. Of those, all individuals with acute infectious diseases, cancer treated with chemotherapy using cardiotoxic components, chronic alcoholism, postpartum cardiomyopathy, coronary heart disease, or heart failure due to other known origins (e.g., primary valvular disease) were excluded. All patients displayed global, but not segmental, contractility impairment. In accordance with the Dallas criteria, histopathological analysis of endomyocardial biopsies was used to define acute myocarditis, and proven cases were excluded. In total, 368 subjects presented with non-ischemic DCM and had echocardiographic data. DCM was defined as a left ventricular ejection fraction (LVEF) of <45% [3] or dilatation percentage of the left ventricular end diastolic diameter (LVEDD^acc. to HENRY^ [58]) of ≥117% from the norm [59]. In subsequent analyses, to maximize the sample size for each model, the respective numbers of subjects with missing information in LVEDD^acc. to HENRY^ (n = 2) were excluded from the analyses.

All patients provided venous blood samples. In these samples, aspartate amino transferase (ASAT) activity was measured using either the Dimension RxL (patients enrolled until December 2008) or the Dimension Vista (patients enrolled after December 2008) system (both systems: Siemens Healthcare Diagnostics, Eschborn, Germany).

Written informed consent was obtained from all individual participants included in the study (SFB/TR 19: Reg-Nr IIIUV 30/04–34/04). All procedures performed were in accordance with the ethical standards of the institutional and/or national research committee, as well as with the 1964 Helsinki Declaration and its later amendments or comparable ethical standards.

### 4.2. Validation Cohort

For the validation of the associations between LVEF and the metabolites, data from DCM patients recruited at the University of Heidelberg were obtained [10]. In these patients, serum metabolites were quantified using the same kit (AbsoluteIDQ p180 Kit, Biocrates Life Sciences AG, Innsbruck, Austria) as in the discovery cohort. The validation cohort included 101 male DCM patients, among which 93 patients had full data on LVEF, LVEDD^acc. to HENRY^, age, and BMI and were included in the analyses. All metabolites significantly associated with LVEF in the discovery cohort were selected, and their associations with LVEF were examined in the validation cohort. Further details on the recruitment and characteristics of the Heidelberg DCM patients are presented elsewhere [10].

### 4.3. Echocardiography

Echocardiographic parameters (biplane LVEF, according to Simpson’s rule, and LVEDD) were determined by two-dimensional echocardiography, as described earlier [14]. The dilatation percentage of the norm (LVEDD^acc. to HENRY^) was calculated as the relation of the measured LVEDD (LVEDD^measured^ in mm) with the predicted value obtained from age and body surface area, and the formula of Henry was used [58]. In the subsequent analyses, these relative values were assessed.

### 4.4. Global Protein Profiling

Plasma was obtained by centrifuging EDTA blood (10 min, 3.600 g, room temperature) and was stored at −80 °C until further use. Sample preparation for tandem mass spectrometry for non-hemolytic samples was carried out as described earlier [23]. Briefly, samples were depleted of six highly abundant proteins using a MARS Hu-6-column (Agilent Technologies, Santa Clara, CA, USA) according to the manufacturer’s recommendation. After estimation of the total protein content using a Bradford Protein Assay (Bio-Rad Laboratories, Inc., Hercules, CA, USA), samples were subjected to tryptic digestion (protein to trypsin ratio 25:1). Peptides were desalted on Oasis C18 plates (Waters Corp, Manchester, UK) and analyzed by means of shotgun liquid chromatography–tandem mass spectrometry (LC-MS/MS) using a LTQ-Orbitrap Velos (Thermo Electron, Bremen, Germany) mass spectrometer. Proteins were identified and quantified using the Andromeda algorithm and a human Uniprot database (v 01_2021) implemented in MaxQuant v2.0.1.0. Peptides and proteins were annotated at a false positive rate of 1%. For protein quantification, only razor (Occam’s razor principle) and unique peptides were considered, and normalized protein intensities (Label Free Quantification (LFQ) values) were exported from MaxQuant. The resulting values were log10-transformed for subsequent analysis. In total, 307 proteins were quantified using this method. A detailed description of the methods is provided in the online Supplementary Materials (Supplemental Table S1).

### 4.5. Targeted Protein Analysis by Proximity Extension Assay

Plasma samples were measured using the Target 96 Proximity Extension Assays (PEA) CVD II, CVD III, and Inflammation (Olink Bioscience, Uppsala, Sweden) [60]. Each of the 92 analytes per panel, if present, was detected by two specific antibodies, resulting in the formation of a unique DNA reporter sequence, which was amplified by real-time PCR. Plasma protein levels were indicated by the amplified polymerase chain reaction signals. The resulting relative NPX (normalized protein expression) values were log2-transformed for subsequent analysis and normalized according to the manufacturer’s recommendations. Ten protein assays (CCL2, CCL3, CXCL1, FGF21, FGF23, IL6, IL18, KITLG, TNFRSF11B, PLAU) were present on the inflammation and CVD II or CVD III panels. Therefore, of the 276 targeted proteins, 266 were unique and 10 were measured in duplicate.

### 4.6. Verification of Protein Measurements

To verify the protein measurements, four proteins, galectin 3 (GAL3), ST2, osteopontin (OPN), and MMP2 were additionally measured by enzyme-linked immunosorbent assay (ELISA) using kits from R&D Systems (Frankfurt, Germany). The agreement of the ELISA and OLINK or LC-MS/MS measurement results was assessed using Pearson correlation. Statistically significant, moderate to very high correlations (r between 0.55–0.94) were observed (Supplemental Figure S1), approving the measurement results. Further, two proteins, VWF and MMP2, were determined by untargeted as well as targeted protein profiling. Both proteins, from untargeted as well as targeted measurements, were significantly associated with LVEF, underlining the robustness of the study’s results (Figure 1).

### 4.7. Functional Categorization of Proteins and Nomenclature

We used Gene Ontology terms and the Ingenuity Pathway analysis software suite (v03/2023) for the assignment of proteins to functional categories and pathways. The proteins were named based on their corresponding gene names. There was one exception to that: as NPPB refers to both the brain natriuretic peptide (BNP) and N-terminal proBNP (NT-proBNP), the latter was named NT-proBNP.

### 4.8. Targeted Metabolomic Profiling

In the discovery cohort, targeted metabolomic profiling of plasma samples was performed using the AbsoluteIDQ p180 Kit (Biocrates Life Sciences AG, Innsbruck, Austria), as previously described [61]. In short, using this method, up to 188 metabolites from five different compound classes were quantified. Via flow injection analysis, ACs, phospholipids, and sphingolipids were measured in positive ionization mode, whereas the sum of hexoses was measured in negative ionization mode. Amino acids and biogenic amines were analyzed by LC-MS/MS using the Agilent 1260 Infinity Binary LC (Santa Clara, CA, USA) with an Agilent C18 column connected to an AB SCIEX 5500 QTrap™ mass spectrometer (AB SCIEX, Darmstadt, Germany). Pre-processed data were uploaded into the Biocrates MetIDQ software (April/May 2017), and the metabolite concentrations were automatically calculated.

For quality control purposes, external material in three concentrations (quality control 1 (QC1) = low concentration, QC2 = medium concentration, QC3 = high concentration), was measured together with the study samples on each plate. QC2 was used as the reference to account for plate variation. Measured concentrations of the metabolites were divided by the median concentration of all QC2 concentrations for each metabolite on each plate. Afterwards, the median of the plate medians of the QC was used to reset to the original scale. Metabolites that had a coefficient of variation of more than 25% in QC2 were excluded from all further analyses. This yielded 163 metabolites. Additionally, three metabolite ratios were calculated: kynurenine/tryptophan, arginine/asymmetric dimethyl arginine (ADMA), and arginine/symmetric dimethyl arginine (SDMA). For all following analyses, the metabolite concentrations were log2-transformed.

### 4.9. Statistical Analyses

For the patients in the discovery cohort (n = 368), general characteristics, also stratified by sex, were reported as medians with 25th and 75th quartiles (continuous data) or as proportions (nominal data) (Table 1). In linear regression models, associations between LVEF, LVEDD^measured^, and ^LVEDDacc. to HENRY^ (exposures), as well as all proteins or metabolites (outcomes), were assessed. Outcome and exposure variables were z-transformed. We first calculated a combined model including men and women and, subsequently, models stratified by sex. Combined models for LVEF and LVEDD^measured^ were adjusted for sex, age, and BMI, as these covariates have a major impact on heart structure and function as well as on metabolites and proteins. Combined models for LVEDD^acc. to HENRY^ were adjusted for sex only, as age and body surface area were already included in the Henry formula. In sex-specific models, the adjustment for sex was omitted. Differences in effect estimates between men and women were examined using scatterplots. As the results of the analyses for LVEDD^measured^ and LVEDD^acc. to HENRY^ were comparable, we only reported the results for LVEDD^acc. to HENRY^. We additionally tested whether any of the examined associations were non-linear using restricted cubic splines with four knots pre-specified at the 5th, 35th, 65th, and 95th percentiles [62]. The non-linear model was preferred to the linear model if the likelihood ratio test indicated that the fit of the non-linear model was significantly better than the fit of the linear model. To account for multiple testing, we adjusted the *p*-values by controlling the false discovery rate (FDR) at 5% using the Benjamini–Hochberg procedure [63]. FDR values < 0.05 were considered statistically significant.

In a sensitivity analysis, we additionally adjusted the models assessing the associations between LVEF or LVEDD^acc. to HENRY^ and the proteins or metabolites for ASAT activity as a proxy for liver function. Finally, the relations between all proteins and metabolites that demonstrated significant associations with LVEF were inspected using Spearman correlation. The proteins and metabolites that had at least one significant correlation with r < −0.3 or r > 0.3 were subject to a cluster analysis to determine underlying patterns of association. The clustering was based on the average linkage method.

In the validation cohort, we examined the associations between LVEF and the significant metabolites in men from the smaller DCM cohort from Heidelberg. Models were specified as reported above, and the results were corrected for multiple testing. Effect estimates of the discovery and the validation cohort were compared using scatterplots.

All statistical analyses were performed with SAS 9.4 (SAS Institute Inc., Cary, NC, USA).

## Figures and Tables

**Table 1:** Characteristics of the 368 DCM patients in the discovery cohort, stratified by sex.

Characteristics	Total Sample (n = 368)	Male (n = 297)	Female (n = 71)
Age, years	54.8 (47.9–63.7)	54.9 (48.2–63.5)	54.4 (46.8–65.7)
BMI, kg/m^2^	28.0 (25.1–31.2)	28.4 (25.6–31.6)	26.0 (22.8–29.4)
Diabetes mellitus, %	17.1	18.2	12.7
Current smoking, % *	30.8	30.7	31.0
LVEF, %	30.8 (25.0–37.0)	30.0 (25.0–36.0)	33.0 (27.0–37.5)
HFrEF, % *	76.6	76.8	75.8
LVEDD^measured^, mm *	68.0 (63.0–73.0)	69.0 (64.0–74.0)	63.0 (60.0–69.0)
LVEDD^acc. to HENRY^, % *	139 (129–149)	140 (130–150)	135 (127–146)
NYHA Class, %			
I	14.1	14.5	12.7
II	34.0	32.7	39.4
III	40.2	40.7	38.0
IV	3.30	3.37	2.80
missing	8.40	8.80	7.00
MAGGIC score	17.0 (13.0–21.0)	17.0 (13.0–21.0)	15.0 (11.0–19.0)
Symptom time, days *	113 (33–489)	112 (33–459)	131 (35–804)
Myocardial inflammation, %	41.8	40.1	49.3
Medication intake, %			
ACE inhibitors or AT1 antagonists	98.8	99.7	95.8
Beta blockers	97.0	98.3	91.6
Aldosterone antagonists	53.8	55.2	47.9
Diuretics	84.0	86.9	71.8
Digitalis	22.3	23.9	15.5
History of, %			
Stroke	6.00	7.10	1.40
Atrial fibrillation	24.5	27.6	11.3
Cancer	4.90	4.70	5.60
COPD *	8.17	8.50	7.00
hsCRP, mg/L *	6.92 (6.58–7.31)	6.91 (6.57–7.31)	7.05 (6.60–7.33)
ASAT, µkatal/L *	0.43 (0.32–0.62)	0.44 (0.34–0.63)	0.34 (0.27–0.59)
Total cholesterol, mmol/L *	5.10 (4.20–6.00)	5.00 (4.20–5.90)	5.35 (4.50–6.20)
HDL-cholesterol, mmol/L *	1.05 (0.85–1.30)	1.00 (0.80–1.23)	1.34 (1.15–1.56)
LDL-cholesterol, mmol/L *	3.15 (2.50–3.89)	3.13 (2.48–3.90)	3.19 (2.62–3.87)
eGFR, mL/min/1.73 m^2^	83.9 (69.2–98.0)	83.7 (70.2–96.8)	89.2 (66.3–103.1)
Chronic kidney disease, %			
mild to moderate	15.2	14.5	18.3
severe	0.27	0.34	0.00

Data are proportions or medians (1st–3rd quartile). HFrEF was defined as LVEF ≤ 40% and NYHA II–IV. Myocardial inflammation was present if immunohistochemistry revealed focal or diffuse mononuclear infiltrates with more than 14 leucocytes per square millimeter (CD3+ T-lymphocytes and/or CD68+ macrophages) in addition to enhanced expression of HLA class II molecules [14]. The eGFR was calculated according to the CKD-EPI creatinine and cystatin C formula. eGFR 30–59 mL/min/1.73 m^2^ values were defined as mild to moderate and <30 mL/min/1.73 m^2^ as severe chronic kidney disease. * Missing data in men/women, respectively: smoking n = 7/0; HFrEF 26/5; LVEDD^measured^ and LVEDD^acc. to HENRY^ n = 2/0; symptom time n = 1/2; cancer n = 1/0; COPD n = 1/0; hsCRP n = 18/9; ASAT n = 29/8; cholesterol n = 29/5; HDL-cholesterol n = 37/6; LDL-cholesterol n = 35/. ACE, angiotensin-converting enzyme; AT1 antagonists, angiotensin I antagonists; BMI, body mass index; COPD, chronic obstructive pulmonary disease; eGFR, estimated glomerular filtration rate; HDL-cholesterol, high-density lipoprotein cholesterol; HFrEF, heart failure with reduced ejection fraction; hsCRP, high-sensitivity C-reactive protein; LDL-cholesterol, low-density lipoprotein cholesterol; LVEDD, left ventricular end diastolic diameter; LVEF, left ventricular ejection fraction; MAGGIC score, meta-analysis global group in chronic heart failure risk score; NYHA, New York Heart Association Classification.

## Data Availability

Restrictions apply to the availability of data generated or analyzed during this study to preserve patient confidentiality or because they were used under license.

## Supplementary Materials

The following supporting information can be downloaded at: https://www.mdpi.com/article/10.3390/ijms25136827/s1.
