# Supplementary material for: Integrative Analyses of Circulating Proteins and Metabolites Reveal Sex Differences in the Associations with Cardiac Function among DCM Patients
Source: Int J Mol Sci. 2024 Jun 21;25(13):6827. doi: 10.3390/ijms25136827 (PMC11241450; doi:10.3390/ijms25136827)
Supplement: Supplementary file 1 [file ijms-25-06827-s001.zip › ijms-3023806-supplementary.pdf]

Supplemental Material

Supplemental Figure S1: Scatterplots for circulating ST2, osteopontin, galectin 3 and MMP2 concentrations measured by ELISA and the targeted (Olink) or untargeted (LC-MS/MS) approach

Supplemental Figure S2: Heatmap illustrating the associations between left ventricular ejection fraction (LVEF) or left ventricular end diastolic diameter ($LVEDD^{acc.\ to\ HENRY}$) and the plasma proteins quantified by the targeted approach (Olink)

Supplemental Figure S3: Heatmap illustrating the associations between left ventricular ejection fraction (LVEF) or left ventricular end diastolic diameter ($LVEDD^{acc.\ to\ HENRY}$) and the proteins quantified by the untargeted approach

Supplemental Figure S4: Heatmap illustrating the associations between left ventricular ejection fraction (LVEF) or left ventricular end diastolic diameter ($LVEDD^{acc.\ to\ HENRY}$) and the metabolites

Supplemental Figure S5: Scatterplots contrasting the effect estimates for the associations between LVEF and the metabolites or proteins for men and women obtained from linear regression models

Supplemental Figure S6: Heatmap illustrating the associations between left ventricular ejection fraction (LVEF) and selected metabolites in male DCM patients from the discovery (Greifswald n=297) and validation (Heidelberg n=93) cohorts

Supplemental Figure S7: Scatterplots contrasting the effect estimates for the associations between LVEF and the metabolites for men from the discovery and validation cohort

Supplemental Figure S8: Scatterplot illustrating the correlation between left ventricular ejection fraction (LVEF) and left ventricular end diastolic diameter ($LVEDD^{acc.\ to\ HENRY}$) in the discovery cohort (n=368)

Supplemental Figure S9: Schematic representation of the categories and concentration ranges of the plasma proteome covered by tandem mass spectrometry and proximity ligation assay;

Supplemental Table S1A: LC-MS/MS parameter (data dependent mode; quantitative data)

Supplemental Table S1B: MaxQuant parameters for peptide/protein identification and intensity extraction

Supplemental Table S2: Associations of left ventricular ejection fraction (LVEF) and the plasma proteins measured using LC-MS/MS (untargeted approach)

Supplemental Table S3: Associations of left ventricular end diastolic diameter ($LVEDD^{acc.\ to\ HENRY}$) and the plasma proteins measured using LC-MS/MS (untargeted approach)

Supplemental Table S4: Associations of left ventricular ejection fraction (LVEF) and the plasma proteins measured using Olink (targeted approach)

Supplemental Table S5: Associations of left ventricular end diastolic diameter ($LVEDD^{acc.\ to\ HENRY}$) and the plasma proteins measured using Olink (targeted approach)

Supplemental Table S6: Associations of left ventricular ejection fraction (LVEF) and the metabolites

Supplemental Table S7: Associations of left ventricular end diastolic diameter ($LVEDD^{acc.\ to\ HENRY}$) and the metabolites

Supplemental Figures

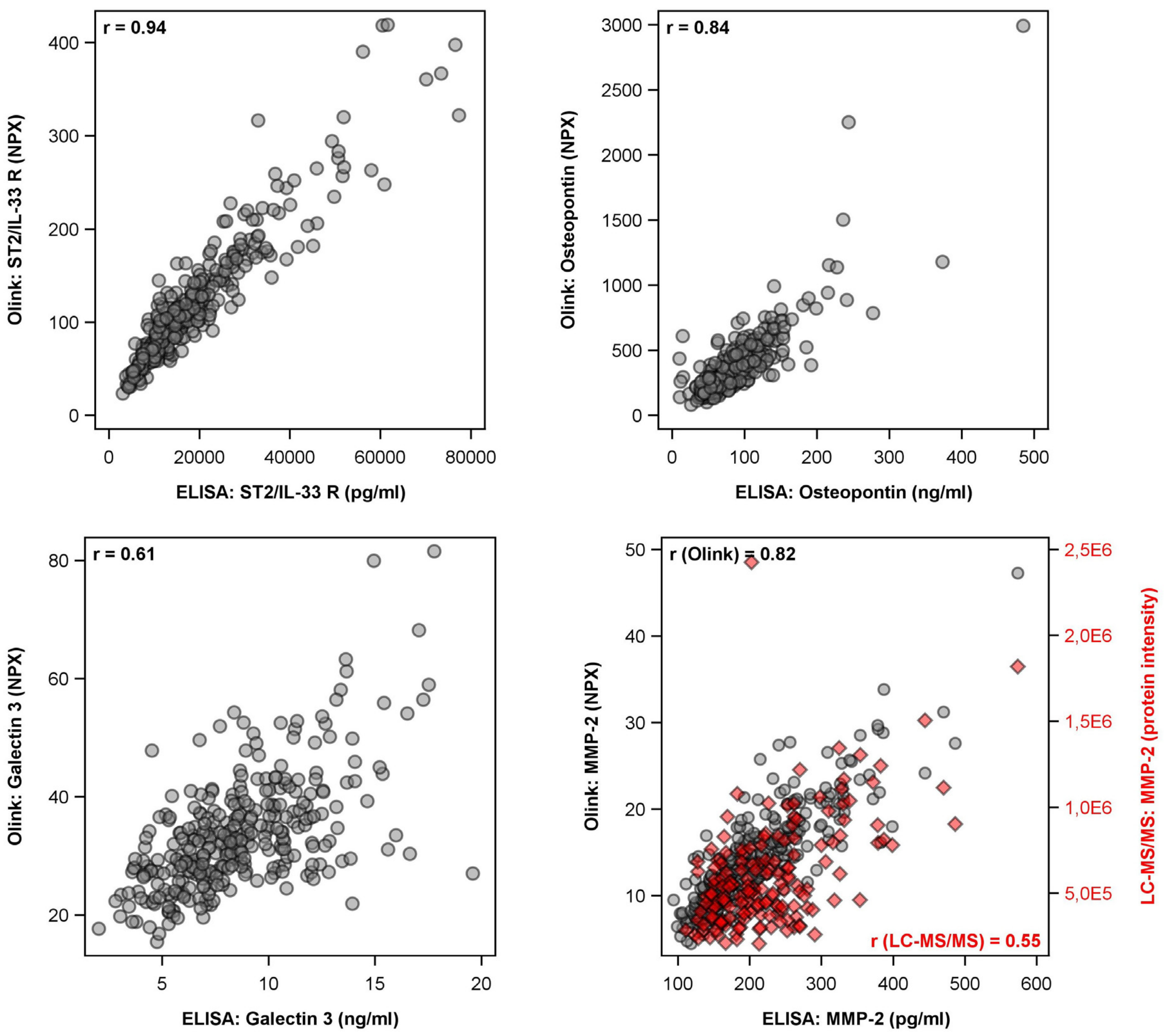

Supplemental Figure S1. Scatterplots for circulating ST2, osteopontin, galectin 3 and MMP2 concentrations measured by ELISA and the targeted (Olink) or untargeted (LC-MS/MS) approach. Pearson correlation coefficients are reported.

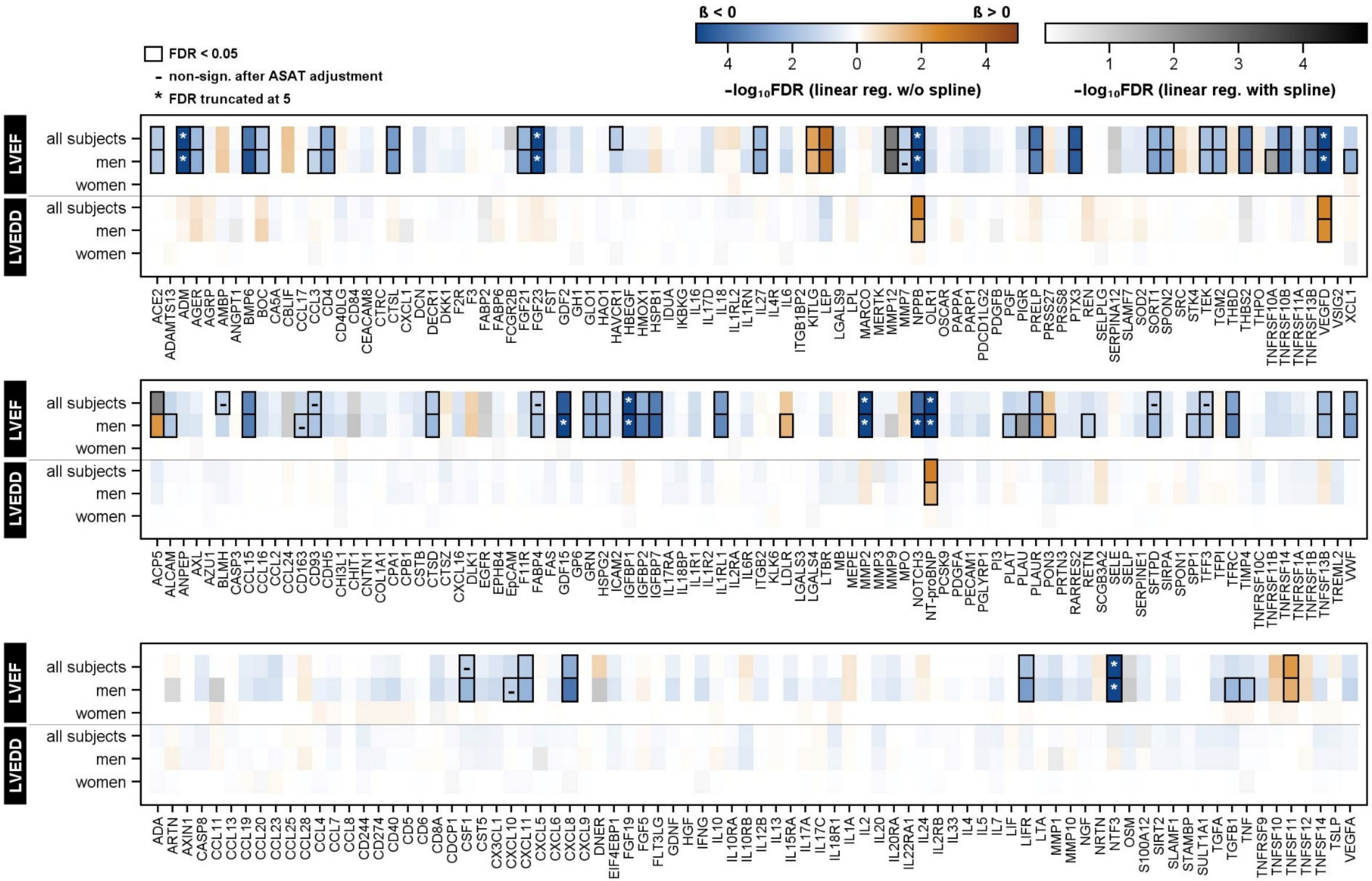

Supplemental Figure S2. Heatmap illustrating the associations between left ventricular ejection fraction (LVEF) or left ventricular end diastolic diameter (LVEDD= LVEDD$^{\text{acc. to HENRY}}$) and the plasma proteins quantified by the targeted approach (Olink). Results from regression models adjusted for sex, age and BMI for the whole study population and from sex-specific models adjusted for age and BMI. The color gradient illustrates the false discovery rate (FDR) obtained after correction for multiple testing. A blue-to-orange color gradient highlights a linear association, with blue color indicating an inverse and orange color a positive association. A white-to-black color gradient highlights a non-linear association. A black frame indicates a significant result (FDR<0.05). A black point within a box implies that the association is no longer significant after additional adjustment for ASAT activity. An asterisk within a box implies that the FDR was truncated at a value of 5. Ten proteins were present on the inflammation and CVD II or III panel. To reduce redundancies, the results from the inflammation panel were not illustrated.

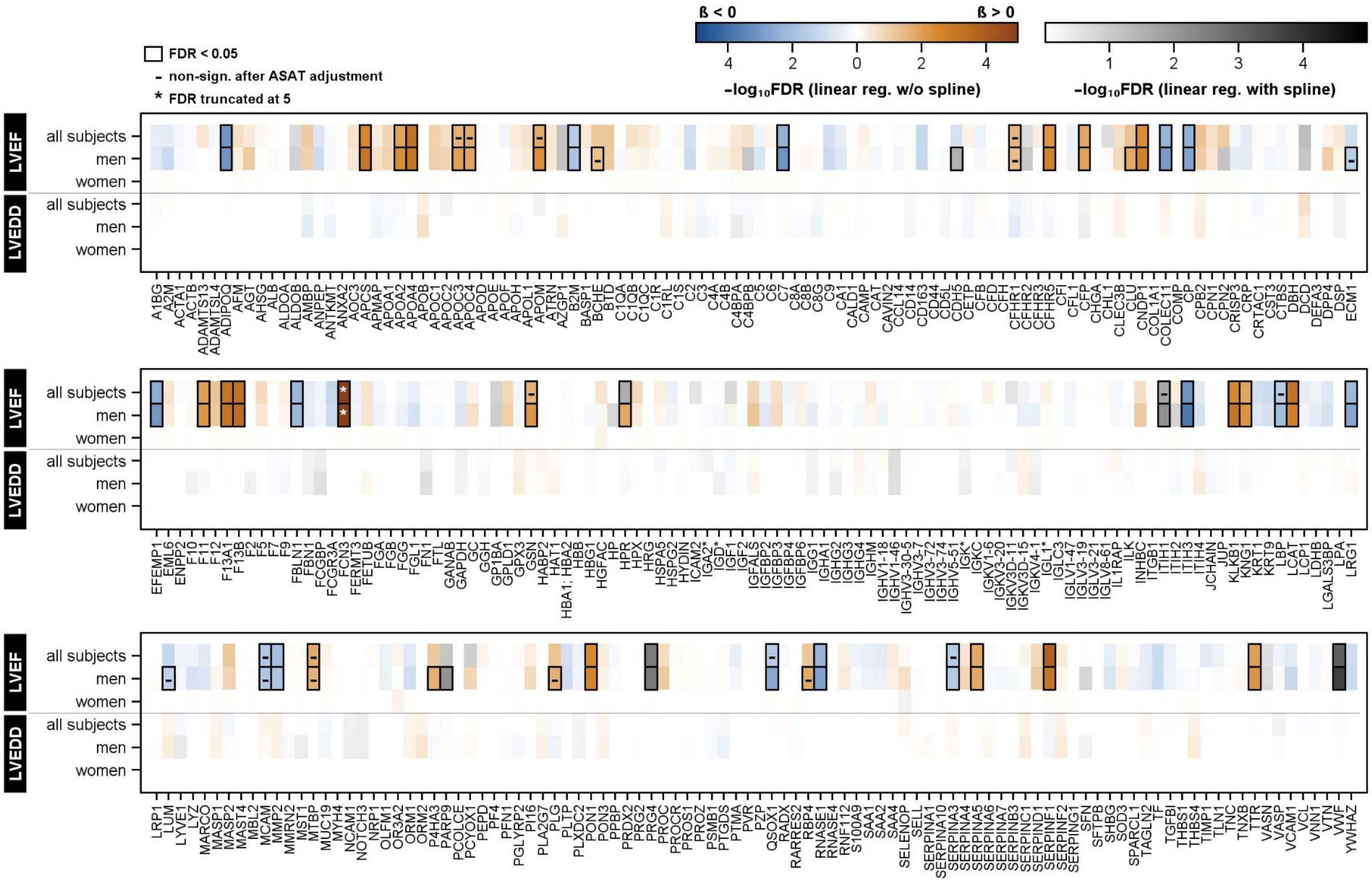

Supplemental Figure S3. Heatmap illustrating the associations between left ventricular ejection fraction (LVEF) or left ventricular end diastolic diameter (LVEDD = LVEDD$^{\text{acc. to HENRY}}$) and the proteins quantified by the untargeted approach. Results from regression models adjusted for sex, age and BMI for the whole study population and from sex-specific models adjusted for age and BMI. The color gradient illustrates the false discovery rate (FDR) obtained after correction for multiple testing. A blue-to-orange color gradient highlights a linear association, with blue color indicating an inverse and orange color a positive association. A white-to-black color gradient highlights a non-linear association. A black frame indicates a significant result (FDR<0.05). A black point within a box implies that the association is no longer significant after additional adjustment for ASAT activity. An asterisk within a box implies that the FDR was truncated at a value of 5.

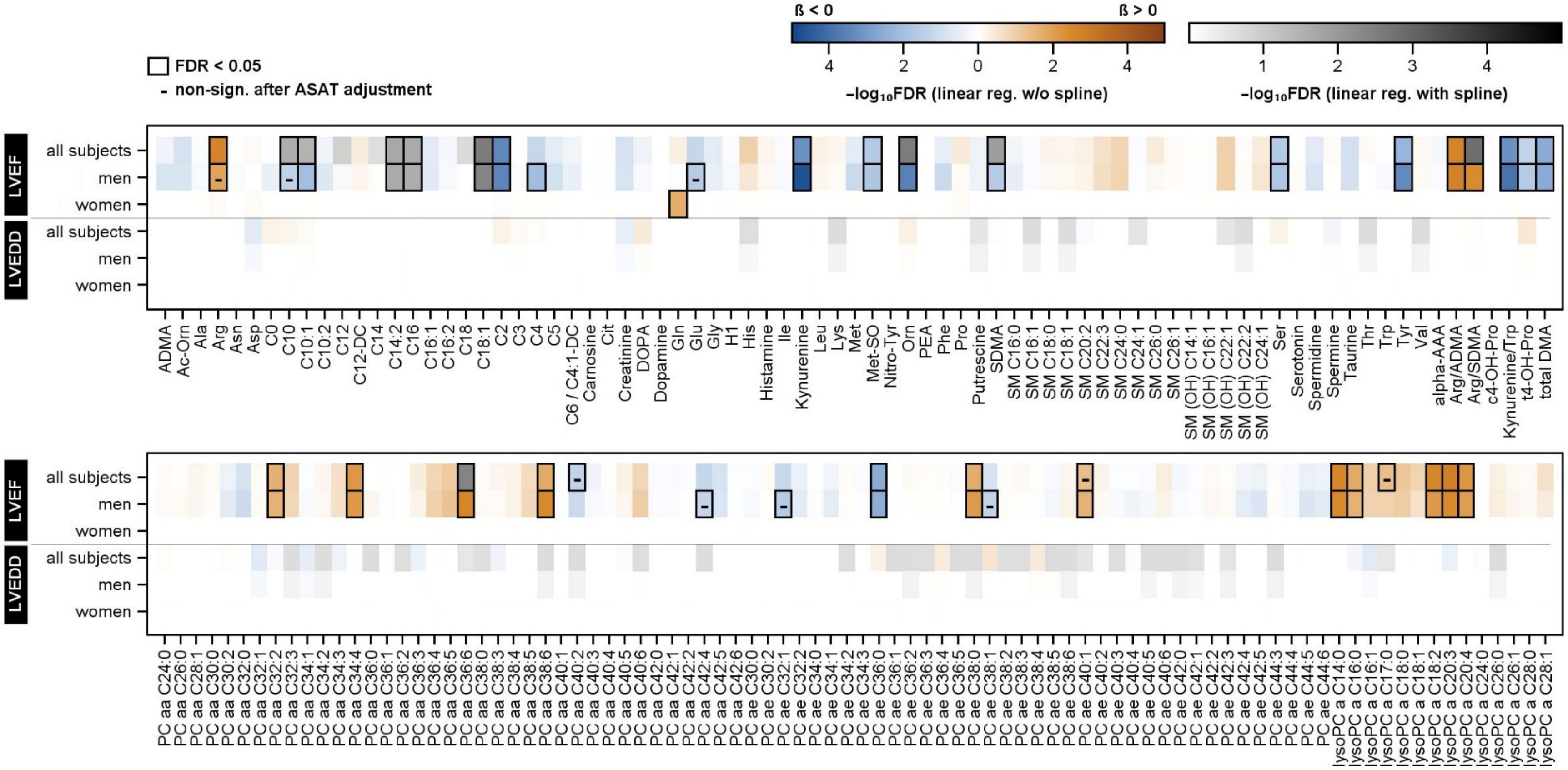

Supplemental Figure S4. Heatmap illustrating the associations between left ventricular ejection fraction (LVEF) or left ventricular end diastolic diameter (LVEDD = LVEDD$^{\text{acc. to HENRY}}$) and the metabolites. Results from regression models adjusted for sex, age and BMI for the whole study population and from sex-specific models adjusted for age and BMI. The color gradient illustrates the false discovery rate (FDR) obtained after correction for multiple testing. A blue-to-orange color gradient highlights a linear association, with blue color indicating an inverse and orange color a positive association. A white-to-black color gradient highlights a non-linear association. A black frame indicates a significant result (FDR<0.05). A black point within a box implies that the association is no longer significant after additional adjustment for ASAT activity.

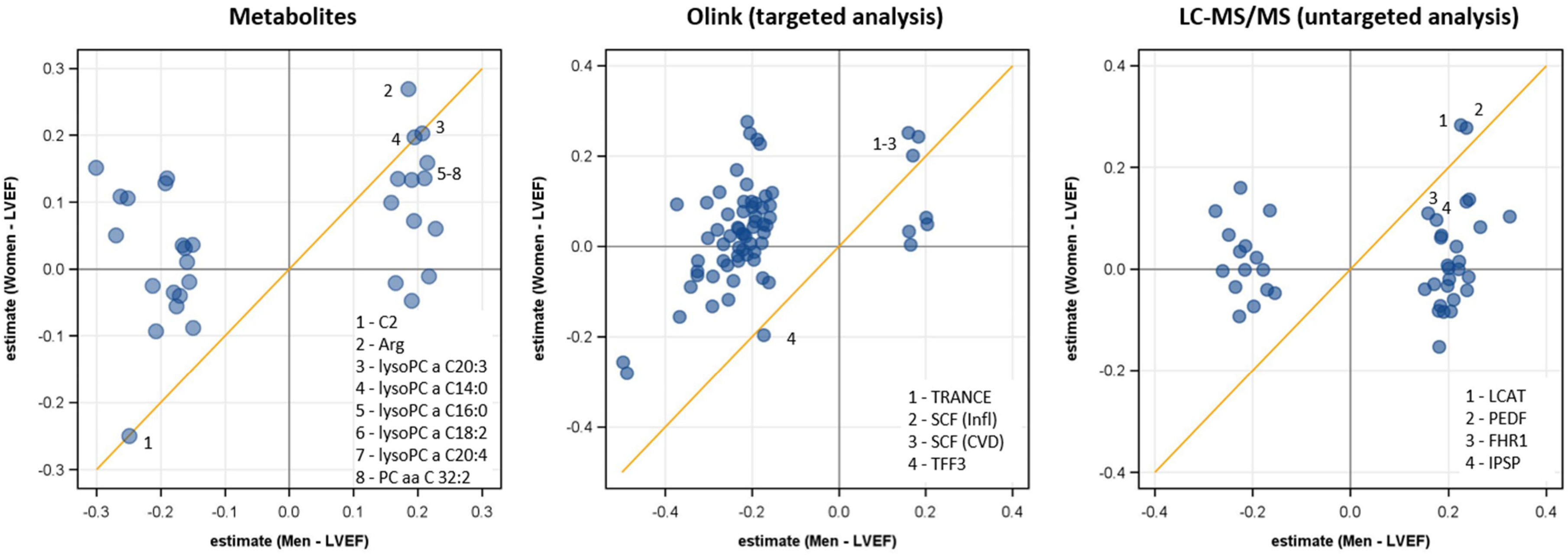

Supplemental Figure S5. Scatterplots contrasting the effect estimates for the associations between LVEF and the metabolites or proteins for men and women obtained from linear regression models. Only metabolites and proteins with significant associations to LVEF in men are displayed. Labelled metabolites and proteins close to the yellow line have similar effects in both sexes. The statistical power of the female sample (71 women vs. 297 men) might have been too small for these effects to become statistically significant. Other metabolites or proteins further away from the yellow line, indicate sex-differences. Points close to the ‚0‘ on the y-axis indicated absence of effects in women. Points further away from the ‚0‘ on the y-axis indicate opposite effects in men and women, which, again, might not have turned significant due to the smaller statistical power in women.

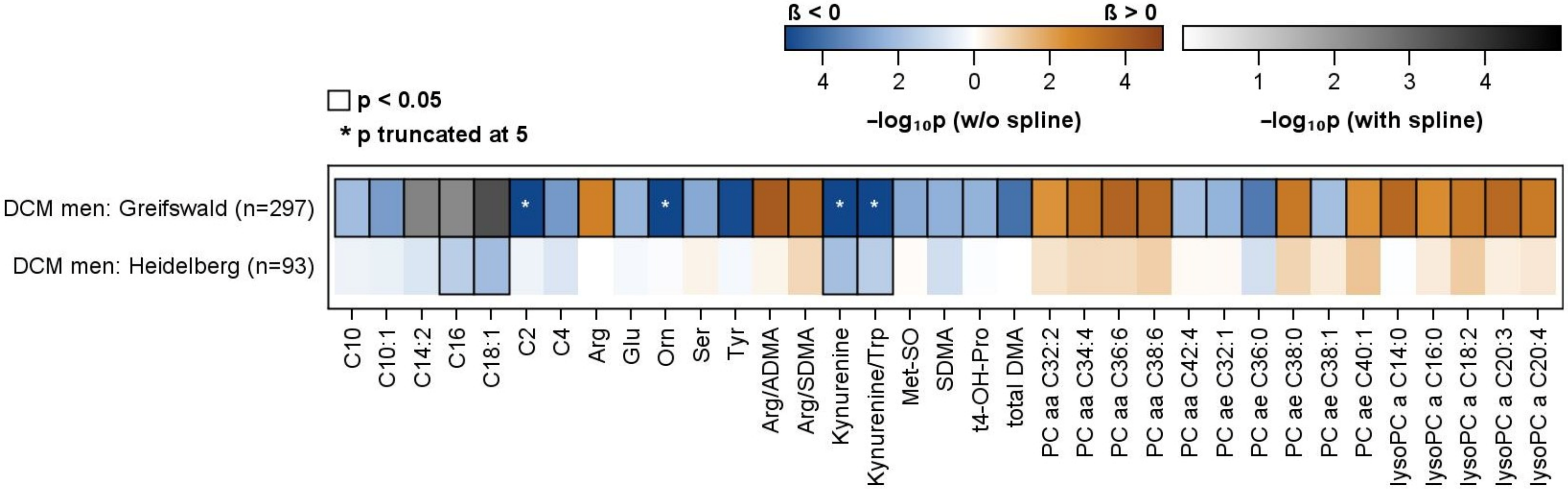

Supplemental Figure S6. Heatmap illustrating the associations between left ventricular ejection fraction (LVEF) and selected metabolites in male DCM patients from the discovery (Greifswald n=297) and validation (Heidelberg n=93) cohorts. Only those metabolites that were significantly associated with LVEF (FDR<0.05) in the discovery cohort were selected for validation and included in the analyses. The color gradient illustrates the p-values from separate regression models adjusted for age and BMI. A blue-to-orange color gradient highlights a linear association, with blue color indicating an inverse and orange color a positive association. A white-to-black color gradient highlights a non-linear association. A black frame indicates a significant result (p<0.05). An asterisk within a box implies that the p-value was truncated at a value of 5.

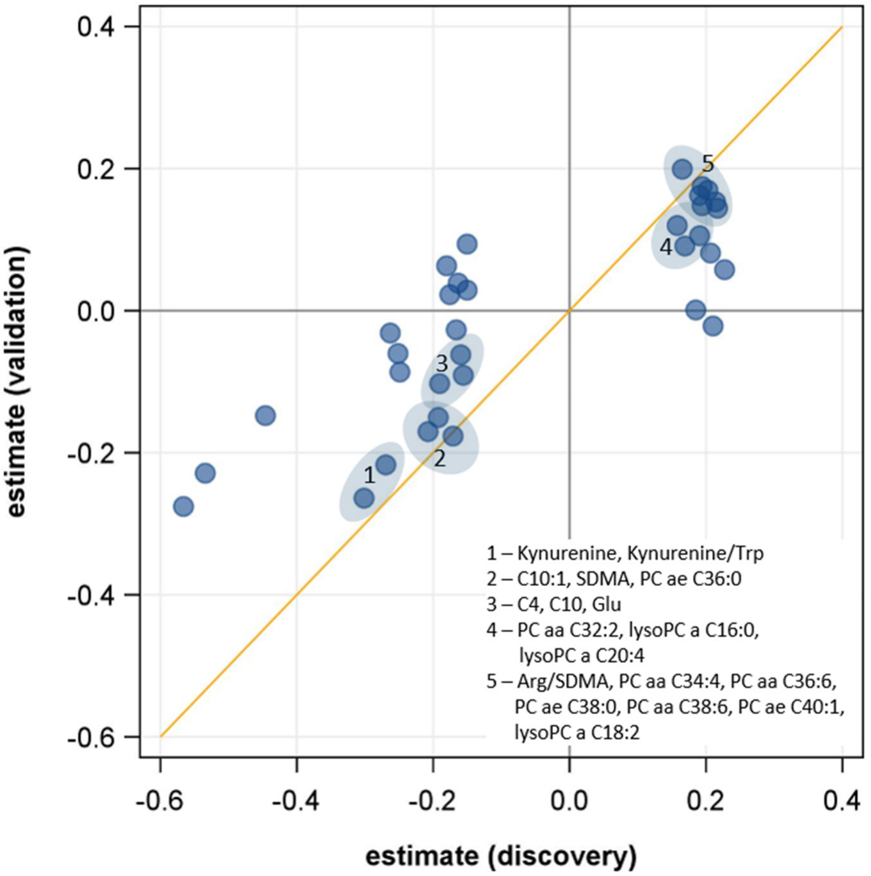

Supplemental Figure S7. Scatterplots contrasting the effect estimates for the associations between LVEF and the metabolites for men from the discovery and validation cohort. Results obtained from linear regression models. Only metabolites with significant associations to LVEF in men in the discovery cohort are displayed. Labelled metabolites close to the yellow line have similar effects in both cohorts. The statistical power of the validation sample (93 men in the validation cohort vs. 297 men in the discovery cohort) might have been too small for these effects to become statistically significant. Other metabolites further away from the yellow line, indicate differences between the discovery and validation cohort. Points close to the ‚0‘ on the y-axis indicated absence of effects in the validation cohort.

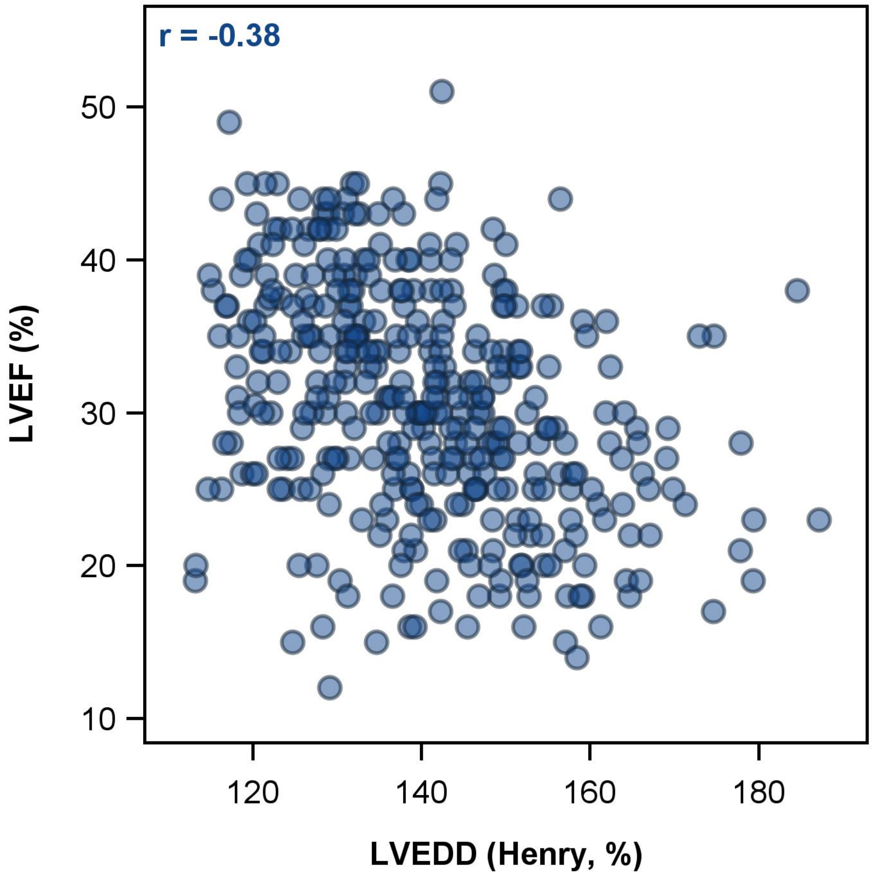

Supplemental Figure S8. Scatterplot illustrating the correlation between left ventricular ejection fraction (LVEF) and left ventricular end diastolic diameter (LVEDD$^{acc.\ to\ HENRY}$) in the discovery cohort (n=368). The Pearson correlation coefficient is reported.

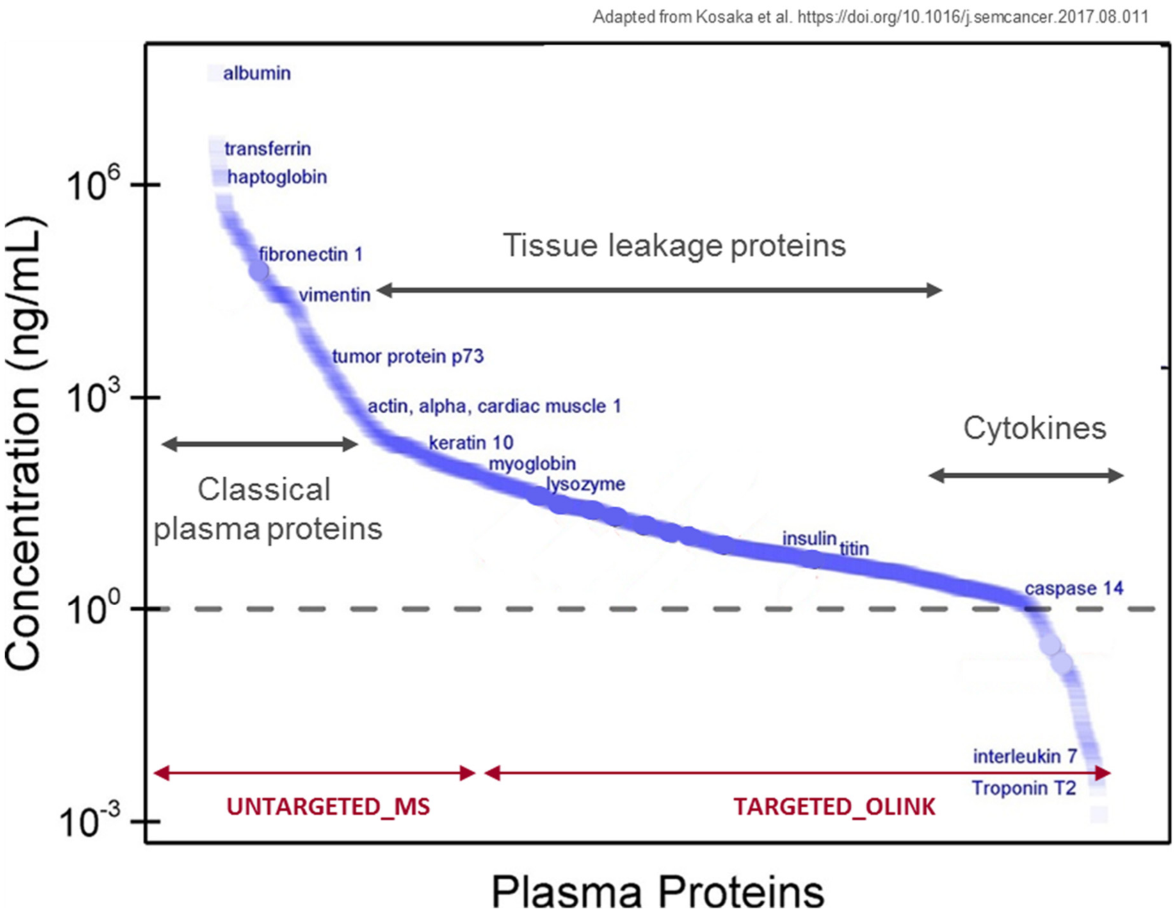

Supplemental Figure S9. Schematic representation of the categories and concentration ranges of the plasma proteome covered by tandem mass spectrometry and proximity ligation assay.

Supplemental Table S1A. LC-MS/MS parameter (data dependent mode; quantitative data)

Data dependent analyses (DDA)	
reversed phase liquid chromatography	**nano-Acquity UPLC (Waters Corp)**
trap column	nanoACQUITY UPLC Symmetry C18 Trap Column, 100Å, 5 µm, 180 µm x 20 mm, 2G, V/M, Waters
analytical column	BEH C18 nanoACQUITY Column 10K psi, 130Å, 1.7 µm, 100 µm X 100 mm, Waters
flow rate	400 nl/min
column oven temperature	40°C
buffer system	binary buffer system consisting of 0.1% acetic acid in HPLC-grade water (buffer A) and 100% ACN in 0.1% acetic acid (buffer B)
gradient	gradient of buffer B: 5min 1% to 5 %, 63min 5% to 25%, 25min 25 to 60%, 2min 60% to 99%, 2min 99%, 1min 99% to 1%, 5 min 1%
Mass spectrometer	**LTQ Orbitrab Velos**
operation mode	data-dependent
electrospray	Nanospray Ion Source
Full MS	
MS scan resolution	30,000
AGC target	1e6
maximum ion injection time for the MS scan	10 ms
scan range	325 to 1525 *m/z*
spectra data type	profile
dd-MS2	
resolution	7,500
MS/MS AGC target	3e4
minimum ACG target	-
intensity threshold	2e3
maximum ion injection time for the MS/MS scans	100 ms
number of MS/MS scans	Top 20
spectra data type	centroid
selection for MS/MS	1
isolation window	2.0 Da
fixed first mass	-
dissociation mode	collisional induced dissociation (CID)
normalized collision energy	35
charge exclusion	unassigned,1, >3
dynamic exclusion	60 sec

Supplemental Table S1B. MaxQuant parameters for peptide/protein identification and intensity extraction

MaxQuant

Parameter	*Value*
Version	2.0.1.0
PSM FDR	0.01
PSM FDR Crosslink	0.01
Protein FDR	0.01
Site FDR	0.01
Use Normalized Ratios For Occupancy	True
Min. peptide Length	6
Min. score for unmodified peptides	0
Min. score for modified peptides	40
Min. delta score for unmodified peptides	0
Min. delta score for modified peptides	6
Min. unique peptides	0
Min. razor peptides	1
Min. peptides	1
Use only unmodified peptides and	True
Modifications included in protein quantification	Oxidation (M);Acetyl (Protein N-term)
Peptides used for protein quantification	Razor
Discard unmodified counterpart peptides	True
Label min. ratio count	1
Use delta score	False
iBAQ	True
iBAQ log fit	True
Match between runs	True
Matching time window [min]	0.7
Match ion mobility window [indices]	0.05
Alignment time window [min]	20
Alignment ion mobility window [indices]	1
Find dependent peptides	False
Fasta file	human_uniprot_reviewed_20394sequences_210125.fasta
Decoy mode	revert
Include contaminants	True
Advanced ratios	True
Second peptides	True
Stabilize large LFQ ratios	True
Separate LFQ in parameter groups	False
Require MS/MS for LFQ comparisons	True
Calculate peak properties	False
Main search max. combinations	200

Advanced site intensities	True
Write msScans table	False
Write msmsScans table	False
Write ms3Scans table	False
Write allPeptides table	True
Write mzRange table	False
Max. peptide mass [Da]	4600
Min. peptide length for unspecific search	8
Max. peptide length for unspecific search	25
Razor protein FDR	True
Disable MD5	False
Max mods in site table	3
Match unidentified features	False
Da interval. (Unknown)	100
MS/MS deisotoping (Unknown)	True
MS/MS deisotoping tolerance (Unknown)	7
MS/MS deisotoping tolerance unit (Unknown)	ppm
MS/MS higher charges (Unknown)	True
MS/MS water loss (Unknown)	True
MS/MS ammonia loss (Unknown)	True
MS/MS dependent losses (Unknown)	True
MS/MS recalibration (Unknown)	False
Site tables	Oxidation (M)Sites.txt

Supplemental Table S2. Associations of left ventricular ejection fraction (LVEF) and the plasma proteins measured using LC-MS/MS (untargeted approach).
Results from regression models adjusted for sex, age and BMI for the whole study population and from sex-specific models adjusted for age and BMI.
Proteins were log10-transformed. Estimates are presented for z-transformed outcome and exposure variables. In the case of less than 50 observations per protein, no results are given.
stderr, standard error; FDR, false discovery rate

Outcome			Exposure	All subjects			Men			Women		
				beta	stderr	FDR	beta	stderr	FDR	beta	stderr	FDR
Entry name	Gene name	Protein name										
A1AG1	ORM1	Alpha-1-acid glycoprotein 1	LVEF	-0.102	0.053	0.199	-0.131	0.059	0.099	0.048	0.122	0.928
A1AG2	ORM2	Alpha-1-acid glycoprotein 2	LVEF	-0.012	0.053	0.939	-0.008	0.059	0.945	-0.010	0.123	0.991
A1AT	SERPINA1	Alpha-1-antitrypsin	LVEF	-0.014	0.053	0.939	-0.011	0.059	0.919	-0.024	0.122	0.962
A1BG	A1BG	Alpha-1B-glycoprotein	LVEF	-0.085	0.052	0.286	-0.106	0.059	0.204	0.055	0.121	0.928
A2AP	SERPINF2	Alpha-2-antiplasmin	LVEF	0.068	0.049	0.393	0.050	0.055	0.606	0.182	0.116	0.910
A2GL	LRG1	Leucine-rich alpha-2-glycoprotein	LVEF	**-0.179**	**0.050**	**0.006**	**-0.215**	**0.056**	**0.002**	0.046	0.118	0.928
A2MG	A2M	Alpha-2-macroglobulin	LVEF	-0.083	0.050	0.281	-0.137	0.055	0.071	0.175	0.117	0.910
AACT	SERPINA3	Alpha-1-antichymotrypsin	LVEF	**-0.155**	**0.052**	**0.025**	**-0.170**	**0.058**	**0.023**	-0.041	0.120	0.928
ACTB	ACTB	Actin, cytoplasmic 1	LVEF	-0.041	0.053	0.699	-0.037	0.059	0.747	-0.070	0.120	0.925
ACTS	ACTA1	Actin, alpha skeletal muscle	LVEF	-0.059	0.053	0.519	-0.049	0.059	0.642	-0.110	0.120	0.910
ADIPO	ADIPOQ	Adiponectin	LVEF	**-0.211**	**0.049**	**0.001**	**-0.235**	**0.055**	**0.001**	-0.036	0.114	0.937
AFAM	AFM	Afamin	LVEF	0.100	0.052	0.203	0.071	0.057	0.446	0.181	0.121	0.910
ALBU	ALB	Albumin	LVEF	0.018	0.053	0.925	0.032	0.059	0.798	-0.009	0.123	0.991
ALDOA	ALDOA	Fructose-bisphosphate aldolase A	LVEF	0.005	0.101	0.990	-0.047	0.115	0.856	n < 50		
ALDOB	ALDOB	Fructose-bisphosphate aldolase B	LVEF	0.975	0.348	0.189	1.030	0.356	0.128	n < 50		
			LVEF'	-0.253	0.093		-0.284	0.100				
			LVEF''	0.795	0.317		0.904	0.342				
ALS	IGFALS	Insulin-like growth factor-binding protein complex acid labile subunit	LVEF	0.100	0.047	0.141	0.136	0.053	0.060	-0.080	0.099	0.910
AMBP	AMBP	Protein AMBP [Cleaved into: Alpha-1-microglobulin	LVEF	0.122	0.050	0.080	0.122	0.056	0.112	0.091	0.117	0.910
AMPN	ANPEP	Aminopeptidase N	LVEF	-0.136	0.068	0.186	-0.174	0.073	0.077	n < 50		
ANGT	AGT	Angiotensinogen	LVEF	0.070	0.050	0.385	0.140	0.059	0.077	-0.089	0.114	0.910
ANKMT	ANTKMT	Adenine nucleotide translocase lysine N-methyltransferase	LVEF	-0.056	0.065	0.651	-0.059	0.071	0.642	n < 50		
ANT3	SERPINC1	Antithrombin-III	LVEF	0.050	0.052	0.583	0.020	0.058	0.878	0.180	0.115	0.910
ANXA2	ANXA2	Annexin A2	LVEF	0.011	0.055	0.948	-0.007	0.060	0.946	0.156	0.127	0.910
AOC3	AOC3	Membrane primary amine oxidase	LVEF	0.167	0.095	0.252	0.156	0.106	0.340	n < 50		
APMAP	APMAP	Adipocyte plasma membrane-associated protein	LVEF	0.106	0.079	0.405	0.147	0.087	0.244	n < 50		
APOA	LPA	Apolipoprotein	LVEF	0.022	0.054	0.897	0.021	0.060	0.878	0.035	0.122	0.943
APOA1	APOA1	Apolipoprotein A-I	LVEF	0.108	0.050	0.138	0.120	0.057	0.124	0.023	0.114	0.962
APOA2	APOA2	Apolipoprotein A-II	LVEF	**0.169**	**0.051**	**0.013**	**0.210**	**0.058**	**0.004**	-0.061	0.102	0.925
APOA4	APOA4	Apolipoprotein A-IV	LVEF	**0.226**	**0.050**	**0.001**	**0.237**	**0.055**	**0.001**	0.133	0.119	0.910
APOB	APOB	Apolipoprotein B-100	LVEF	-0.006	0.052	0.975	-0.003	0.058	0.984	0.020	0.118	0.973
APOC1	APOC1	Apolipoprotein C-I	LVEF	0.121	0.053	0.104	0.130	0.059	0.102	0.091	0.121	0.910
APOC2	APOC2	Apolipoprotein C-II	LVEF	0.097	0.052	0.215	0.117	0.058	0.140	0.029	0.117	0.950
APOC3	APOC3	Apolipoprotein C-III	LVEF	**0.154**	**0.053**	**0.027**	**0.190**	**0.058**	**0.011**	-0.085	0.122	0.910
APOC4	APOC4	Apolipoprotein C-IV	LVEF	**0.164**	**0.052**	**0.018**	**0.185**	**0.058**	**0.013**	0.062	0.120	0.928
APOD	APOD	Apolipoprotein D	LVEF	-0.036	0.051	0.752	-0.026	0.055	0.816	-0.085	0.120	0.910
APOE	APOE	Apolipoprotein E	LVEF	0.021	0.053	0.901	0.014	0.059	0.903	0.093	0.119	0.910
APOF	APOF	Apolipoprotein F	LVEF	-0.059	0.050	0.487	-0.024	0.056	0.851	-0.188	0.118	0.910
APOH	APOH	Beta-2-glycoprotein 1	LVEF	0.074	0.053	0.391	0.065	0.058	0.517	0.051	0.119	0.928
APOL1	APOL1	Apolipoprotein L1	LVEF	0.083	0.049	0.268	0.080	0.057	0.365	0.085	0.120	0.910
APOM	APOM	Apolipoprotein M	LVEF	**0.158**	**0.051**	**0.019**	**0.205**	**0.057**	**0.005**	-0.084	0.117	0.910
ATL4	ADAMTSL4	ADAMTS-like protein 4	LVEF	-0.059	0.058	0.564	-0.060	0.065	0.606	-0.031	0.129	0.951
ATRN	ATRN	Attractin	LVEF	0.084	0.053	0.303	0.087	0.058	0.329	0.018	0.122	0.983
ATS13	ADAMTS13	A disintegrin and metalloproteinase with thrombospondin motifs 13	LVEF	0.231	0.146	0.311	n < 50			n < 50		
B2MG	B2M	Beta-2-microglobulin [Cleaved into: Beta-2-microglobulin form pI 5.3]	LVEF	**-0.148**	**0.049**	**0.023**	**-0.178**	**0.056**	**0.013**	-0.001	0.102	0.994
BASP1	BASP1	Brain acid soluble protein 1	LVEF	0.127	0.088	0.370	0.103	0.100	0.558	n < 50		
BGH3	TGFBI	Transforming growth factor-beta-induced protein ig-h3	LVEF	-0.076	0.053	0.370	-0.078	0.059	0.417	-0.096	0.119	0.910
BIP	HSPA5	Endoplasmic reticulum chaperone BiP	LVEF	0.116	0.091	0.428	0.081	0.100	0.642	n < 50		
BTD	BTD	Biotinidase	LVEF	0.133	0.052	0.067	0.140	0.058	0.074	0.097	0.118	0.910
C163A	CD163	Scavenger receptor cysteine-rich type 1 protein M130	LVEF	-0.160	0.061	0.055	-0.175	0.067	0.055	-0.138	0.147	0.910
C1QA	C1QA	Complement C1q subcomponent subunit A	LVEF	-0.017	0.052	0.926	-0.053	0.059	0.606	0.119	0.121	0.910
C1QB	C1QB	Complement C1q subcomponent subunit B	LVEF	0.091	0.053	0.252	0.051	0.059	0.623	0.243	0.118	0.910
C1QC	C1QC	Complement C1q subcomponent subunit C	LVEF	0.077	0.053	0.359	0.061	0.059	0.550	0.154	0.121	0.910
C1R	C1R	Complement C1r subcomponent	LVEF	0.054	0.053	0.549	0.045	0.059	0.676	0.099	0.121	0.910
C1RL	C1RL	Complement C1r subcomponent-like protein	LVEF	-0.025	0.053	0.855	-0.037	0.059	0.747	-0.520	0.457	0.910
			LVEF'							0.278	0.149	
			LVEF''							-0.903	0.423	
C1S	C1S	Complement C1s subcomponent	LVEF	0.041	0.053	0.696	0.015	0.059	0.895	0.166	0.120	0.910
C4BPA	C4BPA	C4b-binding protein alpha chain	LVEF	0.080	0.053	0.325	0.093	0.059	0.285	0.059	0.107	0.925
C4BPB	C4BPB	C4b-binding protein beta chain	LVEF	0.093	0.052	0.242	0.514	0.230	0.077	0.056	0.108	0.928
			LVEF'				-0.071	0.066				
			LVEF''				0.129	0.228				
CADH5	CDH5	Cadherin-5	LVEF	-0.116	0.054	0.134	**-0.545**	**0.231**	**0.028**	-0.520	0.536	0.910
			LVEF'				**0.171**	**0.067**		-0.015	0.167	
			LVEF''				**-0.654**	**0.232**		0.271	0.468	
CAH1	CA1	Carbonic anhydrase 1	LVEF	-0.087	0.054	0.286	-0.112	0.060	0.183	0.035	0.123	0.943
CALD1	CALD1	Caldesmon	LVEF	0.012	0.093	0.975	0.034	0.100	0.882	n < 50		
CAMP	CAMP	Cathelicidin antimicrobial peptide	LVEF	0.056	0.055	0.559	0.045	0.061	0.687	0.092	0.128	0.910
CATA	CAT	Catalase	LVEF	-0.035	0.068	0.829	-0.072	0.074	0.586	n < 50		
CAVN2	CAVIN2	Caveolae-associated protein 2	LVEF	-0.030	0.128	0.939	n < 50			n < 50		
CBG	SERPINA6	Corticosteroid-binding globulin	LVEF	-0.044	0.050	0.644	-0.057	0.059	0.588	-0.082	0.107	0.910
CBPB2	CPB2	Carboxypeptidase B2	LVEF	0.139	0.053	0.055	0.136	0.059	0.083	0.101	0.117	0.910
CBPN	CPN1	Carboxypeptidase N catalytic chain	LVEF	0.094	0.051	0.225	0.092	0.058	0.286	0.103	0.121	0.910
CCL14	CCL14	C-C motif chemokine 14	LVEF	-0.091	0.130	0.754	-0.161	0.139	0.501	n < 50		
CD14	CD14	Monocyte differentiation antigen CD14	LVEF	0.024	0.050	0.855	0.023	0.057	0.856	0.025	0.108	0.956
CD44	CD44	CD44 antigen	LVEF	0.010	0.063	0.967	-0.039	0.071	0.796	n < 50		
CD5L	CD5L	CD5 antigen-like	LVEF	0.007	0.052	0.974	-0.045	0.058	0.672	0.205	0.113	0.910

CERU	CP	Ceruloplasmin	LVEF	**-0.191**	**0.051**	**0.004**	**-0.227**	**0.057**	**0.002**	-0.094	0.118	0.910
CETP	CETP	Cholesteryl ester transfer protein	LVEF	0.103	0.096	0.542	0.031	0.108	0.895	n < 50		
CFAB	CFB	Complement factor B	LVEF	-0.071	0.053	0.404	-0.048	0.059	0.642	-0.152	0.116	0.910
CFAD	CFD	Complement factor D	LVEF	0.000	0.051	0.997	0.022	0.057	0.861	-0.111	0.113	0.910
CFAH	CFH	Complement factor H	LVEF	0.008	0.051	0.966	0.031	0.057	0.798	-0.076	0.114	0.910
CFAI	CFI	Complement factor I	LVEF	0.002	0.052	0.990	0.018	0.058	0.895	-0.057	0.120	0.928
CHLE	BCHE	Cholinesterase	LVEF	0.129	0.052	0.072	**0.152**	**0.057**	**0.048**	-0.040	0.119	0.928
CLUS	CLU	Clusterin	LVEF	0.141	0.052	0.053	**0.180**	**0.058**	**0.016**	-0.082	0.116	0.910
CMGA	CHGA	Chromogranin-A	LVEF	-0.002	0.119	0.996	-0.067	0.123	0.798	n < 50		
CNDP1	CNDP1	Beta-Ala-His dipeptidase	LVEF	**0.188**	**0.051**	**0.004**	**0.221**	**0.057**	**0.002**	-0.001	0.114	0.994
CO1A1	COL1A1	Collagen alpha-1	LVEF	-0.063	0.110	0.815	-0.065	0.122	0.803	n < 50		
CO2	C2	Complement C2	LVEF	-0.120	0.053	0.113	-0.139	0.059	0.077	0.014	0.122	0.985
CO3	C3	Complement C3	LVEF	-0.018	0.050	0.915	-0.015	0.055	0.895	-0.027	0.120	0.956
CO4A	C4A	Complement C4-A	LVEF	0.034	0.052	0.773	0.061	0.059	0.550	-0.032	0.117	0.945
CO4B	C4B	Complement C4-B	LVEF	0.031	0.053	0.815	0.069	0.059	0.497	-0.145	0.117	0.910
CO5	C5	Complement C5	LVEF	-0.069	0.052	0.413	-0.114	0.058	0.156	0.133	0.116	0.910
CO6	C6	Complement component C6	LVEF	0.050	0.052	0.583	0.024	0.059	0.859	0.201	0.117	0.910
CO7	C7	Complement component C7	LVEF	**-0.191**	**0.050**	**0.004**	**-0.249**	**0.056**	**0.001**	0.067	0.112	0.925
CO8A	C8A	Complement component C8 alpha chain	LVEF	0.016	0.053	0.928	0.022	0.059	0.876	0.046	0.114	0.928
CO8B	C8B	Complement component C8 beta chain	LVEF	0.013	0.053	0.939	0.043	0.059	0.691	-0.053	0.114	0.928
CO8G	C8G	Complement component C8 gamma chain	LVEF	-0.006	0.053	0.975	0.029	0.059	0.809	-0.096	0.118	0.910
CO9	C9	Complement component C9 [C9a; C9b]	LVEF	-0.115	0.052	0.120	-0.138	0.057	0.074	-0.784	0.451	0.910
			LVEF'							0.169	0.147	
			LVEF''							-0.297	0.418	
COF1	CFL1	Cofilin-1	LVEF	-0.140	0.105	0.415	-0.154	0.118	0.423	n < 50		
COL11	COLEC11	Collectin-11	LVEF	**-0.209**	**0.057**	**0.005**	**-0.261**	**0.063**	**0.001**	-0.004	0.143	0.994
COMP	COMP	Cartilage oligomeric matrix protein	LVEF	-0.036	0.052	0.754	0.018	0.059	0.895	-0.265	0.119	0.910
CPN2	CPN2	Carboxypeptidase N subunit 2	LVEF	0.125	0.052	0.083	0.501	0.232	0.073	0.090	0.121	0.910
			LVEF'				-0.062	0.066				
			LVEF''				0.100	0.231				
CRAC1	CRTAC1	Cartilage acidic protein 1	LVEF	0.058	0.100	0.815	0.030	0.118	0.895	n < 50		
CRIS3	CRISP3	Cysteine-rich secretory protein 3	LVEF	-0.053	0.053	0.566	-0.094	0.059	0.285	0.146	0.119	0.910
CRP	CRP	C-reactive protein [Cleaved into: C-reactive protein	LVEF	-0.097	0.056	0.254	-0.105	0.061	0.240	-0.047	0.134	0.928
CXCL7	PPBP	Platelet basic protein	LVEF	-0.044	0.053	0.671	-0.041	0.059	0.705	-0.050	0.120	0.928
CYTC	CST3	Cystatin-C	LVEF	-0.034	0.059	0.815	-0.073	0.067	0.540	0.198	0.126	0.910
DCD	DCD	Dermcidin	LVEF	0.693	0.319	0.054	0.749	0.328	0.073	n < 50		
			LVEF'	-0.262	0.088		-0.293	0.095				
			LVEF''	0.903	0.304		1.075	0.337				
DEF3	DEFA3	Neutrophil defensin 3	LVEF	0.015	0.055	0.937	0.003	0.061	0.984	0.075	0.129	0.925
DESP	DSP	Desmoplakin	LVEF	0.079	0.055	0.370	0.066	0.062	0.543	0.152	0.125	0.910
DIAC	CTBS	Di-N-acetylchitobiase	LVEF	-0.108	0.102	0.547	-0.112	0.114	0.586	n < 50		
DOPO	DBH	Dopamine beta-hydroxylase	LVEF	0.022	0.058	0.904	0.034	0.065	0.807	-0.058	0.125	0.928
DPP4	DPP4	Dipeptidyl peptidase 4	LVEF	0.140	0.149	0.597	0.389	0.168	0.093	n < 50		
ECM1	ECM1	Extracellular matrix protein 1	LVEF	-0.121	0.053	0.104	**-0.165**	**0.058**	**0.028**	0.115	0.121	0.910
EMAL6	EML6	Echinoderm microtubule-associated protein-like 6	LVEF	0.102	0.062	0.281	0.091	0.070	0.423	0.123	0.128	0.910
ENPP2	ENPP2	Ectonucleotide pyrophosphatase/phosphodiesterase family member 2	LVEF	-0.084	0.125	0.773	n < 50			n < 50		
EPCR	PROCR	Endothelial protein C receptor	LVEF	0.057	0.095	0.815	0.027	0.106	0.895	n < 50		
F13A	F13A1	Coagulation factor XIII A chain	LVEF	**0.226**	**0.052**	**0.001**	**0.242**	**0.057**	**0.001**	0.137	0.121	0.910
F13B	F13B	Coagulation factor XIII B chain	LVEF	**0.235**	**0.052**	**0.001**	**0.264**	**0.057**	**<0.001**	0.083	0.121	0.910
FA10	F10	Coagulation factor X	LVEF	0.032	0.052	0.805	0.044	0.058	0.676	-0.070	0.118	0.925
FA11	F11	Coagulation factor XI	LVEF	**0.176**	**0.052**	**0.010**	**0.200**	**0.058**	**0.007**	0.001	0.122	0.994
FA12	F12	Coagulation factor XII	LVEF	0.137	0.052	0.058	0.144	0.058	0.073	0.080	0.119	0.910
FA5	F5	Coagulation factor V	LVEF	0.104	0.052	0.188	0.089	0.058	0.310	0.181	0.120	0.910
FA7	F7	Coagulation factor VII	LVEF	0.030	0.072	0.885	-0.010	0.082	0.946	n < 50		
FA9	F9	Coagulation factor IX	LVEF	0.058	0.052	0.519	0.054	0.058	0.606	0.044	0.122	0.928
FBLN1	FBLN1	Fibulin-1	LVEF	**-0.181**	**0.051**	**0.006**	**-0.197**	**0.056**	**0.006**	-0.074	0.116	0.925
FBLN3	EFEMP1	EGF-containing fibulin-like extracellular matrix protein 1	LVEF	**-0.186**	**0.048**	**0.003**	**-0.226**	**0.054**	**0.001**	0.035	0.104	0.928
FBN1	FBN1	Fibrillin-1 [Cleaved into: Asprosin]	LVEF	-0.161	0.092	0.252	-0.167	0.099	0.245	n < 50		
FCG3A	FCGR3A	Low affinity immunoglobulin gamma Fc region receptor III-A	LVEF	-0.231	0.085	0.053	-0.232	0.095	0.074	n < 50		
FCGBP	FCGBP	IgGFc-binding protein	LVEF	-0.063	0.052	0.467	-0.070	0.058	0.474	-0.066	0.120	0.925
FCN3	FCN3	Ficolin-3	LVEF	**0.298**	**0.051**	**<0.001**	**0.326**	**0.056**	**<0.001**	0.103	0.121	0.910
FETUA	AHSG	Alpha-2-HS-glycoprotein	LVEF	0.042	0.053	0.691	0.008	0.059	0.944	0.176	0.118	0.910
FETUB	FETUB	Fetuin-B	LVEF	0.086	0.051	0.265	0.090	0.058	0.295	0.063	0.115	0.925
FGL1	FGL1	Fibrinogen-like protein 1	LVEF	-0.171	0.109	0.311	-0.259	0.122	0.128	n < 50		
FHR1	CFHR1	Complement factor H-related protein 1	LVEF	**0.152**	**0.052**	**0.028**	**0.159**	**0.058**	**0.040**	0.110	0.122	0.910
FHR2	CFHR2	Complement factor H-related protein 2	LVEF	-0.008	0.213	0.230	-0.045	0.226	0.083	0.117	0.121	0.910
			LVEF'	0.059	0.058		0.079	0.065				
			LVEF''	-0.299	0.197		-0.404	0.225				
FHR3	CFHR3	Complement factor H-related protein 3	LVEF	0.151	0.123	0.457	n < 50			n < 50		
FHR5	CFHR5	Complement factor H-related protein 5	LVEF	**0.188**	**0.052**	**0.006**	**0.222**	**0.058**	**0.002**	0.015	0.121	0.985
FIBA	FGA	Fibrinogen alpha chain	LVEF	-0.051	0.052	0.574	-0.076	0.058	0.421	0.096	0.114	0.910
FIBB	FGB	Fibrinogen beta chain	LVEF	-0.006	0.052	0.975	-0.040	0.058	0.705	0.184	0.114	0.910
FIBG	FGG	Fibrinogen gamma chain	LVEF	-0.055	0.051	0.541	-0.082	0.057	0.356	0.101	0.113	0.910
FINC	FN1	Fibronectin	LVEF	-0.032	0.053	0.805	-0.041	0.059	0.705	0.056	0.116	0.928
FRIL	FTL	Ferritin light chain	LVEF	-0.065	0.116	0.818	-0.114	0.120	0.596	n < 50		
G3P	GAPDH	Glyceraldehyde-3-phosphate dehydrogenase	LVEF	-0.114	0.067	0.261	-0.140	0.073	0.170	n < 50		
GANAB	GANAB	Neutral alpha-glucosidase AB	LVEF	-0.002	0.086	0.993	-0.023	0.095	0.895	n < 50		
GELS	GSN	Gelsolin	LVEF	**0.158**	**0.052**	**0.023**	**0.198**	**0.058**	**0.007**	0.007	0.123	0.991
GGH	GGH	Gamma-glutamyl hydrolase	LVEF	-0.007	0.066	0.975	-0.002	0.074	0.988	n < 50		
GP1BA	GP1BA	Platelet glycoprotein Ib alpha chain	LVEF	-0.652	0.226	0.136	-0.701	0.241	0.112	0.233	0.134	0.910
			LVEF'	0.159	0.061		0.164	0.069				
			LVEF''	-0.467	0.210		-0.475	0.241				
GPX3	GPX3	Glutathione peroxidase 3	LVEF	-0.055	0.053	0.547	-0.049	0.058	0.642	-0.094	0.118	0.910
HABP2	HABP2	Hyaluronan-binding protein 2	LVEF	-0.010	0.052	0.949	-0.030	0.058	0.807	0.071	0.120	0.925
HAT1	HAT1	Histone acetyltransferase type B catalytic subunit	LVEF	0.022	0.085	0.939	-0.004	0.094	0.984	n < 50		
HBA	HBA1; HBA2	Hemoglobin subunit alpha	LVEF	0.036	0.058	0.801	0.028	0.063	0.843	0.051	0.137	0.928

HBB	HBB	Hemoglobin subunit beta	LVEF	0.002	0.055	0.990	-0.001	0.060	0.988	0.006	0.134	0.991
HBG1	HBG1	Hemoglobin subunit gamma-1	LVEF	-0.022	0.098	0.939	-0.104	0.108	0.589	n < 50		
HEMO	HPX	Hemopexin	LVEF	0.108	0.052	0.160	0.077	0.057	0.401	0.202	0.118	0.910
HEP2	SERPIND1	Heparin cofactor 2	LVEF	0.106	0.052	0.165	0.137	0.057	0.075	-0.053	0.118	0.928
HGFA	HGFAC	Hepatocyte growth factor activator	LVEF	0.087	0.053	0.285	0.045	0.059	0.676	0.307	0.112	0.550
HGFL	MST1	Hepatocyte growth factor-like protein	LVEF	0.028	0.053	0.828	0.025	0.059	0.854	0.061	0.121	0.928
HPT	HP	Haptoglobin	LVEF	-0.011	0.053	0.939	0.641	0.233	0.170	-0.162	0.120	0.910
			LVEF'				-0.163	0.067				
			LVEF''				0.505	0.231				
HPTR	HPR	Haptoglobin-related protein	LVEF	**0.718**	**0.216**	**0.026**	**0.184**	**0.058**	**0.015**	-0.072	0.123	0.925
			LVEF'	**-0.146**	**0.059**							
			LVEF''	**0.433**	**0.201**							
HRG	HRG	Histidine-rich glycoprotein	LVEF	0.003	0.053	0.990	-0.016	0.059	0.895	0.094	0.120	0.910
HV118	IGHV1-18	Immunoglobulin heavy variable 1-18	LVEF	-0.070	0.086	0.691	-0.123	0.096	0.433	n < 50		
HV146	IGHV1-46	Immunoglobulin heavy variable 1-46	LVEF	0.033	0.058	0.818	0.013	0.066	0.919	0.114	0.122	0.910
HV307	IGHV3-7	Immunoglobulin heavy variable 3-7	LVEF	0.050	0.105	0.855	0.057	0.115	0.809	n < 50		
HV372	IGHV3-72	Immunoglobulin heavy variable 3-72	LVEF	-0.057	0.072	0.694	-0.071	0.079	0.610	n < 50		
HV374	IGHV3-74	Immunoglobulin heavy variable 3-74	LVEF	0.044	0.060	0.739	0.014	0.066	0.918	n < 50		
HV551	IGHV5-51	Immunoglobulin heavy variable 5-51	LVEF	0.041	0.129	0.926	0.127	0.144	0.621	n < 50		
HVC05	IGHV3-30-5	Immunoglobulin heavy variable 3-30-5	LVEF	-0.030	0.118	0.939	-0.064	0.128	0.809	n < 50		
HYDIN	HYDIN	Hydrocephalus-inducing protein homolog	LVEF	-0.019	0.053	0.915	-0.030	0.059	0.807	0.052	0.121	0.928
IBP2	IGFBP2	Insulin-like growth factor-binding protein 2	LVEF	-0.086	0.065	0.413	-0.137	0.074	0.188	n < 50		
IBP3	IGFBP3	Insulin-like growth factor-binding protein 3	LVEF	0.086	0.048	0.240	0.100	0.054	0.185	0.011	0.104	0.988
IBP4	IGFBP4	Insulin-like growth factor-binding protein 4	LVEF	-0.062	0.055	0.505	-0.049	0.060	0.642	-0.159	0.126	0.910
IBP6	IGFBP6	Insulin-like growth factor-binding protein 6	LVEF	0.078	0.070	0.519	0.081	0.076	0.543	n < 50		
IC1	SERPING1	Plasma protease C1 inhibitor	LVEF	-0.004	0.053	0.981	-0.012	0.059	0.919	0.059	0.122	0.928
ICAM2	ICAM2	Intercellular adhesion molecule 2	LVEF	1.324	0.563	0.311	n < 50			n < 50		
			LVEF'	-0.280	0.151							
			LVEF''	0.758	0.500							
IGA2	IGA2*	Immunoglobulin alpha-2 heavy chain	LVEF	-0.018	0.053	0.915	-0.043	0.059	0.688	0.127	0.116	0.910
IGD	IGD*	Immunoglobulin delta heavy chain	LVEF	-0.055	0.103	0.828	-0.094	0.114	0.642	n < 50		
IGF1	IGF1	Insulin-like growth factor I	LVEF	-0.310	0.235	0.192	0.064	0.065	0.585	-0.661	0.421	0.910
			LVEF'	0.058	0.064					0.128	0.145	
			LVEF''	-0.088	0.221					-0.124	0.418	
IGF2	IGF2	Insulin-like growth factor II	LVEF	-0.040	0.089	0.871	-0.056	0.100	0.796	n < 50		
IGG1	IGG1	Immunoglobulin gamma-1 heavy chain	LVEF	-0.048	0.053	0.608	-0.091	0.059	0.297	0.116	0.116	0.910
IGHA1	IGHA1	Immunoglobulin heavy constant alpha 1	LVEF	-0.004	0.053	0.981	-0.017	0.059	0.895	0.017	0.122	0.983
IGHG2	IGHG2	Immunoglobulin heavy constant gamma 2	LVEF	-0.040	0.053	0.721	-0.054	0.059	0.606	-0.709	0.460	0.910
			LVEF'							0.356	0.150	
			LVEF''							-1.100	0.426	
IGHG3	IGHG3	Immunoglobulin heavy constant gamma 3	LVEF	0.028	0.053	0.828	-0.003	0.059	0.984	0.195	0.120	0.910
IGHG4	IGHG4	Immunoglobulin heavy constant gamma 4	LVEF	-0.032	0.058	0.821	-0.063	0.063	0.583	0.101	0.138	0.910
IGHM	IGHM	Immunoglobulin heavy constant mu	LVEF	0.067	0.053	0.427	0.051	0.059	0.621	0.125	0.119	0.910
IGJ	JCHAIN	Immunoglobulin J chain	LVEF	0.001	0.061	0.996	0.002	0.068	0.988	-0.006	0.144	0.991
IGK	IGK*	Immunoglobulin kappa light chain	LVEF	-0.010	0.053	0.948	-0.053	0.059	0.609	0.162	0.114	0.910
IGKC	IGKC	Immunoglobulin kappa constant	LVEF	-0.013	0.053	0.939	-0.016	0.059	0.895	0.009	0.123	0.991
IGL1	IGL1*	Immunoglobulin lambda-1 light chain	LVEF	0.057	0.054	0.547	0.029	0.060	0.809	0.166	0.123	0.910
IGLC3	IGLC3	Immunoglobulin lambda constant 3	LVEF	0.003	0.053	0.990	-0.021	0.059	0.878	0.087	0.120	0.910
IL1AP	IL1RAP	Interleukin-1 receptor accessory protein	LVEF	-0.082	0.083	0.574	-0.084	0.092	0.606	n < 50		
ILK	ILK	Integrin-linked protein kinase	LVEF	-0.001	0.102	0.996	-0.072	0.114	0.747	n < 50		
INHBC	INHBC	Inhibin beta C chain	LVEF	0.146	0.077	0.210	0.199	0.083	0.077	n < 50		
IPSP	SERPINA5	Plasma serine protease inhibitor	LVEF	**0.165**	**0.052**	**0.016**	**0.175**	**0.057**	**0.016**	0.097	0.121	0.910
ITB1	ITGB1	Integrin beta-1	LVEF	-0.119	0.161	0.729	n < 50			n < 50		
ITIH1	ITIH1	Inter-alpha-trypsin inhibitor heavy chain H1	LVEF	**0.439**	**0.211**	**0.019**	**0.376**	**0.224**	**0.007**	0.051	0.121	0.928
			LVEF'	-0.031	0.057		0.007	0.064				
			LVEF''	-0.013	0.195		-0.179	0.223				
ITIH2	ITIH2	Inter-alpha-trypsin inhibitor heavy chain H2	LVEF	0.069	0.053	0.419	0.190	0.230	0.140	0.051	0.122	0.928
			LVEF'				0.025	0.066				
			LVEF''				-0.208	0.229				
ITIH3	ITIH3	Inter-alpha-trypsin inhibitor heavy chain H3	LVEF	**-0.217**	**0.050**	**0.001**	-0.276	0.055	<0.001	0.114	0.103	0.910
ITIH4	ITIH4	Inter-alpha-trypsin inhibitor heavy chain H4	LVEF	-0.101	0.053	0.203	-0.123	0.059	0.128	-0.017	0.121	0.983
K1C9	KRT9	Keratin, type I cytoskeletal 9	LVEF	-0.130	0.053	0.076	-0.120	0.058	0.134	-0.173	0.119	0.910
K2C1	KRT1	Keratin, type II cytoskeletal 1	LVEF	-0.122	0.053	0.104	-0.112	0.058	0.170	-0.155	0.120	0.910
KAIN	SERPINA4	Kallistatin	LVEF	0.087	0.053	0.283	0.120	0.059	0.136	-0.078	0.120	0.919
KLKB1	KLKB1	Plasma kallikrein	LVEF	**0.191**	**0.050**	**0.003**	**0.238**	**0.056**	**0.001**	-0.042	0.116	0.928
KNG1	KNG1	Kininogen-1	LVEF	**0.167**	**0.051**	**0.013**	**0.198**	**0.057**	**0.006**	-0.033	0.112	0.943
KV106	IGKV1-6	Immunoglobulin kappa variable 1-6	LVEF	0.148	0.141	0.547	n < 50			n < 50		
KV320	IGKV3-20	Immunoglobulin kappa variable 3-20	LVEF	0.013	0.060	0.939	-0.026	0.067	0.861	n < 50		
KV401	IGKV4-1	Immunoglobulin kappa variable 4-1	LVEF	-0.049	0.061	0.691	-0.098	0.067	0.340	0.233	0.136	0.910
KVD11	IGKV3D-11	Immunoglobulin kappa variable 3D-11	LVEF	-0.555	0.531	0.279	n < 50			n < 50		
			LVEF'	0.267	0.141							
			LVEF''	-1.079	0.487							
KVD15	IGKV3D-15	Immunoglobulin kappa variable 3D-15	LVEF	-0.086	0.074	0.487	-0.120	0.081	0.339	n < 50		
LBP	LBP	Lipopolysaccharide-binding protein	LVEF	**-0.154**	**0.052**	**0.027**	**-0.193**	**0.058**	**0.009**	0.023	0.120	0.962
LCAT	LCAT	Phosphatidylcholine-sterol acyltransferase	LVEF	**0.228**	**0.051**	**0.001**	**0.225**	**0.057**	**0.002**	0.283	0.117	0.834
LDHB	LDHB	L-lactate dehydrogenase B chain	LVEF	-0.095	0.069	0.393	-0.125	0.077	0.277	n < 50		
LG3BP	LGALS3BP	Galectin-3-binding protein	LVEF	-0.102	0.052	0.189	-0.118	0.058	0.135	-0.022	0.119	0.962
LRP1	LRP1	Prolow-density lipoprotein receptor-related protein 1	LVEF	0.020	0.082	0.939	0.047	0.091	0.807	n < 50		
LUM	LUM	Lumican	LVEF	-0.137	0.052	0.055	**-0.154**	**0.058**	**0.048**	-0.047	0.121	0.928
LV147	IGLV1-47	Immunoglobulin lambda variable 1-47	LVEF	-0.097	0.085	0.505	-0.103	0.091	0.509	n < 50		
LV319	IGLV3-19	Immunoglobulin lambda variable 3-19	LVEF	0.022	0.075	0.928	-0.014	0.081	0.926	n < 50		
LV321	IGLV3-21	Immunoglobulin lambda variable 3-21	LVEF	0.015	0.061	0.939	-0.009	0.068	0.946	n < 50		
LV861	IGLV8-61	Immunoglobulin lambda variable 8-61	LVEF	0.077	0.133	0.815	n < 50			n < 50		
LYAM1	SELL	L-selectin	LVEF	0.014	0.053	0.939	-0.035	0.059	0.773	0.174	0.116	0.910
LYSC	LYZ	Lysozyme C	LVEF	-0.065	0.053	0.445	-0.125	0.058	0.117	0.143	0.116	0.910
LYVE1	LYVE1	Lymphatic vessel endothelial hyaluronic acid receptor 1	LVEF	-0.130	0.111	0.487	-0.134	0.127	0.548	n < 50		
MARCO	MARCO	Macrophage receptor MARCO	LVEF	-0.229	0.121	0.215	-0.246	0.128	0.170	n < 50		
MASP1	MASP1	Mannan-binding lectin serine protease 1	LVEF	0.006	0.053	0.975	0.049	0.059	0.642	-0.165	0.119	0.910

MASP2	MASP2	Mannan-binding lectin serine protease 2	LVEF	0.148	0.059	0.069	0.140	0.066	0.122	0.142	0.129	0.910
MAST4	MAST4	Microtubule-associated serine/threonine-protein kinase 4	LVEF	0.044	0.139	0.926	n < 50			n < 50		
MBL2	MBL2	Mannose-binding protein C	LVEF	-0.017	0.063	0.939	-0.045	0.072	0.747	0.050	0.130	0.928
MMP2	MMP2	72 kDa type IV collagenase	LVEF	**-0.294**	**0.086**	**0.010**	**-0.292**	**0.093**	**0.015**	n < 50		
MMRN2	MMRN2	Multimerin-2	LVEF	0.096	0.166	0.815	0.029	0.169	0.926	n < 50		
MTBP	MTBP	Mdm2-binding protein	LVEF	**0.341**	**0.105**	**0.018**	**0.344**	**0.115**	**0.025**	n < 50		
MUC18	MCAM	Cell surface glycoprotein MUC18	LVEF	**-0.201**	**0.064**	**0.018**	**-0.217**	**0.070**	**0.016**	n < 50		
MUC19	MUC19	Mucin-19	LVEF	0.056	0.054	0.549	0.040	0.060	0.730	0.120	0.122	0.910
MYH4	MYH4	Myosin-4	LVEF	-0.015	0.079	0.949	-0.032	0.086	0.876	n < 50		
NCAM1	NCAM1	Neural cell adhesion molecule 1	LVEF	-0.015	0.071	0.944	0.001	0.077	0.994	n < 50		
NCHL1	CHL1	Neural cell adhesion molecule L1-like protein	LVEF	1.067	0.389	0.213	0.058	0.106	0.796	n < 50		
			LVEF'	-0.266	0.102							
			LVEF''	0.819	0.337							
NOE1	OLFM1	Noelin	LVEF	-0.153	0.117	0.419	-0.170	0.124	0.395	n < 50		
NOTC3	NOTCH3	Neurogenic locus notch homolog protein 3	LVEF	-0.010	0.098	0.975	-0.033	0.105	0.895	n < 50		
NRP1	NRP1	Neuropilin-1	LVEF	0.059	0.107	0.822	-0.005	0.122	0.984	n < 50		
OR3A2	OR3A2	Olfactory receptor 3A2	LVEF	0.048	0.054	0.626	-0.014	0.060	0.900	0.330	0.116	0.550
P4HA3	P4HA3	Prolyl 4-hydroxylase subunit alpha-3	LVEF	0.190	0.076	0.073	**0.243**	**0.082**	**0.023**	n < 50		
PAFA	PLA2G7	Platelet-activating factor acetylhydrolase	LVEF	0.027	0.088	0.928	-0.018	0.093	0.919	n < 50		
PARP9	PARP9	Protein mono-ADP-ribosyltransferase PARP9	LVEF	-0.184	0.309	0.072	**-0.208**	**0.333**	**0.007**	n < 50		
			LVEF'	-0.064	0.084		**-0.102**	**0.094**				
			LVEF''	0.383	0.284		**0.597**	**0.321**				
PCOC1	PCOLCE	Procollagen C-endopeptidase enhancer 1	LVEF	-0.001	0.056	0.996	0.018	0.063	0.895	-0.086	0.126	0.910
PCYOX	PCYOX1	Prenylcysteine oxidase 1	LVEF	0.481	0.216	0.110	0.120	0.058	0.134	-0.106	0.122	0.910
			LVEF'	-0.066	0.058							
			LVEF''	0.117	0.200							
PEDF	SERPINF1	Pigment epithelium-derived factor	LVEF	**0.250**	**0.050**	**<0.001**	**0.236**	**0.055**	**0.001**	0.278	0.117	0.834
PEPD	PEPD	Xaa-Pro dipeptidase	LVEF	0.139	0.147	0.597	n < 50			n < 50		
PGBM	HSPG2	Basement membrane-specific heparan sulfate proteoglycan core protein	LVEF	-0.085	0.056	0.329	-0.074	0.062	0.476	-0.127	0.140	0.910
PGRP2	PGLYRP2	N-acetylmuramoyl-L-alanine amidase	LVEF	0.033	0.053	0.801	-0.020	0.058	0.884	0.244	0.117	0.910
PHLD	GPLD1	Phosphatidylinositol-glycan-specific phospholipase D	LVEF	0.109	0.052	0.158	0.132	0.059	0.095	-0.065	0.108	0.925
PI16	PI16	Peptidase inhibitor 16	LVEF	0.070	0.056	0.440	0.115	0.062	0.185	-0.111	0.126	0.910
PLAK	JUP	Junction plakoglobin	LVEF	0.003	0.082	0.990	0.024	0.090	0.895	n < 50		
PLF4	PF4	Platelet factor 4	LVEF	-0.024	0.054	0.873	-0.010	0.060	0.926	-0.071	0.126	0.925
PLMN	PLG	Plasminogen	LVEF	0.137	0.051	0.054	**0.171**	**0.058**	**0.023**	-0.029	0.118	0.950
PLSL	LCP1	Plastin-2	LVEF	-0.094	0.060	0.311	-0.079	0.067	0.486	-0.122	0.131	0.910
PLTP	PLTP	Phospholipid transfer protein	LVEF	-0.119	0.053	0.113	-0.137	0.058	0.077	0.007	0.122	0.991
PON1	PON1	Serum paraoxonase/arylesterase 1	LVEF	**0.194**	**0.051**	**0.004**	**0.217**	**0.057**	**0.003**	0.044	0.115	0.928
PON3	PON3	Serum paraoxonase/lactonase 3	LVEF	0.085	0.066	0.422	0.080	0.074	0.541	n < 50		
PRDX2	PRDX2	Peroxiredoxin-2	LVEF	0.021	0.085	0.939	-0.008	0.091	0.975	n < 50		
PRG2	PRG2	Bone marrow proteoglycan	LVEF	-0.048	0.110	0.873	0.007	0.119	0.984	n < 50		
PRG4	PRG4	Proteoglycan 4	LVEF	**0.526**	**0.208**	**0.003**	**0.623**	**0.221**	**0.002**	0.170	0.117	0.910
			LVEF'	**-0.047**	**0.056**		**-0.079**	**0.063**				
			LVEF''	**0.062**	**0.192**		**0.170**	**0.219**				
PROC	PROC	Vitamin K-dependent protein C	LVEF	0.105	0.052	0.183	0.139	0.058	0.074	-0.117	0.120	0.910
PROF1	PFN1	Profilin-1	LVEF	-0.011	0.084	0.975	0.026	0.093	0.895	n < 50		
PROP	CFP	Properdin	LVEF	**0.169**	**0.052**	**0.015**	**0.186**	**0.057**	**0.012**	0.066	0.120	0.925
PROS	PROS1	Vitamin K-dependent protein S	LVEF	0.015	0.053	0.928	0.028	0.059	0.811	-0.013	0.113	0.985
PROZ	PROZ	Vitamin K-dependent protein Z	LVEF	0.019	0.056	0.925	0.018	0.062	0.895	-0.044	0.126	0.928
PSB1	PSMB1	Proteasome subunit beta type-1	LVEF	-0.009	0.089	0.975	-0.025	0.100	0.895	n < 50		
PSPB	SFTPB	Pulmonary surfactant-associated protein B	LVEF	-0.029	0.162	0.951	n < 50			n < 50		
PTGDS	PTGDS	Prostaglandin-H2 D-isomerase	LVEF	0.029	0.068	0.880	0.020	0.073	0.895	n < 50		
PTMA	PTMA	Prothymosin alpha [Cleaved into: Prothymosin alpha, N-terminally processed; Thymosin alpha-1]	LVEF	-0.102	0.067	0.325	-0.079	0.074	0.543	n < 50		
PVR	PVR	Poliovirus receptor	LVEF	0.006	0.153	0.990	**n < 50**			n < 50		
PXDC2	PLXDC2	Plexin domain-containing protein 2	LVEF	-0.116	0.088	0.415	-0.121	0.094	0.427	n < 50		
PZP	PZP	Pregnancy zone protein	LVEF	-0.025	0.050	0.842	-0.017	0.067	0.895	-0.109	0.122	0.910
QSOX1	QSOX1	Sulfhydryl oxidase 1	LVEF	**-0.150**	**0.051**	**0.026**	**-0.225**	**0.058**	**0.002**	0.160	0.119	0.910
RADX	RADX	RPA-related protein RADX	LVEF	-0.138	0.141	0.581	**n < 50**			n < 50		
RARR2	RARRES2	Retinoic acid receptor responder protein 2	LVEF	-0.006	0.053	0.975	-0.028	0.059	0.813	0.077	0.119	0.919
RET4	RBP4	Retinol-binding protein 4	LVEF	0.134	0.053	0.067	**0.181**	**0.058**	**0.016**	-0.154	0.117	0.910
RN112	RNF112	RING finger protein 112	LVEF	0.064	0.056	0.505	0.084	0.062	0.396	-0.050	0.125	0.928
RNAS1	RNASE1	Ribonuclease pancreatic	LVEF	**-0.181**	**0.049**	**0.005**	**-0.216**	**0.054**	**0.002**	-0.001	0.118	0.994
RNAS4	RNASE4	Ribonuclease 4	LVEF	0.014	0.163	0.978	n < 50			n < 50		
S10A9	S100A9	Protein S100-A9	LVEF	-0.103	0.080	0.422	-0.079	0.088	0.610	n < 50		
SAA1	SAA1	Serum amyloid A-1 protein	LVEF	-0.084	0.053	0.303	-0.077	0.059	0.421	-0.099	0.121	0.910
SAA2	SAA2	Serum amyloid A-2 protein	LVEF	-0.094	0.053	0.246	-0.087	0.059	0.335	-0.115	0.124	0.910
SAA4	SAA4	Serum amyloid A-4 protein	LVEF	0.072	0.053	0.393	0.071	0.059	0.476	-0.932	0.456	0.910
			LVEF'							0.380	0.148	
			LVEF''							-1.053	0.422	
SAMP	APCS	Serum amyloid P-component	LVEF	**0.198**	**0.051**	**0.003**	**0.241**	**0.056**	**0.001**	-0.015	0.121	0.985
SEPP1	SELENOP	Selenoprotein P	LVEF	0.035	0.053	0.773	0.099	0.059	0.244	0.966	0.436	0.550
			LVEF'							-0.400	0.142	
			LVEF''							1.041	0.404	
1433S	SFN	14-3-3 protein sigma	LVEF	-0.043	0.092	0.855	-0.195	0.408	0.157	n < 50		
			LVEF'				0.127	0.118				
			LVEF''				-0.646	0.412				
SHBG	SHBG	Sex hormone-binding globulin	LVEF	-0.081	0.049	0.281	-0.107	0.056	0.170	-0.031	0.117	0.948
SODE	SOD3	Extracellular superoxide dismutase [Cu-Zn]	LVEF	0.089	0.109	0.683	0.135	0.120	0.517	n < 50		
SPB3	SERPINB3	Serpin B3	LVEF	-0.035	0.098	0.915	-0.106	0.106	0.579	n < 50		
SPRL1	SPARCL1	SPARC-like protein 1	LVEF	-0.051	0.075	0.771	-0.116	0.085	0.395	n < 50		
TAGL2	TAGLN2	Transgelin-2	LVEF	-0.153	0.092	0.281	-0.163	0.102	0.285	n < 50		
TENA	TNC	Tenascin	LVEF	-0.077	0.133	0.815	0.023	0.140	0.926	n < 50		
TENX	TNXB	Tenascin-X	LVEF	0.031	0.080	0.901	0.067	0.090	0.687	n < 50		
TETN	CLEC3B	Tetranectin	LVEF	0.096	0.053	0.238	0.116	0.059	0.155	0.005	0.122	0.991
THBG	SERPINA7	Thyroxine-binding globulin	LVEF	-0.024	0.053	0.871	-0.062	0.059	0.545	0.049	0.116	0.928
THRB	F2	Prothrombin	LVEF	0.016	0.052	0.926	0.001	0.058	0.988	0.100	0.121	0.910

TIMP1	TIMP1	Metalloproteinase inhibitor 1	LVEF	-0.279	0.150	0.230	n < 50			n < 50		
TLN1	TLN1	Talin-1	LVEF	-0.124	0.090	0.393	-0.076	0.104	0.688	n < 50		
TRFE	TF	Serotransferrin	LVEF	-0.129	0.052	0.073	-0.142	0.058	0.073	-0.034	0.122	0.944
TSP1	THBS1	Thrombospondin-1	LVEF	-0.030	0.055	0.828	-0.056	0.061	0.606	0.086	0.124	0.910
TSP4	THBS4	Thrombospondin-4	LVEF	-0.022	0.061	0.915	-0.049	0.067	0.688	0.167	0.145	0.910
TTHY	TTR	Transthyretin	LVEF	**0.173**	**0.051**	**0.010**	**0.201**	**0.057**	**0.006**	-0.020	0.104	0.962
URP2	FERMT3	Fermitin family homolog 3	LVEF	-0.136	0.103	0.415	-0.138	0.116	0.486	n < 50		
VASN	VASN	Vasorin	LVEF	-0.533	0.225	0.252	-0.599	0.240	0.207	0.107	0.130	0.910
			LVEF'	0.149	0.060		0.163	0.068				
			LVEF''	-0.469	0.206		-0.499	0.236				
VASP	VASP	Vasodilator-stimulated phosphoprotein	LVEF	-0.106	0.082	0.422	-0.054	0.091	0.773	n < 50		
VCAM1	VCAM1	Vascular cell adhesion protein 1	LVEF	-0.108	0.062	0.252	-0.173	0.067	0.060	n < 50		
VINC	VCL	Vinculin	LVEF	-0.014	0.154	0.978	n < 50			n < 50		
VNN1	VNN1	Pantetheinase	LVEF	-0.057	0.054	0.547	-0.067	0.061	0.530	-0.040	0.123	0.929
VTDB	GC	Vitamin D-binding protein	LVEF	0.052	0.050	0.547	0.098	0.057	0.235	-0.151	0.107	0.910
VTNC	VTN	Vitronectin	LVEF	0.024	0.048	0.841	0.030	0.054	0.796	-0.039	0.103	0.928
VWF	VWF	von Willebrand factor	LVEF	**-0.716**	**0.205**	**0.001**	**-0.621**	**0.220**	**<0.001**	0.160	0.112	0.910
			LVEF'	**0.152**	**0.056**		**0.136**	**0.063**				
			LVEF''	**-0.515**	**0.190**		**-0.514**	**0.219**				
1433Z	YWHAZ	14-3-3 protein zeta/delta	LVEF	-0.265	0.108	0.083	-0.312	0.126	0.074	n < 50		
ZA2G	AZGP1	Zinc-alpha-2-glycoprotein	LVEF	-0.473	0.216	0.073	-0.459	0.231	0.073	-0.007	0.123	0.991
			LVEF'	0.159	0.058		0.165	0.066				
			LVEF''	-0.513	0.201		-0.525	0.230				
ZPI	SERPINA10	Protein Z-dependent protease inhibitor	LVEF	0.016	0.053	0.928	0.003	0.059	0.984	0.056	0.121	0.928

Supplemental Table S3. Associations of left ventricular end diastolic diameter (LVEDD$^{\text{acc. to HENRY}}$) and the plasma proteins measured using LC-MS/MS (untargeted approach). Results from regression models adjusted for sex, age and BMI for the whole study population and from sex-specific models adjusted for age and BMI.
Proteins were log10-transformed. Estimates are presented for z-transformed outcome and exposure variables. In the case of less than 50 observations per protein, no results are given.
stderr, standard error; FDR, false discovery rate

Outcome			Exposure	All subjects			Men			Women		
				beta	stderr	FDR	beta	stderr	FDR	beta	stderr	FDR
Entry name	Gene name	Protein name										
A1AG1	ORM1	Alpha-1-acid glycoprotein 1	LVEDD acc. to HENRY	0.087	0.052	0.592	0.104	0.059	0.535	0.002	0.120	0.995
A1AG2	ORM2	Alpha-1-acid glycoprotein 2	LVEDD acc. to HENRY	0.114	0.052	0.553	0.157	0.058	0.302	-0.056	0.120	0.985
A1AT	SERPINA1	Alpha-1-antitrypsin	LVEDD acc. to HENRY	0.026	0.053	0.989	0.065	0.059	0.785	-0.127	0.119	0.985
A1BG	A1BG	Alpha-1B-glycoprotein	LVEDD acc. to HENRY	-0.067	0.052	0.771	-0.052	0.059	0.879	-0.179	0.118	0.985
A2AP	SERPINF2	Alpha-2-antiplasmin	LVEDD acc. to HENRY	0.106	0.052	0.553	0.149	0.058	0.387	-0.104	0.120	0.985
A2GL	LRG1	Leucine-rich alpha-2-glycoprotein	LVEDD acc. to HENRY	0.053	0.053	0.867	0.074	0.059	0.716	-0.048	0.120	0.986
A2MG	A2M	Alpha-2-macroglobulin	LVEDD acc. to HENRY	0.049	0.053	0.902	0.044	0.059	0.946	0.075	0.120	0.985
AACT	SERPINA3	Alpha-1-antichymotrypsin	LVEDD acc. to HENRY	0.076	0.053	0.655	0.069	0.059	0.769	0.112	0.120	0.985
ACTB	ACTB	Actin, cytoplasmic 1	LVEDD acc. to HENRY	0.030	0.053	0.956	0.013	0.059	0.966	0.102	0.120	0.985
ACTS	ACTA1	Actin, alpha skeletal muscle	LVEDD acc. to HENRY	0.020	0.053	0.989	0.005	0.059	0.967	0.088	0.120	0.985
ADIPO	ADIPOQ	Adiponectin	LVEDD acc. to HENRY	0.046	0.052	0.908	0.039	0.059	0.966	0.092	0.120	0.985
AFAM	AFM	Afamin	LVEDD acc. to HENRY	-0.042	0.053	0.920	-0.048	0.059	0.902	-0.014	0.120	0.986
ALBU	ALB	Albumin	LVEDD acc. to HENRY	-0.013	0.053	0.989	-0.012	0.059	0.966	-0.356	0.560	0.985
			LVEDD acc. to HENRY'							0.239	0.176	
			LVEDD acc. to HENRY''							-0.843	0.506	
ALDOA	ALDOA	Fructose-bisphosphate aldolase A	LVEDD acc. to HENRY	-0.042	0.104	0.989	-0.042	0.113	0.966	n < 50		
ALDOB	ALDOB	Fructose-bisphosphate aldolase B	LVEDD acc. to HENRY	-0.032	0.081	0.989	-0.041	0.087	0.966	n < 50		
ALS	IGFALS	Insulin-like growth factor-binding protein complex acid labile subunit	LVEDD acc. to HENRY	0.062	0.052	0.773	0.088	0.059	0.616	-0.063	0.120	0.985
AMBP	AMBP	Protein AMBP [Cleaved into: Alpha-1-microglobulin	LVEDD acc. to HENRY	-0.133	0.052	0.466	-0.175	0.058	0.240	0.055	0.120	0.985
AMPN	ANPEP	Aminopeptidase N	LVEDD acc. to HENRY	-0.005	0.073	0.989	-0.030	0.079	0.966	n < 50		
ANGT	AGT	Angiotensinogen	LVEDD acc. to HENRY	-0.029	0.050	0.956	-0.022	0.059	0.966	-0.057	0.120	0.985
ANKMT	ANTKMT	Adenine nucleotide translocase lysine N-methyltransferase	LVEDD acc. to HENRY	-0.086	0.069	0.773	-0.163	0.078	0.443	n < 50		
ANT3	SERPINC1	Antithrombin-III	LVEDD acc. to HENRY	0.122	0.053	0.532	0.141	0.058	0.401	0.038	0.120	0.986
ANXA2	ANXA2	Annexin A2	LVEDD acc. to HENRY	0.010	0.054	0.989	-0.002	0.061	0.981	0.062	0.123	0.985
AOC3	AOC3	Membrane primary amine oxidase	LVEDD acc. to HENRY	-0.028	0.093	0.989	-0.045	0.100	0.966	n < 50		
APMAP	APMAP	Adipocyte plasma membrane-associated protein	LVEDD acc. to HENRY	-0.156	0.073	0.553	-0.177	0.082	0.440	n < 50		
APOA	LPA	Apolipoprotein	LVEDD acc. to HENRY	0.069	0.053	0.771	0.060	0.060	0.841	0.107	0.120	0.985
APOA1	APOA1	Apolipoprotein A-I	LVEDD acc. to HENRY	-0.075	0.051	0.655	-0.080	0.059	0.674	-0.069	0.120	0.985
APOA2	APOA2	Apolipoprotein A-II	LVEDD acc. to HENRY	-0.074	0.052	0.672	-0.067	0.059	0.777	-0.107	0.120	0.985
APOA4	APOA4	Apolipoprotein A-IV	LVEDD acc. to HENRY	-0.022	0.053	0.989	-0.012	0.059	0.966	-0.065	0.120	0.985
APOB	APOB	Apolipoprotein B-100	LVEDD acc. to HENRY	0.140	0.052	0.466	0.171	0.058	0.240	0.000	0.120	0.999
APOC1	APOC1	Apolipoprotein C-I	LVEDD acc. to HENRY	-0.059	0.053	0.811	-0.031	0.059	0.966	-0.172	0.119	0.985
APOC2	APOC2	Apolipoprotein C-II	LVEDD acc. to HENRY	-0.158	0.256	0.553	-0.012	0.059	0.966	0.152	0.545	0.985
			LVEDD acc. to HENRY'	-0.034	0.088					-0.250	0.171	
			LVEDD acc. to HENRY''	0.179	0.235					0.889	0.493	
APOC3	APOC3	Apolipoprotein C-III	LVEDD acc. to HENRY	-0.018	0.053	0.989	-0.009	0.059	0.966	-0.065	0.120	0.985
APOC4	APOC4	Apolipoprotein C-IV	LVEDD acc. to HENRY	-0.008	0.053	0.989	0.023	0.059	0.966	-0.160	0.119	0.985
APOD	APOD	Apolipoprotein D	LVEDD acc. to HENRY	0.047	0.053	0.906	0.028	0.059	0.966	0.144	0.119	0.985
APOE	APOE	Apolipoprotein E	LVEDD acc. to HENRY	0.001	0.053	0.996	0.027	0.059	0.966	-0.105	0.120	0.985
APOF	APOF	Apolipoprotein F	LVEDD acc. to HENRY	0.056	0.052	0.841	0.047	0.059	0.902	0.096	0.120	0.985
APOH	APOH	Beta-2-glycoprotein 1	LVEDD acc. to HENRY	-0.052	0.053	0.867	-0.077	0.059	0.674	0.062	0.120	0.985
APOL1	APOL1	Apolipoprotein L1	LVEDD acc. to HENRY	0.017	0.051	0.989	0.038	0.059	0.966	-0.075	0.120	0.985
APOM	APOM	Apolipoprotein M	LVEDD acc. to HENRY	-0.615	0.253	0.553	0.024	0.059	0.966	-1.413	0.546	0.985
			LVEDD acc. to HENRY'	0.197	0.086					0.387	0.172	
			LVEDD acc. to HENRY''	-0.473	0.232					-0.937	0.494	
ATL4	ADAMTSL4	ADAMTS-like protein 4	LVEDD acc. to HENRY	-0.023	0.057	0.989	-0.025	0.064	0.966	-0.018	0.127	0.986
ATRN	ATRN	Attractin	LVEDD acc. to HENRY	-0.012	0.053	0.989	-0.016	0.059	0.966	0.010	0.120	0.986
ATS13	ADAMTS13	A disintegrin and metalloproteinase with thrombospondin motifs 13	LVEDD acc. to HENRY	-0.104	0.134	0.920	n < 50			n < 50		
B2MG	B2M	Beta-2-microglobulin [Cleaved into: Beta-2-microglobulin form pI 5.3]	LVEDD acc. to HENRY	-0.007	0.052	0.989	-0.001	0.059	0.988	-0.033	0.120	0.986
BASP1	BASP1	Brain acid soluble protein 1	LVEDD acc. to HENRY	-0.083	0.084	0.867	-0.079	0.098	0.902	n < 50		
BGH3	TGFBI	Transforming growth factor-beta-induced protein ig-h3	LVEDD acc. to HENRY	-0.051	0.053	0.872	-0.005	0.059	0.966	-0.217	0.118	0.985
BIP	HSPA5	Endoplasmic reticulum chaperone BiP	LVEDD acc. to HENRY	-0.147	0.091	0.597	-0.104	0.102	0.838	n < 50		
BTD	BTD	Biotinidase	LVEDD acc. to HENRY	0.010	0.053	0.989	0.016	0.059	0.966	-0.018	0.120	0.986
C163A	CD163	Scavenger receptor cysteine-rich type 1 protein M130	LVEDD acc. to HENRY	-0.026	0.061	0.989	-0.059	0.068	0.882	0.174	0.148	0.985
C1QA	C1QA	Complement C1q subcomponent subunit A	LVEDD acc. to HENRY	0.005	0.052	0.989	0.014	0.059	0.966	-0.028	0.120	0.986
C1QB	C1QB	Complement C1q subcomponent subunit B	LVEDD acc. to HENRY	-0.059	0.052	0.811	-0.032	0.059	0.966	-0.172	0.119	0.985
C1QC	C1QC	Complement C1q subcomponent subunit C	LVEDD acc. to HENRY	0.006	0.052	0.989	0.027	0.059	0.966	-0.097	0.120	0.985
C1R	C1R	Complement C1r subcomponent	LVEDD acc. to HENRY	0.038	0.053	0.931	0.039	0.059	0.966	0.036	0.120	0.986
C1RL	C1RL	Complement C1r subcomponent-like protein	LVEDD acc. to HENRY	0.090	0.052	0.592	0.113	0.058	0.489	-0.008	0.120	0.986
C1S	C1S	Complement C1s subcomponent	LVEDD acc. to HENRY	-0.002	0.052	0.989	-0.006	0.059	0.966	0.013	0.120	0.986
C4BPA	C4BPA	C4b-binding protein alpha chain	LVEDD acc. to HENRY	-0.082	0.053	0.621	0.552	0.273	0.418	0.044	0.120	0.986
			LVEDD acc. to HENRY'				-0.205	0.091				
			LVEDD acc. to HENRY''				0.536	0.261				
C4BPB	C4BPB	C4b-binding protein beta chain	LVEDD acc. to HENRY	-0.089	0.053	0.592	-0.093	0.059	0.564	-0.065	0.120	0.985
CADH5	CDH5	Cadherin-5	LVEDD acc. to HENRY	0.001	0.054	0.996	0.027	0.061	0.966	-0.136	0.122	0.985
CAH1	CA1	Carbonic anhydrase 1	LVEDD acc. to HENRY	0.049	0.054	0.902	0.030	0.060	0.966	0.139	0.125	0.985
CALD1	CALD1	Caldesmon	LVEDD acc. to HENRY	-0.083	0.091	0.902	-0.102	0.099	0.831	n < 50		
CAMP	CAMP	Cathelicidin antimicrobial peptide	LVEDD acc. to HENRY	0.034	0.055	0.955	0.047	0.061	0.923	-0.025	0.123	0.986
CATA	CAT	Catalase	LVEDD acc. to HENRY	-0.010	0.064	0.989	0.012	0.070	0.966	n < 50		
CAVN2	CAVIN2	Caveolae-associated protein 2	LVEDD acc. to HENRY	0.033	0.135	0.989	n < 50			n < 50		
CBG	SERPINA6	Corticosteroid-binding globulin	LVEDD acc. to HENRY	0.057	0.051	0.811	0.078	0.059	0.674	0.011	0.120	0.986
CBPB2	CPB2	Carboxypeptidase B2	LVEDD acc. to HENRY	0.102	0.053	0.553	0.127	0.058	0.440	-0.011	0.120	0.986
CBPN	CPN1	Carboxypeptidase N catalytic chain	LVEDD acc. to HENRY	0.006	0.051	0.989	-0.014	0.059	0.966	0.098	0.120	0.985
CCL14	CCL14	C-C motif chemokine 14	LVEDD acc. to HENRY	0.009	0.139	0.989	-0.017	0.151	0.966	n < 50		
CD14	CD14	Monocyte differentiation antigen CD14	LVEDD acc. to HENRY	-0.096	0.053	0.592	-0.106	0.059	0.535	-0.052	0.120	0.985
CD44	CD44	CD44 antigen	LVEDD acc. to HENRY	-0.056	0.062	0.902	-0.014	0.070	0.966	n < 50		
CD5L	CD5L	CD5 antigen-like	LVEDD acc. to HENRY	-0.086	0.053	0.592	-0.094	0.059	0.564	-0.060	0.120	0.985
CERU	CP	Ceruloplasmin	LVEDD acc. to HENRY	0.031	0.052	0.956	0.042	0.059	0.959	-0.007	0.120	0.990
CETP	CETP	Cholesteryl ester transfer protein	LVEDD acc. to HENRY	-0.194	0.099	0.553	-0.215	0.116	0.535	n < 50		
CFAB	CFB	Complement factor B	LVEDD acc. to HENRY	0.069	0.053	0.770	0.058	0.059	0.842	0.118	0.120	0.985

CFAD	CFD	Complement factor D	LVEDD acc. to HENRY	-0.036	0.053	0.933	-0.072	0.059	0.740	0.121	0.120	0.985
CFAH	CFH	Complement factor H	LVEDD acc. to HENRY	-0.012	0.053	0.989	-0.053	0.059	0.879	0.171	0.119	0.985
CFAI	CFI	Complement factor I	LVEDD acc. to HENRY	0.005	0.053	0.989	0.022	0.059	0.966	-0.068	0.120	0.985
CHLE	BCHE	Cholinesterase	LVEDD acc. to HENRY	0.015	0.053	0.989	0.004	0.059	0.972	0.082	0.120	0.985
CLUS	CLU	Clusterin	LVEDD acc. to HENRY	-0.033	0.053	0.955	-0.034	0.059	0.966	-0.025	0.120	0.986
CMGA	CHGA	Chromogranin-A	LVEDD acc. to HENRY	0.128	0.128	0.867	0.040	0.144	0.966	n < 50		
CNDP1	CNDP1	Beta-Ala-His dipeptidase	LVEDD acc. to HENRY	-0.015	0.053	0.989	0.015	0.059	0.966	-0.235	0.121	0.985
CO1A1	COL1A1	Collagen alpha-1	LVEDD acc. to HENRY	0.005	0.103	0.989	-0.013	0.112	0.966	n < 50		
CO2	C2	Complement C2	LVEDD acc. to HENRY	0.072	0.053	0.737	0.087	0.059	0.616	-0.031	0.120	0.986
CO3	C3	Complement C3	LVEDD acc. to HENRY	-0.089	0.052	0.592	-0.127	0.058	0.440	0.059	0.120	0.985
CO4A	C4A	Complement C4-A	LVEDD acc. to HENRY	-0.045	0.052	0.908	-0.091	0.059	0.595	0.107	0.120	0.985
CO4B	C4B	Complement C4-B	LVEDD acc. to HENRY	-0.009	0.053	0.989	-0.026	0.059	0.966	0.080	0.120	0.985
CO5	C5	Complement C5	LVEDD acc. to HENRY	-0.070	0.052	0.761	-0.066	0.059	0.785	-0.090	0.120	0.985
CO6	C6	Complement component C6	LVEDD acc. to HENRY	0.008	0.052	0.989	0.007	0.059	0.966	0.013	0.120	0.986
CO7	C7	Complement component C7	LVEDD acc. to HENRY	0.052	0.052	0.867	0.052	0.059	0.879	0.049	0.120	0.986
CO8A	C8A	Complement component C8 alpha chain	LVEDD acc. to HENRY	-0.044	0.053	0.908	-0.085	0.059	0.645	0.109	0.120	0.985
CO8B	C8B	Complement component C8 beta chain	LVEDD acc. to HENRY	-0.029	0.053	0.989	-0.074	0.059	0.716	0.143	0.119	0.985
CO8G	C8G	Complement component C8 gamma chain	LVEDD acc. to HENRY	-0.091	0.053	0.592	-0.119	0.059	0.443	0.000	0.120	0.999
CO9	C9	Complement component C9 [C9a; C9b]	LVEDD acc. to HENRY	0.023	0.053	0.989	0.038	0.059	0.966	-0.047	0.120	0.986
COF1	CFL1	Cofilin-1	LVEDD acc. to HENRY	0.011	0.112	0.989	-0.044	0.122	0.966	n < 50		
COL11	COLEC11	Collectin-11	LVEDD acc. to HENRY	0.103	0.058	0.592	0.086	0.065	0.674	0.189	0.140	0.985
COMP	COMP	Cartilage oligomeric matrix protein	LVEDD acc. to HENRY	-0.039	0.052	0.926	-0.039	0.059	0.966	-0.044	0.124	0.986
CPN2	CPN2	Carboxypeptidase N subunit 2	LVEDD acc. to HENRY	-0.036	0.052	0.933	-0.026	0.059	0.966	-0.083	0.120	0.985
CRAC1	CRTAC1	Cartilage acidic protein 1	LVEDD acc. to HENRY	-0.203	0.095	0.553	-0.123	0.109	0.785	n < 50		
CRIS3	CRISP3	Cysteine-rich secretory protein 3	LVEDD acc. to HENRY	0.047	0.053	0.902	0.054	0.059	0.879	0.022	0.120	0.986
CRP	CRP	C-reactive protein [Cleaved into: C-reactive protein	LVEDD acc. to HENRY	-0.010	0.055	0.989	0.018	0.060	0.966	-0.153	0.131	0.985
CXCL7	PPBP	Platelet basic protein	LVEDD acc. to HENRY	-0.041	0.053	0.920	-0.024	0.059	0.966	-0.112	0.120	0.985
CYTC	CST3	Cystatin-C	LVEDD acc. to HENRY	-0.026	0.060	0.989	-0.037	0.067	0.966	0.026	0.139	0.986
DCD	DCD	Dermcidin	LVEDD acc. to HENRY	0.231	0.072	0.237	0.171	0.079	0.440	n < 50		
DEF3	DEFA3	Neutrophil defensin 3	LVEDD acc. to HENRY	-0.041	0.055	0.926	-0.052	0.061	0.886	0.013	0.127	0.986
DESP	DSP	Desmoplakin	LVEDD acc. to HENRY	-0.729	0.269	0.553	-0.662	0.294	0.564	-0.247	0.125	0.985
			LVEDD acc. to HENRY'	0.199	0.093		0.202	0.099				
			LVEDD acc. to HENRY''	-0.459	0.249		-0.515	0.284				
DIAC	CTBS	Di-N-acetylchitobiase	LVEDD acc. to HENRY	0.092	0.100	0.902	0.090	0.111	0.902	n < 50		
DOPO	DBH	Dopamine beta-hydroxylase	LVEDD acc. to HENRY	0.033	0.057	0.956	0.010	0.064	0.966	0.108	0.122	0.985
DPP4	DPP4	Dipeptidyl peptidase 4	LVEDD acc. to HENRY	0.099	0.140	0.931	-0.006	0.168	0.981	n < 50		
ECM1	ECM1	Extracellular matrix protein 1	LVEDD acc. to HENRY	0.032	0.053	0.955	0.009	0.059	0.966	0.133	0.119	0.985
EMAL6	EML6	Echinoderm microtubule-associated protein-like 6	LVEDD acc. to HENRY	-0.017	0.061	0.989	-0.019	0.069	0.966	-0.012	0.128	0.986
ENPP2	ENPP2	Ectonucleotide pyrophosphatase/phosphodiesterase family member 2	LVEDD acc. to HENRY	0.039	0.128	0.989	n < 50			n < 50		
EPCR	PROCR	Endothelial protein C receptor	LVEDD acc. to HENRY	-0.007	0.096	0.989	-0.002	0.102	0.988	n < 50		
F13A	F13A1	Coagulation factor XIII A chain	LVEDD acc. to HENRY	0.035	0.053	0.944	0.043	0.059	0.947	-0.009	0.120	0.986
F13B	F13B	Coagulation factor XIII B chain	LVEDD acc. to HENRY	0.006	0.053	0.989	-0.006	0.059	0.966	0.052	0.120	0.985
FA10	F10	Coagulation factor X	LVEDD acc. to HENRY	0.021	0.053	0.989	0.609	0.276	0.564	-0.088	0.120	0.985
			LVEDD acc. to HENRY'				-0.149	0.092				
			LVEDD acc. to HENRY''				0.347	0.263				
FA11	F11	Coagulation factor XI	LVEDD acc. to HENRY	-0.040	0.052	0.920	-0.077	0.059	0.674	0.183	0.118	0.985
FA12	F12	Coagulation factor XII	LVEDD acc. to HENRY	-0.043	0.053	0.908	-0.028	0.059	0.966	-0.105	0.120	0.985
FA5	F5	Coagulation factor V	LVEDD acc. to HENRY	-0.025	0.053	0.989	-0.026	0.059	0.966	-0.014	0.120	0.986
FA7	F7	Coagulation factor VII	LVEDD acc. to HENRY	-0.083	0.069	0.773	-0.117	0.081	0.650	n < 50		
FA9	F9	Coagulation factor IX	LVEDD acc. to HENRY	-0.066	0.053	0.772	-0.096	0.059	0.564	0.061	0.120	0.985
FBLN1	FBLN1	Fibulin-1	LVEDD acc. to HENRY	-0.013	0.052	0.989	-0.014	0.058	0.966	-0.011	0.120	0.986
FBLN3	EFEMP1	EGF-containing fibulin-like extracellular matrix protein 1	LVEDD acc. to HENRY	-0.010	0.053	0.989	-0.028	0.059	0.966	0.080	0.120	0.985
FBN1	FBN1	Fibrillin-1 [Cleaved into: Asprosin]	LVEDD acc. to HENRY	0.164	0.499	0.553	0.322	0.518	0.564	n < 50		
			LVEDD acc. to HENRY'	0.097	0.160		0.036	0.164				
			LVEDD acc. to HENRY''	-0.407	0.423		-0.272	0.463				
FCG3A	FCGR3A	Low affinity immunoglobulin gamma Fc region receptor III-A	LVEDD acc. to HENRY	0.017	0.079	0.989	0.027	0.088	0.966	n < 50		
FCGBP	FCGBP	IgGFc-binding protein	LVEDD acc. to HENRY	0.526	0.255	0.466	0.547	0.272	0.240	0.023	0.121	0.986
			LVEDD acc. to HENRY'	-0.225	0.087		-0.250	0.091				
			LVEDD acc. to HENRY''	0.582	0.234		0.707	0.260				
FCN3	FCN3	Ficolin-3	LVEDD acc. to HENRY	0.000	0.053	0.996	0.022	0.059	0.966	-0.126	0.119	0.985
FETUA	AHSG	Alpha-2-HS-glycoprotein	LVEDD acc. to HENRY	0.004	0.053	0.989	-0.001	0.059	0.988	0.026	0.120	0.986
FETUB	FETUB	Fetuin-B	LVEDD acc. to HENRY	0.026	0.052	0.989	0.080	0.058	0.674	-0.116	0.120	0.985
FGL1	FGL1	Fibrinogen-like protein 1	LVEDD acc. to HENRY	0.101	0.139	0.931	0.033	0.151	0.966	n < 50		
FHR1	CFHR1	Complement factor H-related protein 1	LVEDD acc. to HENRY	-0.129	0.052	0.490	-0.169	0.058	0.240	0.072	0.120	0.985
FHR2	CFHR2	Complement factor H-related protein 2	LVEDD acc. to HENRY	-0.087	0.053	0.592	-0.089	0.059	0.602	-0.080	0.120	0.985
FHR3	CFHR3	Complement factor H-related protein 3	LVEDD acc. to HENRY	-0.094	0.154	0.955	n < 50			n < 50		
FHR5	CFHR5	Complement factor H-related protein 5	LVEDD acc. to HENRY	-0.085	0.052	0.597	-0.104	0.058	0.535	0.011	0.120	0.986
FIBA	FGA	Fibrinogen alpha chain	LVEDD acc. to HENRY	-0.036	0.053	0.933	-0.052	0.059	0.879	0.042	0.120	0.986
FIBB	FGB	Fibrinogen beta chain	LVEDD acc. to HENRY	-0.032	0.053	0.955	-0.042	0.059	0.959	0.014	0.120	0.986
FIBG	FGG	Fibrinogen gamma chain	LVEDD acc. to HENRY	-0.021	0.053	0.989	-0.042	0.059	0.959	0.077	0.120	0.985
FINC	FN1	Fibronectin	LVEDD acc. to HENRY	-0.783	0.254	0.490	-0.914	0.273	0.240	-0.044	0.120	0.986
			LVEDD acc. to HENRY'	0.243	0.087		0.292	0.091				
			LVEDD acc. to HENRY''	-0.628	0.233		-0.824	0.260				
FRIL	FTL	Ferritin light chain	LVEDD acc. to HENRY	-0.019	0.101	0.989	0.005	0.103	0.981	n < 50		
G3P	GAPDH	Glyceraldehyde-3-phosphate dehydrogenase	LVEDD acc. to HENRY	-0.571	0.313	0.532	-0.482	0.329	0.564	n < 50		
			LVEDD acc. to HENRY'	0.252	0.108		0.221	0.111				
			LVEDD acc. to HENRY''	-0.758	0.293		-0.712	0.324				
GANAB	GANAB	Neutral alpha-glucosidase AB	LVEDD acc. to HENRY	0.030	0.088	0.989	0.018	0.099	0.966	n < 50		
GELS	GSN	Gelsolin	LVEDD acc. to HENRY	0.062	0.053	0.782	0.048	0.059	0.902	0.115	0.120	0.985
GGH	GGH	Gamma-glutamyl hydrolase	LVEDD acc. to HENRY	-0.081	0.068	0.773	-0.115	0.075	0.595	n < 50		
GP1BA	GP1BA	Platelet glycoprotein Ib alpha chain	LVEDD acc. to HENRY	0.027	0.056	0.989	0.006	0.062	0.966	0.117	0.126	0.985
GPX3	GPX3	Glutathione peroxidase 3	LVEDD acc. to HENRY	0.114	0.053	0.553	0.129	0.058	0.440	0.054	0.120	0.985
HABP2	HABP2	Hyaluronan-binding protein 2	LVEDD acc. to HENRY	0.080	0.052	0.644	0.079	0.059	0.674	0.088	0.120	0.985
HAT1	HAT1	Histone acetyltransferase type B catalytic subunit	LVEDD acc. to HENRY	0.099	0.082	0.773	0.139	0.090	0.595	n < 50		
HBA	HBA1; HBA2	Hemoglobin subunit alpha	LVEDD acc. to HENRY	-0.025	0.057	0.989	-0.057	0.062	0.879	0.133	0.140	0.985
HBB	HBB	Hemoglobin subunit beta	LVEDD acc. to HENRY	0.015	0.055	0.989	-0.012	0.061	0.966	0.151	0.132	0.985
HBG1	HBG1	Hemoglobin subunit gamma-1	LVEDD acc. to HENRY	-0.140	0.095	0.655	-0.128	0.106	0.740	n < 50		
HEMO	HPX	Hemopexin	LVEDD acc. to HENRY	-0.003	0.053	0.989	0.006	0.059	0.966	-0.048	0.120	0.986
HEP2	SERPIND1	Heparin cofactor 2	LVEDD acc. to HENRY	0.015	0.052	0.989	0.023	0.058	0.966	-0.020	0.120	0.986
HGFA	HGFAC	Hepatocyte growth factor activator	LVEDD acc. to HENRY	-0.014	0.053	0.989	0.023	0.059	0.966	-0.159	0.119	0.985
HGFL	MST1	Hepatocyte growth factor-like protein	LVEDD acc. to HENRY	0.042	0.054	0.920	0.291	0.276	0.443	0.133	0.121	0.985

			LVEDD acc. to HENRY'				-0.008	0.093				
			LVEDD acc. to HENRY''				-0.095	0.268				
HPT	HP	Haptoglobin	LVEDD acc. to HENRY	0.013	0.053	0.989	-0.028	0.059	0.966	0.120	0.120	0.985
HPTR	HPR	Haptoglobin-related protein	LVEDD acc. to HENRY	-0.065	0.053	0.773	-0.081	0.059	0.674	-0.011	0.121	0.986
HRG	HRG	Histidine-rich glycoprotein	LVEDD acc. to HENRY	0.011	0.053	0.989	-0.024	0.059	0.966	0.153	0.119	0.985
HV118	IGHV1-18	Immunoglobulin heavy variable 1-18	LVEDD acc. to HENRY	0.102	0.080	0.771	0.051	0.089	0.966	n < 50		
HV146	IGHV1-46	Immunoglobulin heavy variable 1-46	LVEDD acc. to HENRY	0.396	0.282	0.237	0.435	0.302	0.240	0.105	0.137	0.985
			LVEDD acc. to HENRY'	-0.044	0.098		-0.085	0.103				
			LVEDD acc. to HENRY''	-0.032	0.263		0.098	0.296				
HV307	IGHV3-7	Immunoglobulin heavy variable 3-7	LVEDD acc. to HENRY	0.100	0.115	0.908	0.017	0.124	0.966	n < 50		
HV372	IGHV3-72	Immunoglobulin heavy variable 3-72	LVEDD acc. to HENRY	-0.020	0.078	0.989	-0.019	0.083	0.966	n < 50		
HV374	IGHV3-74	Immunoglobulin heavy variable 3-74	LVEDD acc. to HENRY	0.000	0.063	0.996	-0.015	0.069	0.966	n < 50		
HV551	IGHV5-51	Immunoglobulin heavy variable 5-51	LVEDD acc. to HENRY	0.030	0.124	0.989	-0.072	0.134	0.966	n < 50		
HVC05	IGHV3-30-5	Immunoglobulin heavy variable 3-30-5	LVEDD acc. to HENRY	-0.106	0.108	0.867	-0.120	0.116	0.831	n < 50		
HYDIN	HYDIN	Hydrocephalus-inducing protein homolog	LVEDD acc. to HENRY	-0.043	0.053	0.908	-0.063	0.059	0.806	0.037	0.120	0.986
IBP2	IGFBP2	Insulin-like growth factor-binding protein 2	LVEDD acc. to HENRY	-0.004	0.076	0.989	0.010	0.083	0.966	n < 50		
IBP3	IGFBP3	Insulin-like growth factor-binding protein 3	LVEDD acc. to HENRY	0.020	0.053	0.989	0.027	0.059	0.966	-0.012	0.120	0.986
IBP4	IGFBP4	Insulin-like growth factor-binding protein 4	LVEDD acc. to HENRY	0.016	0.054	0.989	-0.021	0.060	0.966	0.188	0.120	0.985
IBP6	IGFBP6	Insulin-like growth factor-binding protein 6	LVEDD acc. to HENRY	-0.079	0.073	0.827	-0.066	0.082	0.902	n < 50		
IC1	SERPING1	Plasma protease C1 inhibitor	LVEDD acc. to HENRY	-0.001	0.053	0.996	0.006	0.059	0.966	-0.032	0.120	0.986
ICAM2	ICAM2	Intercellular adhesion molecule 2	LVEDD acc. to HENRY	-0.245	0.154	0.621	n < 50			n < 50		
IGA2	IGA2*	Immunoglobulin alpha-2 heavy chain	LVEDD acc. to HENRY	0.381	0.260	0.592	-0.028	0.059	0.966	0.120	0.120	0.985
			LVEDD acc. to HENRY'	-0.072	0.089							
			LVEDD acc. to HENRY''	0.107	0.237							
IGD	IGD*	Immunoglobulin delta heavy chain	LVEDD acc. to HENRY	0.080	0.111	0.931	-0.043	0.563	0.535	n < 50		
			LVEDD acc. to HENRY'				0.197	0.187				
			LVEDD acc. to HENRY''				-0.792	0.555				
IGF1	IGF1	Insulin-like growth factor I	LVEDD acc. to HENRY	-0.098	0.061	0.598	-0.096	0.067	0.650	-0.108	0.148	0.985
IGF2	IGF2	Insulin-like growth factor II	LVEDD acc. to HENRY	0.065	0.101	0.955	0.052	0.112	0.966	n < 50		
IGG1	IGG1	Immunoglobulin gamma-1 heavy chain	LVEDD acc. to HENRY	-0.482	0.258	0.592	-0.609	0.276	0.564	0.046	0.120	0.986
			LVEDD acc. to HENRY'	0.214	0.088		0.230	0.092				
			LVEDD acc. to HENRY''	-0.603	0.236		-0.659	0.264				
IGHA1	IGHA1	Immunoglobulin heavy constant alpha 1	LVEDD acc. to HENRY	0.012	0.053	0.989	0.006	0.059	0.966	0.036	0.121	0.986
IGHG2	IGHG2	Immunoglobulin heavy constant gamma 2	LVEDD acc. to HENRY	0.069	0.053	0.771	-0.537	0.278	0.443	0.053	0.120	0.985
			LVEDD acc. to HENRY'				0.234	0.092				
			LVEDD acc. to HENRY''				-0.684	0.264				
IGHG3	IGHG3	Immunoglobulin heavy constant gamma 3	LVEDD acc. to HENRY	-0.009	0.053	0.989	-0.011	0.059	0.966	0.002	0.120	0.995
IGHG4	IGHG4	Immunoglobulin heavy constant gamma 4	LVEDD acc. to HENRY	0.099	0.060	0.592	0.089	0.066	0.674	0.151	0.141	0.985
IGHM	IGHM	Immunoglobulin heavy constant mu	LVEDD acc. to HENRY	-0.078	0.053	0.655	-0.084	0.059	0.650	-0.054	0.120	0.985
IGJ	JCHAIN	Immunoglobulin J chain	LVEDD acc. to HENRY	-0.077	0.061	0.771	-0.108	0.068	0.564	0.031	0.136	0.986
IGK	IGK*	Immunoglobulin kappa light chain	LVEDD acc. to HENRY	-0.077	0.257	0.544	-0.236	0.275	0.443	0.067	0.120	0.985
			LVEDD acc. to HENRY'	0.122	0.088		0.167	0.092				
			LVEDD acc. to HENRY''	-0.410	0.236		-0.557	0.262				
IGKC	IGKC	Immunoglobulin kappa constant	LVEDD acc. to HENRY	0.019	0.053	0.989	0.010	0.059	0.966	0.052	0.120	0.985
IGL1	IGL1*	Immunoglobulin lambda-1 light chain	LVEDD acc. to HENRY	-0.038	0.054	0.931	-0.028	0.061	0.966	-0.079	0.123	0.985
IGLC3	IGLC3	Immunoglobulin lambda constant 3	LVEDD acc. to HENRY	-0.018	0.053	0.989	-0.033	0.059	0.966	0.046	0.120	0.986
IL1AP	IL1RAP	Interleukin-1 receptor accessory protein	LVEDD acc. to HENRY	0.126	0.082	0.644	0.078	0.097	0.902	n < 50		
ILK	ILK	Integrin-linked protein kinase	LVEDD acc. to HENRY	-0.114	0.103	0.821	-0.145	0.120	0.740	n < 50		
INHBC	INHBC	Inhibin beta C chain	LVEDD acc. to HENRY	0.018	0.077	0.989	-0.006	0.087	0.972	n < 50		
IPSP	SERPINA5	Plasma serine protease inhibitor	LVEDD acc. to HENRY	-0.007	0.053	0.989	-0.005	0.059	0.966	-0.014	0.120	0.986
ITB1	ITGB1	Integrin beta-1	LVEDD acc. to HENRY	-0.149	0.145	0.867	n < 50			n < 50		
ITIH1	ITIH1	Inter-alpha-trypsin inhibitor heavy chain H1	LVEDD acc. to HENRY	-0.043	0.053	0.908	-0.037	0.059	0.966	-0.073	0.120	0.985
ITIH2	ITIH2	Inter-alpha-trypsin inhibitor heavy chain H2	LVEDD acc. to HENRY	-0.064	0.053	0.773	-0.090	0.059	0.595	0.062	0.120	0.985
ITIH3	ITIH3	Inter-alpha-trypsin inhibitor heavy chain H3	LVEDD acc. to HENRY	-0.011	0.053	0.989	-0.016	0.059	0.966	0.010	0.120	0.986
ITIH4	ITIH4	Inter-alpha-trypsin inhibitor heavy chain H4	LVEDD acc. to HENRY	0.089	0.052	0.592	0.143	0.058	0.401	-0.123	0.119	0.985
K1C9	KRT9	Keratin, type I cytoskeletal 9	LVEDD acc. to HENRY	-0.052	0.053	0.867	-0.037	0.059	0.966	-1.367	0.556	0.985
			LVEDD acc. to HENRY'							0.427	0.175	
			LVEDD acc. to HENRY''							-1.210	0.503	
K2C1	KRT1	Keratin, type II cytoskeletal 1	LVEDD acc. to HENRY	-0.061	0.053	0.784	-0.056	0.059	0.866	-1.467	0.551	0.985
			LVEDD acc. to HENRY'							0.479	0.173	
			LVEDD acc. to HENRY''							-1.370	0.499	
KAIN	SERPINA4	Kallistatin	LVEDD acc. to HENRY	0.037	0.053	0.931	0.037	0.059	0.966	0.040	0.120	0.986
KLKB1	KLKB1	Plasma kallikrein	LVEDD acc. to HENRY	-0.018	0.052	0.989	-0.005	0.059	0.966	-0.077	0.120	0.985
KNG1	KNG1	Kininogen-1	LVEDD acc. to HENRY	-0.064	0.052	0.773	-0.080	0.059	0.674	-0.004	0.120	0.995
KV106	IGKV1-6	Immunoglobulin kappa variable 1-6	LVEDD acc. to HENRY	0.009	0.147	0.989	n < 50			n < 50		
KV320	IGKV3-20	Immunoglobulin kappa variable 3-20	LVEDD acc. to HENRY	-0.025	0.062	0.989	-0.078	0.067	0.770	n < 50		
KV401	IGKV4-1	Immunoglobulin kappa variable 4-1	LVEDD acc. to HENRY	-0.346	0.291	0.597	-0.345	0.300	0.440	-0.041	0.152	0.986
			LVEDD acc. to HENRY'	0.188	0.101		0.209	0.102				
			LVEDD acc. to HENRY''	-0.570	0.275		-0.702	0.297				
KVD11	IGKV3D-11	Immunoglobulin kappa variable 3D-11	LVEDD acc. to HENRY	-1.812	0.692	0.553	n < 50			n < 50		
			LVEDD acc. to HENRY'	0.560	0.225							
			LVEDD acc. to HENRY''	-1.462	0.589							
KVD15	IGKV3D-15	Immunoglobulin kappa variable 3D-15	LVEDD acc. to HENRY	0.142	0.082	0.592	0.144	0.090	0.564	n < 50		
LBP	LBP	Lipopolysaccharide-binding protein	LVEDD acc. to HENRY	-0.008	0.053	0.989	-0.034	0.059	0.966	0.093	0.120	0.985
LCAT	LCAT	Phosphatidylcholine-sterol acyltransferase	LVEDD acc. to HENRY	0.014	0.052	0.989	0.009	0.059	0.966	-0.316	0.557	0.985
			LVEDD acc. to HENRY'							-0.031	0.175	
			LVEDD acc. to HENRY''							0.295	0.504	
LDHB	LDHB	L-lactate dehydrogenase B chain	LVEDD acc. to HENRY	0.058	0.068	0.908	0.041	0.075	0.966	n < 50		
LG3BP	LGALS3BP	Galectin-3-binding protein	LVEDD acc. to HENRY	-0.031	0.053	0.956	-0.013	0.059	0.966	-0.110	0.120	0.985
LRP1	LRP1	Prolow-density lipoprotein receptor-related protein 1	LVEDD acc. to HENRY	-0.057	0.074	0.920	-0.073	0.082	0.879	n < 50		
LUM	LUM	Lumican	LVEDD acc. to HENRY	0.089	0.052	0.592	0.118	0.058	0.443	-0.034	0.120	0.986
LV147	IGLV1-47	Immunoglobulin lambda variable 1-47	LVEDD acc. to HENRY	0.000	0.090	0.996	-0.054	0.096	0.966	n < 50		
LV319	IGLV3-19	Immunoglobulin lambda variable 3-19	LVEDD acc. to HENRY	-0.043	0.073	0.956	-0.083	0.079	0.820	n < 50		
LV321	IGLV3-21	Immunoglobulin lambda variable 3-21	LVEDD acc. to HENRY	0.013	0.064	0.989	-0.018	0.070	0.966	n < 50		
LV861	IGLV8-61	Immunoglobulin lambda variable 8-61	LVEDD acc. to HENRY	0.149	0.133	0.817	n < 50			n < 50		
LYAM1	SELL	L-selectin	LVEDD acc. to HENRY	0.044	0.053	0.908	0.061	0.059	0.831	-0.011	0.120	0.986
LYSC	LYZ	Lysozyme C	LVEDD acc. to HENRY	-0.005	0.053	0.989	0.015	0.059	0.966	-0.085	0.120	0.985
LYVE1	LYVE1	Lymphatic vessel endothelial hyaluronic acid receptor 1	LVEDD acc. to HENRY	-0.209	0.112	0.592	-0.753	0.498	0.401	n < 50		
			LVEDD acc. to HENRY'				0.039	0.176				
			LVEDD acc. to HENRY''				0.092	0.518				
MARCO	MARCO	Macrophage receptor MARCO	LVEDD acc. to HENRY	0.101	0.121	0.908	0.093	0.129	0.959	n < 50		
MASP1	MASP1	Mannan-binding lectin serine protease 1	LVEDD acc. to HENRY	0.086	0.053	0.592	0.106	0.059	0.535	0.008	0.120	0.986
MASP2	MASP2	Mannan-binding lectin serine protease 2	LVEDD acc. to HENRY	-0.120	0.058	0.553	-0.074	0.066	0.785	-0.276	0.127	0.985

MAST4	MAST4	Microtubule-associated serine/threonine-protein kinase 4	LVEDD acc. to HENRY	-0.045	0.152	0.989	n < 50			n < 50		
MBL2	MBL2	Mannose-binding protein C	LVEDD acc. to HENRY	0.010	0.059	0.989	-0.006	0.065	0.966	0.084	0.145	0.985
MMP2	MMP2	72 kDa type IV collagenase	LVEDD acc. to HENRY	0.181	0.079	0.544	0.147	0.087	0.564	n < 50		
MMRN2	MMRN2	Multimerin-2	LVEDD acc. to HENRY	-0.066	0.141	0.989	-0.200	0.148	0.674	n < 50		
MTBP	MTBP	Mdm2-binding protein	LVEDD acc. to HENRY	-0.085	0.107	0.920	-0.044	0.113	0.966	n < 50		
MUC18	MCAM	Cell surface glycoprotein MUC18	LVEDD acc. to HENRY	0.127	0.063	0.553	0.084	0.070	0.740	n < 50		
MUC19	MUC19	Mucin-19	LVEDD acc. to HENRY	-0.091	0.054	0.592	-0.108	0.060	0.535	-0.013	0.120	0.986
MYH4	MYH4	Myosin-4	LVEDD acc. to HENRY	-0.039	0.077	0.989	-0.032	0.083	0.966	n < 50		
NCAM1	NCAM1	Neural cell adhesion molecule 1	LVEDD acc. to HENRY	0.305	0.339	0.532	0.467	0.365	0.440	n < 50		
			LVEDD acc. to HENRY'	0.033	0.118		-0.029	0.122				
			LVEDD acc. to HENRY''	-0.219	0.316		-0.067	0.348				
NCHL1	CHL1	Neural cell adhesion molecule L1-like protein	LVEDD acc. to HENRY	0.070	0.093	0.926	0.011	0.104	0.966	n < 50		
NOE1	OLFM1	Noelin	LVEDD acc. to HENRY	-0.038	0.115	0.989	0.035	0.136	0.966	n < 50		
NOTC3	NOTCH3	Neurogenic locus notch homolog protein 3	LVEDD acc. to HENRY	-0.231	0.536	0.466	-0.149	0.549	0.443	n < 50		
			LVEDD acc. to HENRY'	0.176	0.184		0.132	0.184				
			LVEDD acc. to HENRY''	-0.674	0.497		-0.592	0.535				
NRP1	NRP1	Neuropilin-1	LVEDD acc. to HENRY	0.046	0.107	0.989	-0.024	0.119	0.966	n < 50		
OR3A2	OR3A2	Olfactory receptor 3A2	LVEDD acc. to HENRY	0.010	0.054	0.989	-0.015	0.060	0.966	0.115	0.121	0.985
P4HA3	P4HA3	Prolyl 4-hydroxylase subunit alpha-3	LVEDD acc. to HENRY	-0.053	0.077	0.933	-0.112	0.085	0.674	n < 50		
PAFA	PLA2G7	Platelet-activating factor acetylhydrolase	LVEDD acc. to HENRY	0.118	0.082	0.672	0.150	0.086	0.564	n < 50		
PARP9	PARP9	Protein mono-ADP-ribosyltransferase PARP9	LVEDD acc. to HENRY	0.092	0.076	0.773	0.076	0.085	0.879	n < 50		
PCOC1	PCOLCE	Procollagen C-endopeptidase enhancer 1	LVEDD acc. to HENRY	-0.028	0.056	0.989	-0.069	0.062	0.788	0.433	0.583	0.985
			LVEDD acc. to HENRY'							-0.234	0.183	
			LVEDD acc. to HENRY''							0.840	0.525	
PCYOX	PCYOX1	Prenylcysteine oxidase 1	LVEDD acc. to HENRY	0.055	0.053	0.856	0.068	0.059	0.770	-0.005	0.120	0.995
PEDF	SERPINF1	Pigment epithelium-derived factor	LVEDD acc. to HENRY	-0.135	0.052	0.466	-0.105	0.059	0.535	-0.260	0.116	0.985
PEPD	PEPD	Xaa-Pro dipeptidase	LVEDD acc. to HENRY	-0.035	0.140	0.989	n < 50			n < 50		
PGBM	HSPG2	Basement membrane-specific heparan sulfate proteoglycan core protein	LVEDD acc. to HENRY	-0.015	0.056	0.989	-0.028	0.062	0.966	0.061	0.131	0.985
PGRP2	PGLYRP2	N-acetylmuramoyl-L-alanine amidase	LVEDD acc. to HENRY	-0.048	0.053	0.902	-0.097	0.059	0.564	0.105	0.120	0.985
PHLD	GPLD1	Phosphatidylinositol-glycan-specific phospholipase D	LVEDD acc. to HENRY	0.011	0.053	0.989	0.038	0.059	0.966	-0.122	0.119	0.985
PI16	PI16	Peptidase inhibitor 16	LVEDD acc. to HENRY	0.005	0.054	0.989	-0.052	0.060	0.886	0.228	0.121	0.985
PLAK	JUP	Junction plakoglobin	LVEDD acc. to HENRY	-0.105	0.080	0.770	-0.120	0.085	0.674	n < 50		
PLF4	PF4	Platelet factor 4	LVEDD acc. to HENRY	0.020	0.054	0.989	0.036	0.059	0.966	-0.059	0.128	0.985
PLMN	PLG	Plasminogen	LVEDD acc. to HENRY	-0.075	0.052	0.655	-0.117	0.059	0.451	0.085	0.120	0.985
PLSL	LCP1	Plastin-2	LVEDD acc. to HENRY	0.059	0.058	0.867	0.070	0.064	0.801	0.004	0.136	0.995
PLTP	PLTP	Phospholipid transfer protein	LVEDD acc. to HENRY	0.035	0.053	0.944	0.027	0.059	0.966	0.078	0.120	0.985
PON1	PON1	Serum paraoxonase/arylesterase 1	LVEDD acc. to HENRY	-0.062	0.053	0.777	-0.057	0.059	0.848	-0.084	0.120	0.985
PON3	PON3	Serum paraoxonase/lactonase 3	LVEDD acc. to HENRY	0.112	0.066	0.592	0.125	0.073	0.564	n < 50		
PRDX2	PRDX2	Peroxiredoxin-2	LVEDD acc. to HENRY	0.035	0.087	0.989	0.047	0.094	0.966	n < 50		
PRG2	PRG2	Bone marrow proteoglycan	LVEDD acc. to HENRY	-0.006	0.116	0.989	-0.123	0.132	0.879	n < 50		
PRG4	PRG4	Proteoglycan 4	LVEDD acc. to HENRY	-0.017	0.053	0.989	0.007	0.059	0.966	0.488	0.553	0.985
			LVEDD acc. to HENRY'							-0.316	0.174	
			LVEDD acc. to HENRY''							1.046	0.500	
PROC	PROC	Vitamin K-dependent protein C	LVEDD acc. to HENRY	-0.018	0.052	0.989	-0.011	0.058	0.966	-0.057	0.120	0.985
PROF1	PFN1	Profilin-1	LVEDD acc. to HENRY	-0.054	0.079	0.933	-0.103	0.085	0.740	n < 50		
PROP	CFP	Properdin	LVEDD acc. to HENRY	0.189	0.257	0.592	0.388	0.274	0.443	0.070	0.120	0.985
			LVEDD acc. to HENRY'	-0.142	0.088		-0.207	0.091				
			LVEDD acc. to HENRY''	0.443	0.236		0.645	0.261				
PROS	PROS1	Vitamin K-dependent protein S	LVEDD acc. to HENRY	-0.028	0.053	0.989	-0.063	0.059	0.811	0.136	0.119	0.985
PROZ	PROZ	Vitamin K-dependent protein Z	LVEDD acc. to HENRY	-0.048	0.057	0.908	-0.043	0.064	0.966	-0.071	0.122	0.985
PSB1	PSMB1	Proteasome subunit beta type-1	LVEDD acc. to HENRY	-0.141	0.092	0.644	-0.138	0.101	0.674	n < 50		
PSPB	SFTPB	Pulmonary surfactant-associated protein B	LVEDD acc. to HENRY	-0.011	0.129	0.989	n < 50			n < 50		
PTGDS	PTGDS	Prostaglandin-H2 D-isomerase	LVEDD acc. to HENRY	-0.136	0.069	0.553	-0.191	0.077	0.401	n < 50		
PTMA	PTMA	Prothymosin alpha [Cleaved into: Prothymosin alpha, N-terminally processed; Thymosin alpha-1]	LVEDD acc. to HENRY	-0.049	0.066	0.930	-0.054	0.070	0.923	n < 50		
PVR	PVR	Poliovirus receptor	LVEDD acc. to HENRY	-0.039	0.149	0.989	n < 50			n < 50		
PXDC2	PLXDC2	Plexin domain-containing protein 2	LVEDD acc. to HENRY	1.125	0.432	0.592	1.022	0.450	0.564	n < 50		
			LVEDD acc. to HENRY'	-0.386	0.143		-0.359	0.144				
			LVEDD acc. to HENRY''	0.986	0.374		0.983	0.401				
PZP	PZP	Pregnancy zone protein	LVEDD acc. to HENRY	-0.026	0.050	0.989	0.005	0.069	0.969	-0.160	0.120	0.985
QSOX1	QSOX1	Sulfhydryl oxidase 1	LVEDD acc. to HENRY	-0.020	0.051	0.989	-0.005	0.059	0.966	-0.084	0.120	0.985
RADX	RADX	RPA-related protein RADX	LVEDD acc. to HENRY	0.138	0.121	0.811	n < 50			n < 50		
RARR2	RARRES2	Retinoic acid receptor responder protein 2	LVEDD acc. to HENRY	-0.055	0.053	0.854	-0.071	0.059	0.740	0.016	0.120	0.986
RET4	RBP4	Retinol-binding protein 4	LVEDD acc. to HENRY	0.003	0.053	0.989	-0.018	0.059	0.966	0.106	0.120	0.985
RN112	RNF112	RING finger protein 112	LVEDD acc. to HENRY	-0.017	0.056	0.989	-0.041	0.061	0.966	0.078	0.129	0.985
RNAS1	RNASE1	Ribonuclease pancreatic	LVEDD acc. to HENRY	0.018	0.053	0.989	-0.008	0.059	0.966	0.151	0.121	0.985
RNAS4	RNASE4	Ribonuclease 4	LVEDD acc. to HENRY	0.016	0.141	0.989	n < 50			n < 50		
S10A9	S100A9	Protein S100-A9	LVEDD acc. to HENRY	0.010	0.076	0.989	0.027	0.085	0.966	n < 50		
SAA1	SAA1	Serum amyloid A-1 protein	LVEDD acc. to HENRY	-0.018	0.053	0.989	-0.034	0.059	0.966	0.056	0.121	0.985
SAA2	SAA2	Serum amyloid A-2 protein	LVEDD acc. to HENRY	0.007	0.053	0.989	-0.012	0.060	0.966	0.104	0.121	0.985
SAA4	SAA4	Serum amyloid A-4 protein	LVEDD acc. to HENRY	-0.019	0.052	0.989	-0.010	0.059	0.966	-0.057	0.120	0.985
SAMP	APCS	Serum amyloid P-component	LVEDD acc. to HENRY	-0.017	0.052	0.989	-0.025	0.059	0.966	0.021	0.120	0.986
SEPP1	SELENOP	Selenoprotein P	LVEDD acc. to HENRY	0.078	0.053	0.655	0.030	0.059	0.966	0.288	0.115	0.985
1433S	SFN	14-3-3 protein sigma	LVEDD acc. to HENRY	0.047	0.087	0.989	-0.775	0.426	0.564	n < 50		
			LVEDD acc. to HENRY'				0.346	0.145				
			LVEDD acc. to HENRY''				-1.024	0.415				
SHBG	SHBG	Sex hormone-binding globulin	LVEDD acc. to HENRY	-0.033	0.051	0.948	-0.046	0.059	0.923	0.012	0.121	0.986
SODE	SOD3	Extracellular superoxide dismutase [Cu-Zn]	LVEDD acc. to HENRY	-0.074	0.104	0.931	-0.037	0.120	0.966	n < 50		
SPB3	SERPINB3	Serpin B3	LVEDD acc. to HENRY	-0.060	0.096	0.955	-0.024	0.101	0.966	n < 50		
SPRL1	SPARCL1	SPARC-like protein 1	LVEDD acc. to HENRY	-0.067	0.073	0.902	-0.051	0.083	0.966	n < 50		
TAGL2	TAGLN2	Transgelin-2	LVEDD acc. to HENRY	0.144	0.098	0.655	0.112	0.112	0.841	n < 50		
TENA	TNC	Tenascin	LVEDD acc. to HENRY	-0.061	0.139	0.989	-0.047	0.143	0.966	n < 50		
TENX	TNXB	Tenascin-X	LVEDD acc. to HENRY	-0.066	0.082	0.909	-0.070	0.087	0.902	n < 50		
TETN	CLEC3B	Tetranectin	LVEDD acc. to HENRY	0.091	0.053	0.592	0.097	0.059	0.564	0.065	0.120	0.985
THBG	SERPINA7	Thyroxine-binding globulin	LVEDD acc. to HENRY	0.001	0.052	0.996	0.015	0.059	0.966	-0.054	0.120	0.985
THRB	F2	Prothrombin	LVEDD acc. to HENRY	0.032	0.053	0.955	0.687	0.276	0.535	-1.175	0.556	0.985
			LVEDD acc. to HENRY'				-0.188	0.092		0.296	0.175	
			LVEDD acc. to HENRY''				0.467	0.263		-0.678	0.503	
TIMP1	TIMP1	Metalloproteinase inhibitor 1	LVEDD acc. to HENRY	-0.135	0.123	0.821	n < 50			n < 50		
TLN1	TLN1	Talin-1	LVEDD acc. to HENRY	0.047	0.090	0.989	0.067	0.095	0.961	n < 50		
TRFE	TF	Serotransferrin	LVEDD acc. to HENRY	0.011	0.053	0.989	0.023	0.059	0.966	-0.050	0.120	0.986

TSP1	THBS1	Thrombospondin-1	LVEDD acc. to HENRY	-0.008	0.054	0.989	0.014	0.060	0.966	-0.109	0.125	0.985
TSP4	THBS4	Thrombospondin-4	LVEDD acc. to HENRY	0.099	0.060	0.592	0.162	0.068	0.401	-0.166	0.126	0.985
TTHY	TTR	Transthyretin	LVEDD acc. to HENRY	0.044	0.053	0.908	0.034	0.059	0.966	0.088	0.120	0.985
URP2	FERMT3	Fermitin family homolog 3	LVEDD acc. to HENRY	-0.021	0.111	0.989	-0.023	0.120	0.966	n < 50		
VASN	VASN	Vasorin	LVEDD acc. to HENRY	-0.001	0.055	0.996	0.013	0.061	0.966	-0.064	0.123	0.985
VASP	VASP	Vasodilator-stimulated phosphoprotein	LVEDD acc. to HENRY	0.062	0.390	0.553	0.092	0.093	0.842	n < 50		
			LVEDD acc. to HENRY'	-0.114	0.137							
			LVEDD acc. to HENRY''	0.433	0.368							
VCAM1	VCAM1	Vascular cell adhesion protein 1	LVEDD acc. to HENRY	0.027	0.068	0.989	0.020	0.073	0.966	n < 50		
VINC	VCL	Vinculin	LVEDD acc. to HENRY	-0.160	0.126	0.771	n < 50			n < 50		
VNN1	VNN1	Pantetheinase	LVEDD acc. to HENRY	0.052	0.054	0.873	0.017	0.061	0.966	0.189	0.118	0.985
VTDB	GC	Vitamin D-binding protein	LVEDD acc. to HENRY	-0.052	0.052	0.867	-0.057	0.059	0.851	-0.042	0.120	0.986
VTNC	VTN	Vitronectin	LVEDD acc. to HENRY	0.005	0.053	0.989	-0.018	0.059	0.966	0.096	0.120	0.985
VWF	VWF	von Willebrand factor	LVEDD acc. to HENRY	-0.078	0.053	0.655	-0.055	0.059	0.874	-0.191	0.118	0.985
1433Z	YWHAZ	14-3-3 protein zeta/delta	LVEDD acc. to HENRY	0.044	0.119	0.989	0.032	0.137	0.966	n < 50		
ZA2G	AZGP1	Zinc-alpha-2-glycoprotein	LVEDD acc. to HENRY	0.068	0.053	0.771	0.113	0.059	0.504	-0.116	0.120	0.985
ZPI	SERPINA10	Protein Z-dependent protease inhibitor	LVEDD acc. to HENRY	-0.015	0.053	0.989	0.013	0.059	0.966	-0.153	0.119	0.985

Supplemental Table S4. Associations of left ventricular ejection fraction (LVEF) and the plasma proteins measured using Olink (targeted approach). Results from regression models adjusted for sex, age and BMI for the whole study population and from sex-specific models adjusted for age and BMI. Proteins were log2-transformed. Estimates are presented for z-transformed outcome and exposure variables.
stderr, standard error; FDR, false discovery rate

Outcome			Exposure	All subjects			Men			Women		
				beta	stderr	FDR	beta	stderr	FDR	beta	stderr	FDR
OLINK Label	Gene name	Protein name										
**CVD2 Panel**												
ACE2	ACE2	Angiotensin-converting enzyme 2	LVEF	**-0.151**	**0.052**	**0.023**	**-0.175**	**0.061**	**0.020**	-0.070	0.121	0.977
ADAM_TS13	ADAMTS13	A disintegrin and metalloproteinase with thrombospondin motifs 13	LVEF	-0.003	0.055	0.986	0.013	0.061	0.897	-0.116	0.125	0.977
ADM	ADM	ADM [Cleaved into: Adrenomedullin]	LVEF	**-0.264**	**0.048**	**<0.001**	**-0.325**	**0.057**	**<0.001**	-0.033	0.105	0.977
AGRP	AGRP	Agouti-related protein	LVEF	-0.050	0.050	0.494	-0.047	0.061	0.585	-0.043	0.123	0.977
AMBP	AMBP	Protein AMBP [Cleaved into: Alpha-1-microglobulin]	LVEF	0.135	0.061	0.098	0.135	0.061	0.082	0.011	0.118	0.988
ANGPT1	ANGPT1	Angiopoietin-1	LVEF	-0.025	0.052	0.788	-0.052	0.062	0.551	0.076	0.125	0.977
BMP_6	BMP6	Bone morphogenetic protein 6	LVEF	**-0.194**	**0.043**	**<0.001**	**-0.301**	**0.060**	**<0.001**	0.019	0.118	0.982
BNP	NPPB	Natriuretic peptides B	LVEF	**-0.354**	**0.036**	**<0.001**	**-0.497**	**0.052**	**<0.001**	-0.257	0.115	0.595
BOC	BOC	Brother of CDO	LVEF	**-0.169**	**0.058**	**0.022**	**-0.193**	**0.060**	**0.008**	0.055	0.127	0.977
CA5A	CA5A	Carbonic anhydrase 5A, mitochondrial	LVEF	-0.012	0.059	0.942	-0.005	0.063	0.958	-0.006	0.120	0.989
CCL17	CCL17	C-C motif chemokine 17	LVEF	0.025	0.054	0.794	0.010	0.063	0.925	0.140	0.124	0.948
CCL3	CCL3	C-C motif chemokine 3	LVEF	-0.116	0.055	0.110	**-0.154**	**0.061**	**0.047**	0.119	0.111	0.948
CD40_L	CD40LG	CD40 ligand	LVEF	0.049	0.050	0.494	0.039	0.062	0.679	0.124	0.125	0.972
CD4	CD4	T-cell surface glycoprotein CD4	LVEF	**-0.197**	**0.052**	**0.002**	**-0.232**	**0.061**	**0.001**	-0.021	0.109	0.982
CD84	CD84	SLAM family member 5	LVEF	-0.030	0.056	0.760	-0.059	0.062	0.500	0.098	0.124	0.977
CEACAM8	CEACAM8	Carcinoembryonic antigen-related cell adhesion molecule 8	LVEF	-0.078	0.060	0.348	-0.087	0.062	0.268	0.011	0.126	0.988
CTRC	CTRC	Chymotrypsin-C	LVEF	0.017	0.061	0.901	0.021	0.062	0.833	-0.057	0.125	0.977
CTSL1	CTSL	Cathepsin L1	LVEF	**-0.228**	**0.057**	**0.001**	**-0.232**	**0.059**	**0.001**	-0.032	0.107	0.977
CXCL1	CXCL1	Growth-regulated alpha protein	LVEF	-0.006	0.047	0.952	-0.019	0.062	0.857	0.065	0.126	0.977
DCN	DCN	Decorin	LVEF	-0.115	0.050	0.084	-0.127	0.058	0.088	-0.017	0.100	0.982
DECR1	DECR1	2,4-dienoyl-CoA reductase, mitochondrial	LVEF	-0.045	0.045	0.488	-0.056	0.063	0.513	-0.029	0.124	0.977
Dkk_1	DKK1	Dickkopf-related protein 1	LVEF	-0.043	0.057	0.636	-0.073	0.062	0.377	0.087	0.127	0.977
FABP2	FABP2	Fatty acid-binding protein, intestinal	LVEF	-0.018	0.058	0.889	-0.017	0.063	0.880	-0.034	0.126	0.977
FGF_21	FGF21	Fibroblast growth factor 21	LVEF	**-0.191**	**0.055**	**0.005**	**-0.231**	**0.060**	**0.001**	-0.003	0.124	0.991
FGF_23	FGF23	Fibroblast growth factor 23	LVEF	**-0.372**	**0.065**	**<0.001**	**-0.342**	**0.058**	**<0.001**	-0.089	0.124	0.977
FS	FST	Follistatin	LVEF	-0.119	0.053	0.092	-0.124	0.062	0.120	-0.140	0.125	0.948
GDF_2	GDF2	Growth/differentiation factor 2	LVEF	-0.066	0.053	0.370	-0.058	0.062	0.506	-0.120	0.120	0.972
GH	GH1	Somatotropin	LVEF	-0.101	0.046	0.102	-0.123	0.061	0.114	-0.107	0.113	0.977
GIF	CBLIF	Gastric intrinsic factor	LVEF	0.148	0.059	0.057	0.141	0.059	0.064	0.092	0.125	0.977
GLO1	GLO1	Lactoylglutathione lyase	LVEF	0.002	0.053	0.987	0.011	0.061	0.912	-0.036	0.127	0.977
GT	FABP6	Gastrotropin	LVEF	-0.047	0.054	0.561	0.007	0.062	0.940	-0.205	0.119	0.766
Gal_9	LGALS9	Galectin-9	LVEF	-0.110	0.049	0.095	-0.146	0.059	0.055	0.043	0.098	0.977
HAOX1	HAO1	Hydroxyacid oxidase 1	LVEF	-0.041	0.058	0.662	-0.049	0.062	0.573	0.041	0.125	0.977
HB_EGF	HBEGF	Proheparin-binding EGF-like growth factor [Cleaved into: Heparin-binding EGF-like growth factor	LVEF	-0.059	0.055	0.453	-0.106	0.062	0.181	0.159	0.125	0.948
HO_1	HMOX1	Heme oxygenase 1	LVEF	-0.064	0.056	0.413	-0.102	0.061	0.188	0.123	0.120	0.972
HSP_27	HSPB1	Heat shock protein beta-1	LVEF	0.031	0.031	0.475	0.093	0.062	0.241	-0.136	0.124	0.948
IDUA	IDUA	Alpha-L-iduronidase	LVEF	-0.015	0.060	0.915	-0.052	0.061	0.544	0.141	0.126	0.948
IL16	IL16	Pro-interleukin-16 [Cleaved into: Interleukin-16	LVEF	-0.040	0.061	0.683	-0.057	0.062	0.506	0.090	0.118	0.977
IL18	IL18	Interleukin-18	LVEF	0.068	0.054	0.358	0.033	0.059	0.722	0.174	0.124	0.903
IL1RL2	IL1RL2	Interleukin-1 receptor-like 2	LVEF	0.083	0.053	0.268	0.032	0.060	0.729	0.262	0.108	0.595
IL6	IL6	Interleukin-6	LVEF	-0.105	0.056	0.162	-0.148	0.061	0.058	0.166	0.105	0.766
IL_17D	IL17D	Interleukin-17D	LVEF	-0.105	0.053	0.137	-0.117	0.060	0.126	-0.018	0.107	0.982
IL_1ra	IL1RN	Interleukin-1 receptor antagonist protein	LVEF	-0.078	0.054	0.303	-0.114	0.057	0.120	0.117	0.103	0.948
IL_27	IL27	Interleukin-27	LVEF	**-0.175**	**0.053**	**0.007**	**-0.213**	**0.060**	**0.003**	-0.019	0.117	0.982
IL_4RA	IL4R	Interleukin-4 receptor subunit alpha	LVEF	-0.008	0.055	0.952	-0.022	0.059	0.823	0.044	0.116	0.977
ITGB1BP2	ITGB1BP2	Integrin beta-1-binding protein 2	LVEF	0.019	0.045	0.811	0.031	0.062	0.748	-0.008	0.126	0.988
IgG_Fc_receptor_II_b	FCGR2B	Low affinity immunoglobulin gamma Fc region receptor II-b	LVEF	0.058	0.217	0.102	-0.069	0.062	0.400	-0.063	0.123	0.977
			LVEF'	0.018	0.052							
			LVEF''	-0.222	0.226							
KIM1	HAVCR1	Hepatitis A virus cellular receptor 1	LVEF	**-0.158**	**0.055**	**0.025**	-0.141	0.057	0.052	-0.169	0.105	0.766
LEP	LEP	Leptin	LVEF	**0.172**	**0.040**	**<0.001**	**0.201**	**0.047**	**<0.001**	0.063	0.092	0.977
LOX_1	OLR1	Oxidized low-density lipoprotein receptor 1	LVEF	-0.120	0.064	0.163	-0.139	0.062	0.080	0.018	0.127	0.982
LPL	LPL	Lipoprotein lipase	LVEF	-0.027	0.052	0.769	-0.007	0.062	0.940	-0.112	0.120	0.977
MARCO	MARCO	Macrophage receptor MARCO	LVEF	0.049	0.059	0.582	0.022	0.062	0.833	0.176	0.111	0.766
MERTK	MERTK	Tyrosine-protein kinase Mer	LVEF	-0.085	0.061	0.316	-0.080	0.061	0.310	-0.049	0.118	0.977
MMP12	MMP12	Macrophage metalloelastase	LVEF	**0.497**	**0.204**	**0.003**	**0.647**	**0.227**	**0.001**	-0.060	0.107	0.977
			LVEF'	**-0.122**	**0.049**		**-0.183**	**0.063**				
			LVEF''	**0.385**	**0.212**		**0.505**	**0.222**				
MMP7	MMP7	Matrilysin	LVEF	**-0.105**	**0.040**	**0.040**	**-0.162**	**0.060**	**0.032**	-0.080	0.111	0.977
NEMO	IKBKG	NF-kappa-B essential modulator	LVEF	-0.027	0.043	0.708	-0.035	0.062	0.722	-0.021	0.123	0.982
PAPPA	PAPPA	Pappalysin-1	LVEF	-0.078	0.066	0.387	-0.079	0.062	0.331	-0.011	0.118	0.988
PARP_1	PARP1	Poly [ADP-ribose] polymerase 1	LVEF	-0.125	0.062	0.137	-0.133	0.062	0.093	0.090	0.114	0.977
PAR_1	F2R	Proteinase-activated receptor 1	LVEF	-0.068	0.053	0.350	-0.083	0.061	0.286	0.017	0.119	0.982
PDGF_subunit_B	PDGFB	Platelet-derived growth factor subunit B	LVEF	-0.045	0.051	0.557	-0.070	0.062	0.396	0.033	0.126	0.977
PD_L2	PDCD1LG2	Programmed cell death 1 ligand 2	LVEF	-0.106	0.058	0.173	-0.116	0.061	0.135	0.008	0.112	0.988
PGF	PGF	Placenta growth factor	LVEF	-0.027	0.057	0.788	-0.027	0.061	0.787	-0.011	0.109	0.988
PIgR	PIGR	Polymeric immunoglobulin receptor	LVEF	-0.118	0.050	0.080	-0.096	0.059	0.198	-0.237	0.114	0.621
PRELP	PRELP	Prolargin	LVEF	**-0.255**	**0.055**	**<0.001**	**-0.255**	**0.058**	**<0.001**	-0.119	0.103	0.948
PRSS27	PRSS27	Serine protease 27	LVEF	0.067	0.057	0.388	0.090	0.063	0.263	-0.029	0.127	0.977
PRSS8	PRSS8	Prostasin	LVEF	-0.072	0.058	0.361	-0.092	0.061	0.239	0.025	0.124	0.982
PSGL_1	SELPLG	P-selectin glycoprotein ligand 1	LVEF	-0.026	0.059	0.809	-0.018	0.062	0.867	0.010	0.122	0.988
PTX3	PTX3	Pentraxin-related protein PTX3	LVEF	**-0.260**	**0.052**	**<0.001**	**-0.291**	**0.059**	**<0.001**	-0.133	0.125	0.952
RAGE	AGER	Advanced glycosylation end product-specific receptor	LVEF	**-0.214**	**0.063**	**0.006**	**-0.215**	**0.060**	**0.003**	0.022	0.125	0.982
REN	REN	Renin	LVEF	0.009	0.062	0.952	0.016	0.062	0.889	0.009	0.123	0.988
SCF	KITLG	Kit ligand	LVEF	**0.183**	**0.058**	**0.012**	**0.170**	**0.060**	**0.023**	0.201	0.118	0.766
SERPINA12	SERPINA12	Serpin A12	LVEF	0.212	0.221	0.097	0.079	0.239	0.130	-0.157	0.123	0.948
			LVEF'	-0.111	0.053		-0.098	0.066				
			LVEF''	0.565	0.229		0.440	0.233				
SLAMF7	SLAMF7	SLAM family member 7	LVEF	-0.082	0.056	0.293	-0.100	0.062	0.198	0.000	0.120	0.998
SOD2	SOD2	Superoxide dismutase [Mn], mitochondrial	LVEF	-0.114	0.064	0.188	-0.099	0.062	0.203	-0.050	0.126	0.977
SORT1	SORT1	Sortilin	LVEF	**-0.203**	**0.058**	**0.004**	**-0.231**	**0.061**	**0.002**	0.039	0.114	0.977
SPON2	SPON2	Spondin-2	LVEF	**-0.202**	**0.057**	**0.004**	**-0.222**	**0.060**	**0.002**	-0.007	0.121	0.988
SRC	SRC	Proto-oncogene tyrosine-protein kinase Src	LVEF	0.067	0.041	0.234	0.117	0.061	0.133	-0.035	0.125	0.977
STK4	STK4	Serine/threonine-protein kinase 4	LVEF	0.037	0.040	0.526	0.063	0.062	0.447	-0.010	0.126	0.988
TF	F3	Tissue factor	LVEF	0.021	0.057	0.848	0.035	0.059	0.712	-0.091	0.107	0.977
TGM2	TGM2	Protein-glutamine gamma-glutamyltransferase 2	LVEF	**-0.165**	**0.052**	**0.011**	**-0.220**	**0.061**	**0.003**	0.077	0.118	0.977
THBS2	THBS2	Thrombospondin-2	LVEF	**-0.241**	**0.056**	**<0.001**	**-0.267**	**0.060**	**<0.001**	-0.032	0.120	0.977
THPO	THPO	Thrombopoietin	LVEF	0.061	0.060	0.475	0.069	0.062	0.400	0.027	0.124	0.977
TIE2	TEK	Angiopoietin-1 receptor	LVEF	**-0.194**	**0.062**	**0.012**	**-0.194**	**0.061**	**0.009**	-0.013	0.120	0.988

TM	THBD	Thrombomodulin	LVEF	0.085	0.061	0.316	0.087	0.061	0.263	0.036	0.120	0.977
TNFRSF10A	TNFRSF10A	Tumor necrosis factor receptor superfamily member 10A	LVEF	-0.134	0.055	0.066	**-0.040**	**0.232**	**0.017**	0.065	0.110	0.977
			LVEF'				**0.023**	**0.064**				
			LVEF''				**-0.200**	**0.227**				
TNFRSF11A	TNFRSF11A	Tumor necrosis factor receptor superfamily member 11A	LVEF	-0.081	0.059	0.322	-0.108	0.058	0.139	0.112	0.108	0.972
TNFRSF13B	TNFRSF13B	Tumor necrosis factor receptor superfamily member 13B	LVEF	**-0.219**	**0.055**	**0.001**	**-0.244**	**0.060**	**0.001**	-0.076	0.121	0.977
TRAIL_R2	TNFRSF10B	Tumor necrosis factor receptor superfamily member 10B	LVEF	**-0.253**	**0.056**	**<0.001**	**-0.266**	**0.057**	**<0.001**	0.004	0.098	0.989
VEGFD	VEGFD	Vascular endothelial growth factor D	LVEF	**-0.320**	**0.049**	**<0.001**	**-0.367**	**0.056**	**<0.001**	-0.156	0.124	0.948
VSIG2	VSIG2	V-set and immunoglobulin domain-containing protein 2	LVEF	0.008	0.060	0.952	-0.008	0.059	0.935	0.031	0.116	0.977
XCL1	XCL1	Lymphotactin	LVEF	-0.126	0.050	0.052	**-0.211**	**0.061**	**0.004**	0.276	0.119	0.595
hOSCAR	OSCAR	Osteoclast-associated immunoglobulin-like receptor	LVEF	-0.039	0.062	0.697	-0.033	0.062	0.727	-0.036	0.123	0.977
**CVD3 Panel**												
ALCAM	ALCAM	CD166 antigen	LVEF	-0.156	0.061	0.052	**-0.175**	**0.061**	**0.020**	0.049	0.119	0.977
AP_N	ANPEP	Aminopeptidase N	LVEF	-0.111	0.061	0.176	-0.110	0.060	0.146	0.043	0.122	0.977
AXL	AXL	Tyrosine-protein kinase receptor UFO	LVEF	-0.078	0.060	0.349	-0.102	0.060	0.181	1.073	0.440	0.766
			LVEF'							-0.252	0.149	
			LVEF''							0.596	0.454	
AZU1	AZU1	Azurocidin	LVEF	0.003	0.051	0.986	-0.002	0.062	0.978	0.046	0.126	0.977
BLM_hydrolase	BLMH	Bleomycin hydrolase	LVEF	**-0.160**	**0.060**	**0.039**	-0.151	0.061	0.051	-0.072	0.117	0.977
CASP_3	CASP3	Caspase-3	LVEF	0.010	0.050	0.940	0.025	0.062	0.806	-0.037	0.125	0.977
CCL15	CCL15	C-C motif chemokine 15	LVEF	**-0.226**	**0.053**	**<0.001**	**-0.258**	**0.058**	**<0.001**	-0.041	0.121	0.977
CCL16	CCL16	C-C motif chemokine 16	LVEF	-0.111	0.053	0.113	-0.127	0.061	0.107	-0.043	0.121	0.977
CCL24	CCL24	C-C motif chemokine 24	LVEF	0.145	0.220	0.105	0.133	0.234	0.102	0.052	0.127	0.977
			LVEF'	0.010	0.053		0.012	0.065				
			LVEF''	-0.205	0.229		-0.181	0.230				
CD163	CD163	Scavenger receptor cysteine-rich type 1 protein M130	LVEF	-0.135	0.056	0.066	**-0.166**	**0.060**	**0.027**	0.047	0.112	0.977
CD93	CD93	Complement component C1q receptor	LVEF	**-0.184**	**0.063**	**0.021**	**-0.178**	**0.059**	**0.014**	0.008	0.122	0.988
CDH5	CDH5	Cadherin-5	LVEF	-0.114	0.057	0.137	-0.139	0.061	0.080	0.053	0.123	0.977
CHI3L1	CHI3L1	Chitinase-3-like protein 1	LVEF	-0.077	0.052	0.293	-0.116	0.060	0.129	0.160	0.100	0.766
CHIT1	CHIT1	Chitotriosidase-1	LVEF	0.575	0.206	0.137	0.723	0.229	0.069	0.036	0.127	0.977
			LVEF'	-0.115	0.049		-0.178	0.064				
			LVEF''	0.415	0.215		0.552	0.225				
CNTN1	CNTN1	Contactin-1	LVEF	-0.079	0.058	0.324	-0.084	0.062	0.287	0.016	0.120	0.984
COL1A1	COL1A1	Collagen alpha-1	LVEF	-0.080	0.059	0.324	-0.097	0.059	0.195	0.126	0.124	0.972
CPA1	CPA1	Carboxypeptidase A1	LVEF	-0.041	0.056	0.642	-0.029	0.061	0.764	-0.104	0.125	0.977
CPB1	CPB1	Carboxypeptidase B	LVEF	-0.088	0.057	0.269	-0.055	0.061	0.513	-0.213	0.119	0.742
CSTB	CSTB	Cystatin-B	LVEF	-0.113	0.060	0.164	-0.121	0.059	0.112	0.051	0.106	0.977
CTSD	CTSD	Cathepsin D	LVEF	**-0.160**	**0.055**	**0.021**	**-0.200**	**0.060**	**0.006**	0.087	0.103	0.977
CTSZ	CTSZ	Cathepsin Z	LVEF	0.080	0.055	0.296	0.046	0.059	0.582	0.530	0.445	0.595
			LVEF'							0.046	0.150	
			LVEF''							-0.362	0.459	
CXCL16	CXCL16	C-X-C motif chemokine 16	LVEF	-0.115	0.061	0.162	-0.124	0.062	0.118	0.588	0.479	0.788
			LVEF'							-0.057	0.162	
			LVEF''							-0.066	0.494	
DLK_1	DLK1	Protein delta homolog 1	LVEF	0.110	0.053	0.119	0.131	0.060	0.089	0.037	0.118	0.977
EGFR	EGFR	Epidermal growth factor receptor	LVEF	0.501	0.212	0.102	0.493	0.232	0.130	-0.063	0.114	0.977
			LVEF'	-0.079	0.051		-0.079	0.065				
			LVEF''	0.195	0.221		0.156	0.228				
EPHB4	EPHB4	Ephrin type-B receptor 4	LVEF	-0.066	0.060	0.433	-0.065	0.058	0.400	1.244	0.468	0.690
			LVEF'							-0.367	0.158	
			LVEF''							0.946	0.482	
Ep_CAM	EPCAM	Epithelial cell adhesion molecule	LVEF	-0.002	0.053	0.987	0.034	0.062	0.722	-0.150	0.124	0.948
FABP4	FABP4	Fatty acid-binding protein, adipocyte	LVEF	**-0.145**	**0.054**	**0.037**	**-0.173**	**0.057**	**0.013**	0.030	0.100	0.977
FAS	FAS	Tumor necrosis factor receptor superfamily member 6	LVEF	-0.014	0.055	0.914	-0.055	0.060	0.511	0.162	0.116	0.903
GDF_15	GDF15	Growth/differentiation factor 15	LVEF	**-0.247**	**0.050**	**<0.001**	**-0.279**	**0.053**	**<0.001**	1.085	0.404	0.690
			LVEF'							-0.313	0.136	
			LVEF''							0.803	0.417	
GP6	GP6	Platelet glycoprotein VI	LVEF	0.021	0.054	0.842	0.014	0.062	0.897	0.071	0.125	0.977
GRN	GRN	Granulins	LVEF	**-0.188**	**0.056**	**0.007**	**-0.203**	**0.060**	**0.006**	0.006	0.118	0.989
Gal_3	LGALS3	Galectin-3	LVEF	-0.044	0.060	0.641	-0.025	0.062	0.808	-0.079	0.119	0.977
Gal_4	LGALS4	Galectin-4	LVEF	-0.020	0.054	0.844	-0.023	0.060	0.816	-0.005	0.116	0.989
ICAM_2	ICAM2	Intercellular adhesion molecule 2	LVEF	-0.089	0.056	0.253	-0.098	0.061	0.203	0.010	0.120	0.988
IGFBP_1	IGFBP1	Insulin-like growth factor-binding protein 1	LVEF	**-0.245**	**0.046**	**<0.001**	**-0.291**	**0.051**	**<0.001**	-0.067	0.119	0.977
IGFBP_2	IGFBP2	Insulin-like growth factor-binding protein 2	LVEF	**-0.179**	**0.049**	**0.003**	**-0.219**	**0.052**	**<0.001**	0.099	0.118	0.977
IGFBP_7	IGFBP7	Insulin-like growth factor-binding protein 7	LVEF	**-0.268**	**0.060**	**<0.001**	**-0.280**	**0.058**	**<0.001**	0.037	0.118	0.977
IL2_RA	IL2RA	Interleukin-2 receptor subunit alpha	LVEF	-0.010	0.058	0.943	-0.038	0.062	0.686	1.090	0.472	0.716
			LVEF'							-0.216	0.159	
			LVEF''							0.434	0.486	
IL_17RA	IL17RA	Interleukin-17 receptor A	LVEF	-0.071	0.056	0.358	-0.072	0.062	0.379	-0.027	0.127	0.977
IL_18BP	IL18BP	Interleukin-18-binding protein	LVEF	-0.025	0.058	0.810	-0.039	0.059	0.655	0.089	0.117	0.977
IL_1RT1	IL1R1	Interleukin-1 receptor type 1	LVEF	-0.135	0.060	0.094	-0.133	0.059	0.080	-0.039	0.117	0.977
IL_1RT2	IL1R2	Interleukin-1 receptor type 2	LVEF	0.006	0.059	0.962	-0.002	0.062	0.978	0.069	0.125	0.977
IL_6RA	IL6R	Interleukin-6 receptor subunit alpha	LVEF	-0.073	0.057	0.358	-0.103	0.062	0.189	0.102	0.120	0.977
ITGB2	ITGB2	Integrin beta-2	LVEF	0.025	0.052	0.794	0.009	0.061	0.929	0.134	0.114	0.948
JAM_A	F11R	Junctional adhesion molecule A	LVEF	-0.134	0.061	0.102	-0.111	0.061	0.149	-0.129	0.119	0.948
KLK6	KLK6	Kallikrein-6	LVEF	0.008	0.058	0.952	0.032	0.061	0.729	-0.106	0.121	0.977
LDL_receptor	LDLR	Low-density lipoprotein receptor	LVEF	0.135	0.056	0.067	**0.161**	**0.059**	**0.028**	0.033	0.119	0.977
LTBR	LTBR	Tumor necrosis factor receptor superfamily member 3	LVEF	-0.097	0.060	0.246	-0.107	0.058	0.143	0.063	0.120	0.977
MB	MB	Myoglobin	LVEF	0.034	0.055	0.708	0.032	0.061	0.735	0.022	0.126	0.982
MCP_1	CCL2	C-C motif chemokine 2	LVEF	-0.085	0.065	0.350	-0.089	0.062	0.263	0.088	0.109	0.977
MEPE	MEPE	Matrix extracellular phosphoglycoprotein	LVEF	0.019	0.053	0.848	0.014	0.060	0.896	0.100	0.124	0.977
MMP_2	MMP2	72 kDa type IV collagenase	LVEF	**-0.348**	**0.059**	**<0.001**	**-0.373**	**0.056**	**<0.001**	0.094	0.121	0.977
MMP_3	MMP3	Stromelysin-1	LVEF	-0.058	0.052	0.437	-0.073	0.059	0.346	0.080	0.125	0.977
MMP_9	MMP9	Matrix metalloproteinase-9	LVEF	-0.059	0.060	0.495	-0.440	0.235	0.103	0.097	0.123	0.977
			LVEF'				0.047	0.065				
			LVEF''				-0.042	0.231				
MPO	MPO	Myeloperoxidase	LVEF	0.094	0.056	0.227	0.086	0.062	0.276	0.129	0.124	0.972
NT_proBNP	NT_proBNP	Natriuretic peptides B	LVEF	**-0.359**	**0.036**	**<0.001**	**-0.489**	**0.051**	**<0.001**	-0.281	0.112	0.595
Notch_3	NOTCH3	Neurogenic locus notch homolog protein 3	LVEF	**-0.280**	**0.059**	**<0.001**	**-0.304**	**0.056**	**<0.001**	0.097	0.110	0.977
OPG	TNFRSF11B	Tumor necrosis factor receptor superfamily member 11B	LVEF	-0.137	0.060	0.090	-0.121	0.057	0.098	-0.109	0.121	0.977
OPN	SPP1	Osteopontin	LVEF	-0.131	0.054	0.066	**-0.170**	**0.059**	**0.019**	0.112	0.118	0.977
PAI	SERPINE1	Plasminogen activator inhibitor 1	LVEF	-0.073	0.054	0.327	-0.116	0.060	0.132	0.122	0.123	0.972
PCSK9	PCSK9	Proprotein convertase subtilisin/kexin type 9	LVEF	-0.035	0.054	0.690	-0.055	0.061	0.511	0.145	0.118	0.948
PDGF_subunit_A	PDGFA	Platelet-derived growth factor subunit A	LVEF	-0.093	0.055	0.227	-0.119	0.062	0.130	0.035	0.125	0.977
PECAM_1	PECAM1	Platelet endothelial cell adhesion molecule	LVEF	-0.092	0.066	0.316	-0.091	0.062	0.253	0.020	0.123	0.982

PGLYRP1	PGLYRP1	Peptidoglycan recognition protein 1	LVEF	-0.078	0.056	0.316	-0.127	0.061	0.102	0.161	0.123	0.948
PI3	PI3	Elafin	LVEF	0.004	0.054	0.977	-0.007	0.061	0.942	0.083	0.125	0.977
PLC	HSPG2	Basement membrane-specific heparan sulfate proteoglycan core protein	LVEF	**-0.186**	**0.060**	**0.012**	**-0.197**	**0.059**	**0.006**	0.043	0.115	0.977
PON3	PON3	Serum paraoxonase/lactonase 3	LVEF	0.125	0.050	0.060	**0.166**	**0.061**	**0.028**	0.004	0.123	0.989
PRTN3	PRTN3	Myeloblastin	LVEF	-0.077	0.060	0.357	-0.097	0.062	0.213	0.059	0.125	0.977
PSP_D	SFTPD	Pulmonary surfactant-associated protein D	LVEF	**-0.149**	**0.055**	**0.037**	**-0.193**	**0.060**	**0.008**	0.066	0.126	0.977
RARRES2	RARRES2	Retinoic acid receptor responder protein 2	LVEF	-0.090	0.053	0.214	-0.099	0.061	0.198	-0.043	0.120	0.977
RETN	RETN	Resistin	LVEF	-0.117	0.057	0.123	**-0.159**	**0.059**	**0.032**	0.090	0.126	0.977
SCGB3A2	SCGB3A2	Secretoglobin family 3A member 2	LVEF	-0.088	0.055	0.251	-0.097	0.061	0.212	-0.027	0.125	0.977
SELE	SELE	E-selectin	LVEF	0.044	0.056	0.620	0.024	0.060	0.811	0.124	0.126	0.972
SELP	SELP	P-selectin	LVEF	-0.047	0.062	0.632	-0.063	0.062	0.447	0.065	0.125	0.977
SHPS_1	SIRPA	Tyrosine-protein phosphatase non-receptor type substrate 1	LVEF	-0.096	0.057	0.226	-0.105	0.060	0.171	-0.031	0.123	0.977
SPON1	SPON1	Spondin-1	LVEF	-0.035	0.065	0.760	-0.024	0.062	0.811	-0.030	0.124	0.977
ST2	IL1RL1	Interleukin-1 receptor-like 1	LVEF	**-0.232**	**0.058**	**0.001**	**-0.251**	**0.059**	**<0.001**	0.023	0.121	0.982
TFF3	TFF3	Trefoil factor 3	LVEF	**-0.157**	**0.056**	**0.027**	**-0.173**	**0.055**	**0.009**	-0.197	0.120	0.766
TFPI	TFPI	Tissue factor pathway inhibitor	LVEF	0.049	0.061	0.606	0.048	0.061	0.577	0.076	0.125	0.977
TIMP4	TIMP4	Metalloproteinase inhibitor 4	LVEF	0.001	0.053	0.988	-0.031	0.057	0.727	0.188	0.114	0.766
TLT_2	TREML2	Trem-like transcript 2 protein	LVEF	0.003	0.059	0.986	-0.034	0.061	0.722	0.150	0.122	0.948
TNFRSF10C	TNFRSF10C	Tumor necrosis factor receptor superfamily member 10C	LVEF	-0.026	0.056	0.794	-0.056	0.060	0.506	0.064	0.122	0.977
TNFRSF14	TNFRSF14	Tumor necrosis factor receptor superfamily member 14	LVEF	-0.119	0.060	0.137	-0.119	0.058	0.112	0.003	0.118	0.991
TNFSF13B	TNFSF13B	Tumor necrosis factor ligand superfamily member 13B	LVEF	**-0.194**	**0.060**	**0.010**	**-0.196**	**0.060**	**0.007**	-0.030	0.122	0.977
TNF_R1	TNFRSF1A	Tumor necrosis factor receptor superfamily member 1A	LVEF	-0.081	0.057	0.304	-0.097	0.056	0.178	0.078	0.114	0.977
TNF_R2	TNFRSF1B	Tumor necrosis factor receptor superfamily member 1B	LVEF	-0.051	0.058	0.562	-0.063	0.057	0.404	0.070	0.115	0.977
TR_AP	ACP5	Tartrate-resistant acid phosphatase type 5	LVEF	**0.790**	**0.238**	**0.003**	**0.204**	**0.061**	**0.006**	0.049	0.120	0.977
			LVEF'	**-0.107**	**0.057**							
			LVEF''	**0.313**	**0.249**							
TR	TFRC	Transferrin receptor protein 1	LVEF	**-0.230**	**0.060**	**0.002**	**-0.257**	**0.059**	**<0.001**	0.071	0.122	0.977
U_PAR	PLAUR	Urokinase plasminogen activator surface receptor	LVEF	**-0.192**	**0.054**	**0.004**	**-0.223**	**0.058**	**0.001**	0.028	0.115	0.977
t_PA	PLAT	Tissue-type plasminogen activator	LVEF	-0.140	0.056	0.057	**-0.177**	**0.061**	**0.017**	0.085	0.107	0.977
uPA	PLAU	Urokinase-type plasminogen activator	LVEF	-0.135	0.060	0.092	**0.306**	**0.234**	**0.008**	0.143	0.118	0.948
			LVEF'				**-0.103**	**0.065**				
			LVEF''				**0.255**	**0.230**				
vWF	VWF	von Willebrand factor	LVEF	**-0.182**	**0.054**	**0.006**	**-0.219**	**0.058**	**0.002**	0.027	0.120	0.977
**Inflammation Panel**												
ADA	ADA	Adenosine deaminase	LVEF	-0.010	0.065	0.952	-0.012	0.062	0.909	0.032	0.123	0.977
ARTN	ARTN	Artemin	LVEF	0.039	0.061	0.697	0.587	0.237	0.181	0.083	0.116	0.977
			LVEF'				-0.137	0.066				
			LVEF''				0.400	0.232				
AXIN1	AXIN1	Axin-1	LVEF	0.002	0.048	0.986	0.004	0.062	0.966	0.000	0.125	0.998
Beta_NGF	NGF	Beta-nerve growth factor	LVEF	-0.069	0.046	0.281	-0.114	0.062	0.143	0.062	0.127	0.977
CASP_8	CASP8	Caspase-8	LVEF	-0.094	0.059	0.253	-0.104	0.062	0.188	0.013	0.122	0.988
CCL3	CCL3	C-C motif chemokine 3	LVEF	**-0.145**	**0.053**	**0.033**	**-0.193**	**0.060**	**0.008**	0.096	0.110	0.977
CCL11	CCL11	Eotaxin	LVEF	-0.021	0.054	0.838	-0.061	0.233	0.107	0.245	0.104	0.595
			LVEF'				0.057	0.065				
			LVEF''				-0.320	0.228				
CCL19	CCL19	C-C motif chemokine 19	LVEF	-0.082	0.054	0.280	-0.094	0.062	0.230	-0.009	0.124	0.988
CCL20	CCL20	C-C motif chemokine 20	LVEF	-0.094	0.058	0.246	-0.124	0.063	0.120	0.107	0.117	0.977
CCL23	CCL23	C-C motif chemokine 23	LVEF	-0.109	0.051	0.104	-0.114	0.061	0.139	-0.059	0.120	0.977
CCL25	CCL25	C-C motif chemokine 25	LVEF	-0.039	0.057	0.668	-0.011	0.061	0.910	-0.133	0.111	0.948
CCL28	CCL28	C-C motif chemokine 28	LVEF	0.070	0.066	0.456	0.068	0.062	0.413	0.017	0.121	0.983
CCL4	CCL4	C-C motif chemokine 4	LVEF	-0.018	0.057	0.881	-0.073	0.062	0.376	0.312	0.109	0.595
CD244	CD244	Natural killer cell receptor 2B4	LVEF	-0.018	0.060	0.889	-0.067	0.062	0.418	0.251	0.112	0.595
CD40	CD40	Tumor necrosis factor receptor superfamily member 5	LVEF	-0.070	0.058	0.387	-0.131	0.060	0.088	0.275	0.105	0.595
CD5	CD5	T-cell surface glycoprotein CD5	LVEF	-0.003	0.060	0.986	-0.055	0.062	0.518	0.245	0.116	0.621
CD6	CD6	T-cell differentiation antigen CD6	LVEF	-0.001	0.061	0.988	-0.022	0.063	0.833	0.141	0.117	0.948
CD8A	CD8A	T-cell surface glycoprotein CD8 alpha chain	LVEF	-0.103	0.054	0.162	-0.134	0.062	0.090	-0.780	0.451	0.595
			LVEF'							0.122	0.152	
			LVEF''							-0.088	0.465	
CDCP1	CDCP1	CUB domain-containing protein 1	LVEF	-0.036	0.051	0.662	-0.086	0.059	0.254	0.185	0.100	0.742
CSF_1	CSF1	Macrophage colony-stimulating factor 1	LVEF	**-0.160**	**0.058**	**0.032**	**-0.202**	**0.059**	**0.005**	0.099	0.119	0.977
CST5	CST5	Cystatin-D	LVEF	-0.078	0.054	0.303	-0.125	0.060	0.105	0.181	0.107	0.766
CX3CL1	CX3CL1	Fractalkine	LVEF	-0.104	0.057	0.173	-0.140	0.060	0.069	0.101	0.122	0.977
CXCL10	CXCL10	C-X-C motif chemokine 10	LVEF	-0.135	0.058	0.079	**-0.159**	**0.061**	**0.037**	0.063	0.112	0.977
CXCL11	CXCL11	C-X-C motif chemokine 11	LVEF	**-0.154**	**0.054**	**0.025**	**-0.213**	**0.061**	**0.004**	0.137	0.119	0.948
CXCL1	CXCL1	Growth-regulated alpha protein	LVEF	-0.005	0.047	0.962	-0.029	0.063	0.767	0.110	0.126	0.977
CXCL5	CXCL5	C-X-C motif chemokine 5	LVEF	0.028	0.053	0.761	0.014	0.062	0.896	0.099	0.126	0.977
CXCL6	CXCL6	C-X-C motif chemokine 6	LVEF	-0.040	0.054	0.637	-0.091	0.062	0.254	0.173	0.124	0.903
CXCL9	CXCL9	C-X-C motif chemokine 9	LVEF	-0.011	0.054	0.940	-0.004	0.062	0.966	0.004	0.113	0.989
DNER	DNER	Delta and Notch-like epidermal growth factor-related receptor	LVEF	0.118	0.058	0.126	0.515	0.229	0.080	0.151	0.122	0.948
			LVEF'				-0.071	0.063				
			LVEF''				0.138	0.223				
EN_RAGE	S100A12	Protein S100-A12	LVEF	-0.102	0.066	0.269	-0.090	0.062	0.255	-0.061	0.124	0.977
FGF_19	FGF19	Fibroblast growth factor 19	LVEF	-0.024	0.053	0.800	-0.034	0.063	0.727	-0.042	0.118	0.977
FGF_21	FGF21	Fibroblast growth factor 21	LVEF	**-0.182**	**0.055**	**0.008**	**-0.233**	**0.060**	**0.001**	0.042	0.125	0.977
FGF_23	FGF23	Fibroblast growth factor 23	LVEF	**-0.358**	**0.066**	**<0.001**	**-0.326**	**0.059**	**<0.001**	-0.064	0.124	0.977
FGF_5	FGF5	Fibroblast growth factor 5	LVEF	0.036	0.065	0.760	0.053	0.063	0.544	-0.051	0.112	0.977
Flt3L	FLT3LG	Fms-related tyrosine kinase 3 ligand	LVEF	0.016	0.055	0.889	-0.010	0.062	0.925	0.131	0.121	0.948
GDNF	GDNF	Glial cell line-derived neurotrophic factor	LVEF	-0.083	0.060	0.324	-0.116	0.062	0.138	0.107	0.122	0.977
HGF	HGF	Hepatocyte growth factor	LVEF	0.014	0.047	0.889	-0.009	0.063	0.929	0.137	0.120	0.948
IFN_gamma	IFNG	Interferon gamma	LVEF	-0.090	0.060	0.285	-0.126	0.062	0.113	0.135	0.122	0.948
IL10	IL10	Interleukin-10	LVEF	0.017	0.048	0.848	-0.044	0.062	0.620	0.190	0.120	0.766
IL13	IL13	Interleukin-13	LVEF	-0.032	0.067	0.788	-0.004	0.063	0.966	-0.083	0.126	0.977
IL18	IL18	Interleukin-18	LVEF	0.090	0.054	0.227	0.547	0.228	0.139	0.222	0.124	0.742
			LVEF'				-0.163	0.063				
			LVEF''				0.578	0.223				
IL2	IL2	Interleukin-2	LVEF	-0.011	0.060	0.943	-0.028	0.063	0.778	0.060	0.126	0.977
IL33	IL33	Interleukin-33	LVEF	-0.009	0.049	0.943	-0.047	0.063	0.592	0.104	0.124	0.977
IL4	IL4	Interleukin-4	LVEF	-0.011	0.059	0.942	-0.018	0.063	0.870	0.035	0.122	0.977
IL5	IL5	Interleukin-5	LVEF	-0.008	0.048	0.943	-0.039	0.063	0.679	0.094	0.126	0.977
IL6	IL6	Interleukin-6	LVEF	-0.092	0.057	0.249	-0.141	0.062	0.078	0.219	0.106	0.621
IL7	IL7	Interleukin-7	LVEF	-0.034	0.055	0.708	-0.076	0.063	0.356	0.177	0.125	0.903
IL8	CXCL8	Interleukin-8	LVEF	**-0.198**	**0.054**	**0.003**	**-0.276**	**0.060**	**<0.001**	0.120	0.120	0.972
IL_10RA	IL10RA	Interleukin-10 receptor subunit alpha	LVEF	-0.040	0.054	0.637	-0.012	0.063	0.909	-0.163	0.124	0.948
IL_10RB	IL10RB	Interleukin-10 receptor subunit beta	LVEF	0.077	0.056	0.324	0.049	0.060	0.563	0.157	0.120	0.948
IL_12B	IL12B	Interleukin-12 subunit beta	LVEF	-0.062	0.058	0.445	-0.084	0.062	0.288	0.080	0.119	0.977

IL_15RA	IL15RA	Interleukin-15 receptor subunit alpha	LVEF	-0.068	0.057	0.388	-0.103	0.060	0.178	0.134	0.118	0.948
IL_17A	IL17A	Interleukin-17A	LVEF	0.002	0.061	0.987	-0.048	0.063	0.588	0.202	0.123	0.766
IL_17C	IL17C	Interleukin-17C	LVEF	-0.060	0.055	0.445	-0.083	0.062	0.288	0.071	0.120	0.977
IL_18R1	IL18R1	Interleukin-18 receptor 1	LVEF	-0.029	0.056	0.777	-0.088	0.062	0.263	0.258	0.116	0.595
IL_1_alpha	IL1A	Interleukin-1 alpha	LVEF	0.074	0.053	0.316	0.079	0.063	0.331	0.068	0.124	0.977
IL_20RA	IL20RA	Interleukin-20 receptor subunit alpha	LVEF	-0.094	0.061	0.268	-0.106	0.063	0.181	0.039	0.126	0.977
IL_20	IL20	Interleukin-20	LVEF	-0.066	0.057	0.409	-0.085	0.063	0.288	0.069	0.126	0.977
IL_22_RA1	IL22RA1	Interleukin-22 receptor subunit alpha-1	LVEF	-0.030	0.055	0.760	-0.058	0.063	0.506	0.049	0.126	0.977
IL_24	IL24	Interleukin-24	LVEF	0.065	0.057	0.409	0.049	0.063	0.582	0.095	0.128	0.977
IL_2RB	IL2RB	Interleukin-2 receptor subunit beta	LVEF	-0.007	0.059	0.962	0.019	0.063	0.857	-0.101	0.126	0.977
LAP_TGF_beta_1	TGFB1	Transforming growth factor beta-1 proprotein	LVEF	-0.123	0.057	0.102	**-0.205**	**0.062**	**0.006**	0.250	0.122	0.621
LIF	LIF	Leukemia inhibitory factor	LVEF	-0.014	0.066	0.938	-0.010	0.063	0.925	-0.071	0.125	0.977
LIF_R	LIFR	Leukemia inhibitory factor receptor	LVEF	**-0.189**	**0.060**	**0.011**	**-0.236**	**0.060**	**0.001**	0.169	0.113	0.836
MCP_1	CCL2	C-C motif chemokine 2	LVEF	-0.070	0.058	0.386	-0.117	0.062	0.138	0.170	0.106	0.766
MCP_2	CCL8	C-C motif chemokine 8	LVEF	-0.007	0.057	0.952	-0.030	0.063	0.764	0.147	0.120	0.948
MCP_3	CCL7	C-C motif chemokine 7	LVEF	-0.033	0.053	0.700	-0.084	0.061	0.282	0.197	0.109	0.742
MCP_4	CCL13	C-C motif chemokine 13	LVEF	0.014	0.057	0.916	-0.016	0.062	0.889	0.172	0.114	0.836
MMP_10	MMP10	Stromelysin-2	LVEF	-0.044	0.056	0.610	-0.092	0.060	0.231	0.151	0.123	0.948
MMP_1	MMP1	Interstitial collagenase	LVEF	-0.116	0.056	0.124	-0.150	0.061	0.054	0.107	0.125	0.977
NRTN	NRTN	Neurturin	LVEF	0.072	0.055	0.350	0.062	0.063	0.469	0.122	0.125	0.977
NT_3	NTF3	Neurotrophin-3	LVEF	**-0.317**	**0.059**	**<0.001**	**-0.326**	**0.059**	**<0.001**	-0.056	0.127	0.977
OPG	TNFRSF11B	Tumor necrosis factor receptor superfamily member 11B	LVEF	-0.117	0.062	0.161	-0.115	0.058	0.120	-0.045	0.115	0.977
OSM	OSM	Oncostatin-M	LVEF	-0.441	0.227	0.173	-0.381	0.236	0.098	0.074	0.125	0.977
			LVEF'	0.056	0.054		0.027	0.065				
			LVEF''	-0.099	0.236		0.041	0.231				
PD_L1	CD274	Programmed cell death 1 ligand 1	LVEF	-0.055	0.058	0.503	-0.111	0.061	0.146	0.259	0.114	0.595
SCF	KITLG	Kit ligand	LVEF	**0.203**	**0.059**	**0.005**	**0.182**	**0.060**	**0.014**	0.243	0.117	0.621
SIRT2	SIRT2	NAD-dependent protein deacetylase sirtuin-2	LVEF	-0.006	0.057	0.962	-0.014	0.063	0.896	0.040	0.126	0.977
SLAMF1	SLAMF1	Signaling lymphocytic activation molecule	LVEF	-0.056	0.058	0.503	-0.081	0.062	0.310	0.095	0.123	0.977
ST1A1	SULT1A1	Sulfotransferase 1A1	LVEF	-0.008	0.045	0.943	-0.002	0.062	0.978	-0.036	0.126	0.977
STAMBP	STAMBP	STAM-binding protein	LVEF	0.000	0.061	0.998	-0.001	0.063	0.982	0.018	0.124	0.982
TGF_alpha	TGFA	Protransforming growth factor alpha	LVEF	-0.090	0.057	0.253	-0.138	0.061	0.080	0.171	0.122	0.903
TNFB	LTA	Lymphotoxin-alpha	LVEF	-0.088	0.058	0.269	-0.135	0.060	0.082	0.130	0.115	0.948
TNFRSF9	TNFRSF9	Tumor necrosis factor receptor superfamily member 9	LVEF	-0.080	0.055	0.298	-0.130	0.060	0.089	0.133	0.120	0.948
TNFSF14	TNFSF14	Tumor necrosis factor ligand superfamily member 14	LVEF	-0.072	0.058	0.358	-0.120	0.062	0.128	0.199	0.120	0.766
TNF	TNF	Tumor necrosis factor	LVEF	-0.100	0.053	0.162	**-0.182**	**0.060**	**0.014**	0.227	0.113	0.647
TRAIL	TNFSF10	Tumor necrosis factor ligand superfamily member 10	LVEF	0.151	0.060	0.055	0.118	0.061	0.128	0.291	0.114	0.595
TRANCE	TNFSF11	Tumor necrosis factor ligand superfamily member 11	LVEF	**0.184**	**0.055**	**0.007**	**0.160**	**0.055**	**0.019**	0.251	0.118	0.621
TSLP	TSLP	Thymic stromal lymphopoietin	LVEF	0.055	0.058	0.503	0.015	0.063	0.896	0.230	0.124	0.742
TWEAK	TNFSF12	Tumor necrosis factor ligand superfamily member 12	LVEF	0.110	0.064	0.210	0.110	0.062	0.162	0.056	0.122	0.977
uPA	PLAU	Urokinase-type plasminogen activator	LVEF	-0.125	0.060	0.119	**-0.189**	**0.061**	**0.012**	0.237	0.107	0.595
VEGFA	VEGFA	Vascular endothelial growth factor A	LVEF	-0.121	0.061	0.137	-0.153	0.061	0.051	0.154	0.122	0.948
4E_BP1	EIF4EBP1	Eukaryotic translation initiation factor 4E-binding protein 1	LVEF	-0.074	0.055	0.335	-0.105	0.062	0.181	0.103	0.117	0.977

Supplemental Table S5. Associations of left ventricular end diastolic diameter (LVEDD$^{\text{acc. to HENRY}}$) and the plasma proteins measured using Olink (targeted approach). Results from regression models adjusted for sex, age and BMI for the whole study population and from sex-specific models adjusted for age and BMI. Proteins were log2-transformed. Estimates are presented for z-transformed outcome and exposure variables.
stderr, standard error; FDR, false discovery rate

Outcome			Exposure	All subjects			Men			Women		
				beta	stderr	FDR	beta	stderr	FDR	beta	stderr	FDR
OLINK Label	Gene name	Protein name										
**CVD2 Panel**												
ACE2	ACE2	Angiotensin-converting enzyme 2	LVEDD acc. to HENRY	0.005	0.053	0.974	-0.006	0.062	0.966	0.057	0.125	0.954
ADAM_TS13	ADAMTS13	A disintegrin and metalloproteinase with thrombospondin motifs 13	LVEDD acc. to HENRY	0.029	0.055	0.828	-0.003	0.062	0.967	0.200	0.122	0.786
ADM	ADM	ADM [Cleaved into: Adrenomedullin]	LVEDD acc. to HENRY	0.078	0.053	0.515	0.073	0.062	0.641	0.119	0.124	0.938
AGRP	AGRP	Agouti-related protein	LVEDD acc. to HENRY	0.083	0.050	0.455	0.088	0.061	0.521	0.108	0.124	0.944
AMBP	AMBP	Protein AMBP [Cleaved into: Alpha-1-microglobulin]	LVEDD acc. to HENRY	-0.074	0.063	0.646	-0.105	0.062	0.506	0.134	0.123	0.872
ANGPT1	ANGPT1	Angiopoietin-1	LVEDD acc. to HENRY	-0.076	0.051	0.515	-0.287	0.280	0.404	-0.180	0.123	0.810
			LVEDD acc. to HENRY'				0.151	0.091				
			LVEDD acc. to HENRY''				-0.545	0.274				
BMP_6	BMP6	Bone morphogenetic protein 6	LVEDD acc. to HENRY	-0.012	0.043	0.910	-0.029	0.062	0.849	0.032	0.123	0.961
BNP	NPPB	Natriuretic peptides B	LVEDD acc. to HENRY	**0.186**	**0.040**	**0.002**	**0.235**	**0.060**	**0.014**	0.294	0.120	0.702
BOC	BOC	Brother of CDO	LVEDD acc. to HENRY	0.149	0.058	0.285	0.186	0.061	0.135	-0.083	0.124	0.944
CA5A	CA5A	Carbonic anhydrase 5A, mitochondrial	LVEDD acc. to HENRY	-0.076	0.059	0.610	-0.088	0.062	0.521	0.003	0.125	0.996
CCL17	CCL17	C-C motif chemokine 17	LVEDD acc. to HENRY	-0.060	0.054	0.672	-0.052	0.062	0.769	-0.101	0.124	0.944
CCL3	CCL3	C-C motif chemokine 3	LVEDD acc. to HENRY	0.034	0.056	0.819	0.069	0.062	0.678	1.319	0.550	0.702
			LVEDD acc. to HENRY'							-0.528	0.184	
			LVEDD acc. to HENRY''							1.588	0.561	
CD40_L	CD40LG	CD40 ligand	LVEDD acc. to HENRY	-0.116	0.050	0.288	-0.112	0.061	0.483	-0.185	0.123	0.810
CD4	CD4	T-cell surface glycoprotein CD4	LVEDD acc. to HENRY	0.021	0.053	0.880	0.016	0.062	0.902	0.057	0.125	0.954
CD84	CD84	SLAM family member 5	LVEDD acc. to HENRY	-0.057	0.056	0.695	-0.071	0.062	0.661	0.008	0.125	0.991
CEACAM8	CEACAM8	Carcinoembryonic antigen-related cell adhesion molecule 8	LVEDD acc. to HENRY	-0.096	0.059	0.461	-0.068	0.061	0.678	-0.202	0.122	0.786
CTRC	CTRC	Chymotrypsin-C	LVEDD acc. to HENRY	-0.003	0.060	0.980	0.006	0.061	0.966	-0.051	0.125	0.959
CTSL1	CTSL	Cathepsin L1	LVEDD acc. to HENRY	0.055	0.060	0.701	0.086	0.061	0.521	-0.115	0.124	0.938
CXCL1	CXCL1	Growth-regulated alpha protein	LVEDD acc. to HENRY	-0.027	0.046	0.819	0.039	0.281	0.404	-0.189	0.123	0.810
			LVEDD acc. to HENRY'				0.074	0.092				
			LVEDD acc. to HENRY''				-0.340	0.275				
DCN	DCN	Decorin	LVEDD acc. to HENRY	-0.005	0.054	0.974	0.016	0.062	0.902	-0.099	0.122	0.944
DECR1	DECR1	2,4-dienoyl-CoA reductase, mitochondrial	LVEDD acc. to HENRY	-0.021	0.045	0.853	-0.051	0.062	0.770	0.088	0.123	0.944
Dkk_1	DKK1	Dickkopf-related protein 1	LVEDD acc. to HENRY	-0.070	0.056	0.614	-0.057	0.062	0.715	-0.119	0.124	0.938
FABP2	FABP2	Fatty acid-binding protein, intestinal	LVEDD acc. to HENRY	0.755	0.272	0.385	0.736	0.284	0.496	0.091	0.124	0.944
			LVEDD acc. to HENRY'	-0.235	0.091		-0.237	0.093				
			LVEDD acc. to HENRY''	0.626	0.255		0.680	0.278				
FGF_21	FGF21	Fibroblast growth factor 21	LVEDD acc. to HENRY	0.069	0.056	0.621	0.074	0.061	0.626	0.043	0.125	0.959
FGF_23	FGF23	Fibroblast growth factor 23	LVEDD acc. to HENRY	0.119	0.067	0.420	0.116	0.061	0.453	0.006	0.125	0.992
FS	FST	Follistatin	LVEDD acc. to HENRY	0.057	0.052	0.672	0.091	0.061	0.521	-0.048	0.124	0.959
GDF_2	GDF2	Growth/differentiation factor 2	LVEDD acc. to HENRY	-0.035	0.053	0.799	-0.032	0.062	0.849	-0.056	0.120	0.954
GH	GH1	Somatotropin	LVEDD acc. to HENRY	0.018	0.047	0.883	0.035	0.062	0.833	-1.408	0.529	0.702
			LVEDD acc. to HENRY'							0.551	0.177	
			LVEDD acc. to HENRY''							-1.731	0.540	
GIF	CBLIF	Gastric intrinsic factor	LVEDD acc. to HENRY	-0.007	0.061	0.967	-0.016	0.062	0.902	0.036	0.123	0.959
GLO1	GLO1	Lactoylglutathione lyase	LVEDD acc. to HENRY	-0.007	0.053	0.959	-0.011	0.062	0.926	0.007	0.125	0.992
GT	FABP6	Gastrotropin	LVEDD acc. to HENRY	0.058	0.055	0.692	0.100	0.061	0.521	-0.083	0.124	0.944
Gal_9	LGALS9	Galectin-9	LVEDD acc. to HENRY	-0.001	0.053	0.985	0.011	0.061	0.931	-0.042	0.125	0.959
HAOX1	HAO1	Hydroxyacid oxidase 1	LVEDD acc. to HENRY	-0.057	0.057	0.695	-0.057	0.062	0.715	-0.043	0.124	0.959
HB_EGF	HBEGF	Proheparin-binding EGF-like growth factor [Cleaved into: Heparin-binding EGF-like growth factor	LVEDD acc. to HENRY	-0.065	0.055	0.633	-0.046	0.062	0.822	-0.153	0.123	0.869
HO_1	HMOX1	Heme oxygenase 1	LVEDD acc. to HENRY	0.007	0.057	0.963	-0.006	0.062	0.966	0.067	0.125	0.949
HSP_27	HSPB1	Heat shock protein beta-1	LVEDD acc. to HENRY	-0.002	0.031	0.974	-0.012	0.062	0.926	0.036	0.124	0.959
IDUA	IDUA	Alpha-L-iduronidase	LVEDD acc. to HENRY	-0.030	0.060	0.830	0.019	0.061	0.886	-0.226	0.122	0.702
IL16	IL16	Pro-interleukin-16 [Cleaved into: Interleukin-16	LVEDD acc. to HENRY	-0.071	0.061	0.646	-0.030	0.062	0.849	-0.204	0.122	0.786
IL18	IL18	Interleukin-18	LVEDD acc. to HENRY	-0.067	0.055	0.623	-0.063	0.061	0.711	1.352	0.559	0.702
			LVEDD acc. to HENRY'							-0.460	0.187	
			LVEDD acc. to HENRY''							1.298	0.570	
IL1RL2	IL1RL2	Interleukin-1 receptor-like 2	LVEDD acc. to HENRY	-0.087	0.056	0.487	-0.065	0.062	0.699	-0.155	0.122	0.869
IL6	IL6	Interleukin-6	LVEDD acc. to HENRY	-0.016	0.057	0.910	-0.007	0.062	0.966	1.413	0.553	0.702
			LVEDD acc. to HENRY'							-0.506	0.185	
			LVEDD acc. to HENRY''							1.482	0.564	
IL_17D	IL17D	Interleukin-17D	LVEDD acc. to HENRY	-0.016	0.056	0.910	-0.021	0.062	0.875	0.008	0.122	0.991
IL_1ra	IL1RN	Interleukin-1 receptor antagonist protein	LVEDD acc. to HENRY	-0.085	0.059	0.538	-0.059	0.062	0.711	-0.166	0.123	0.857
IL_27	IL27	Interleukin-27	LVEDD acc. to HENRY	0.042	0.055	0.766	0.040	0.062	0.833	0.051	0.122	0.959
IL_4RA	IL4R	Interleukin-4 receptor subunit alpha	LVEDD acc. to HENRY	-0.056	0.056	0.695	-0.071	0.061	0.647	0.048	0.125	0.959
ITGB1BP2	ITGB1BP2	Integrin beta-1-binding protein 2	LVEDD acc. to HENRY	-0.044	0.045	0.695	-0.061	0.062	0.711	-0.026	0.125	0.970
IgG_Fc_receptor_II	FCGR2B	Low affinity immunoglobulin gamma Fc region receptor II-b	LVEDD acc. to HENRY	0.009	0.057	0.946	0.005	0.062	0.966	0.033	0.124	0.961
KIM1	HAVCR1	Hepatitis A virus cellular receptor 1	LVEDD acc. to HENRY	-0.004	0.061	0.979	-0.028	0.062	0.849	1.357	0.558	0.702
			LVEDD acc. to HENRY'							-0.475	0.187	
			LVEDD acc. to HENRY''							1.465	0.570	
LEP	LEP	Leptin	LVEDD acc. to HENRY	-0.171	0.051	0.068	-0.188	0.060	0.135	-0.141	0.123	0.872
LOX_1	OLR1	Oxidized low-density lipoprotein receptor 1	LVEDD acc. to HENRY	-0.114	0.063	0.420	-0.079	0.062	0.585	-0.184	0.123	0.810
LPL	LPL	Lipoprotein lipase	LVEDD acc. to HENRY	0.072	0.051	0.547	0.088	0.062	0.521	0.025	0.123	0.970
MARCO	MARCO	Macrophage receptor MARCO	LVEDD acc. to HENRY	-0.051	0.059	0.717	-0.017	0.062	0.898	-0.168	0.123	0.857
MERTK	MERTK	Tyrosine-protein kinase Mer	LVEDD acc. to HENRY	-0.012	0.062	0.938	-0.026	0.062	0.866	0.063	0.124	0.949
MMP12	MMP12	Macrophage metalloelastase	LVEDD acc. to HENRY	0.020	0.057	0.895	0.021	0.062	0.875	0.016	0.125	0.982
MMP7	MMP7	Matrilysin	LVEDD acc. to HENRY	0.015	0.041	0.889	-0.004	0.062	0.966	0.133	0.124	0.891
NEMO	IKBKG	NF-kappa-B essential modulator	LVEDD acc. to HENRY	-0.024	0.044	0.822	-0.048	0.062	0.804	0.043	0.125	0.959

PAPPA	PAPPA	Pappalysin-1	LVEDD acc. to HENRY	0.061	0.065	0.701	0.085	0.062	0.533	-0.079	0.119	0.944
PARP_1	PARP1	Poly [ADP-ribose] polymerase 1	LVEDD acc. to HENRY	-0.068	0.062	0.672	-0.037	0.062	0.833	-0.178	0.123	0.810
PAR_1	F2R	Proteinase-activated receptor 1	LVEDD acc. to HENRY	-0.046	0.053	0.717	-0.036	0.062	0.833	-0.096	0.124	0.944
PDGF_subunit_B	PDGFB	Platelet-derived growth factor subunit B	LVEDD acc. to HENRY	-0.041	0.050	0.732	-0.101	0.280	0.506	-0.162	0.123	0.857
			LVEDD acc. to HENRY'				0.101	0.092				
			LVEDD acc. to HENRY''				-0.399	0.274				
PD_L2	PDCD1LG2	Programmed cell death 1 ligand 2	LVEDD acc. to HENRY	-0.001	0.059	0.985	0.018	0.062	0.898	-0.095	0.124	0.944
PGF	PGF	Placenta growth factor	LVEDD acc. to HENRY	0.014	0.058	0.917	0.004	0.062	0.966	0.067	0.123	0.949
PIgR	PIGR	Polymeric immunoglobulin receptor	LVEDD acc. to HENRY	0.048	0.052	0.701	0.060	0.061	0.711	0.001	0.125	0.996
PRELP	PRELP	Prolargin	LVEDD acc. to HENRY	0.107	0.059	0.420	0.095	0.062	0.521	0.124	0.123	0.921
PRSS27	PRSS27	Serine protease 27	LVEDD acc. to HENRY	-0.048	0.056	0.717	-0.040	0.062	0.833	-0.078	0.124	0.949
PRSS8	PRSS8	Prostasin	LVEDD acc. to HENRY	-0.041	0.058	0.793	-0.025	0.062	0.866	-0.102	0.122	0.944
PSGL_1	SELPLG	P-selectin glycoprotein ligand 1	LVEDD acc. to HENRY	0.061	0.059	0.692	0.083	0.062	0.540	0.308	0.536	0.702
			LVEDD acc. to HENRY'							-0.338	0.179	
			LVEDD acc. to HENRY''							1.294	0.547	
PTX3	PTX3	Pentraxin-related protein PTX3	LVEDD acc. to HENRY	0.009	0.054	0.946	0.031	0.062	0.849	-0.092	0.124	0.944
RAGE	AGER	Advanced glycosylation end product-specific receptor	LVEDD acc. to HENRY	0.152	0.064	0.285	0.172	0.060	0.216	-0.085	0.123	0.944
REN	REN	Renin	LVEDD acc. to HENRY	0.142	0.061	0.285	0.145	0.061	0.365	0.064	0.124	0.949
SCF	KITLG	Kit ligand	LVEDD acc. to HENRY	-0.093	0.060	0.487	-0.109	0.062	0.496	0.029	0.124	0.970
SERPINA12	SERPINA12	Serpin A12	LVEDD acc. to HENRY	0.051	0.057	0.707	0.051	0.062	0.770	0.043	0.125	0.959
SLAMF7	SLAMF7	SLAM family member 7	LVEDD acc. to HENRY	0.013	0.056	0.917	0.053	0.062	0.767	-0.144	0.124	0.869
SOD2	SOD2	Superoxide dismutase [Mn], mitochondrial	LVEDD acc. to HENRY	0.070	0.064	0.672	0.110	0.062	0.496	-0.222	0.122	0.702
SORT1	SORT1	Sortilin	LVEDD acc. to HENRY	-0.011	0.059	0.938	0.013	0.062	0.920	-0.128	0.124	0.917
SPON2	SPON2	Spondin-2	LVEDD acc. to HENRY	0.020	0.059	0.902	0.038	0.062	0.833	-0.071	0.125	0.949
SRC	SRC	Proto-oncogene tyrosine-protein kinase Src	LVEDD acc. to HENRY	-0.021	0.041	0.829	-0.036	0.062	0.833	0.002	0.124	0.996
STK4	STK4	Serine/threonine-protein kinase 4	LVEDD acc. to HENRY	-0.016	0.040	0.880	-0.034	0.062	0.833	0.026	0.125	0.970
TF	F3	Tissue factor	LVEDD acc. to HENRY	-0.028	0.060	0.853	-0.045	0.061	0.822	0.074	0.124	0.949
TGM2	TGM2	Protein-glutamine gamma-glutamyltransferase 2	LVEDD acc. to HENRY	0.014	0.053	0.917	0.038	0.062	0.833	-0.092	0.124	0.944
THBS2	THBS2	Thrombospondin-2	LVEDD acc. to HENRY	0.868	0.270	0.285	0.797	0.280	0.365	-0.064	0.125	0.949
			LVEDD acc. to HENRY'	-0.238	0.090		-0.189	0.092				
			LVEDD acc. to HENRY''	0.592	0.252		0.468	0.274				
THPO	THPO	Thrombopoietin	LVEDD acc. to HENRY	-0.058	0.059	0.695	-0.071	0.062	0.662	0.043	0.124	0.959
TIE2	TEK	Angiopoietin-1 receptor	LVEDD acc. to HENRY	0.011	0.062	0.938	0.029	0.062	0.849	-0.083	0.124	0.944
TM	THBD	Thrombomodulin	LVEDD acc. to HENRY	-0.046	0.062	0.780	-0.019	0.062	0.889	-0.143	0.124	0.869
TNFRSF10A	TNFRSF10A	Tumor necrosis factor receptor superfamily member 10A	LVEDD acc. to HENRY	-0.007	0.057	0.963	-0.004	0.062	0.966	-0.023	0.124	0.970
TNFRSF11A	TNFRSF11A	Tumor necrosis factor receptor superfamily member 11A	LVEDD acc. to HENRY	-0.104	0.063	0.455	-0.096	0.061	0.521	-0.066	0.124	0.949
TNFRSF13B	TNFRSF13B	Tumor necrosis factor receptor superfamily member 13B	LVEDD acc. to HENRY	0.056	0.056	0.695	0.062	0.062	0.711	0.026	0.125	0.970
TRAIL_R2	TNFRSF10B	Tumor necrosis factor receptor superfamily member 10B	LVEDD acc. to HENRY	0.034	0.061	0.819	0.040	0.061	0.833	-0.017	0.125	0.982
VEGFD	VEGFD	Vascular endothelial growth factor D	LVEDD acc. to HENRY	**0.215**	**0.050**	**0.002**	**0.260**	**0.059**	**0.004**	0.067	0.125	0.949
VSIG2	VSIG2	V-set and immunoglobulin domain-containing protein 2	LVEDD acc. to HENRY	0.034	0.062	0.819	0.029	0.061	0.849	0.034	0.125	0.961
XCL1	XCL1	Lymphotactin	LVEDD acc. to HENRY	0.046	0.050	0.701	0.061	0.062	0.711	1.736	0.525	0.557
			LVEDD acc. to HENRY'							-0.692	0.175	
			LVEDD acc. to HENRY''							2.170	0.536	
hOSCAR	OSCAR	Osteoclast-associated immunoglobulin-like receptor	LVEDD acc. to HENRY	0.003	0.061	0.985	0.004	0.061	0.966	-0.005	0.125	0.992
**CVD3 Panel**												
ALCAM	ALCAM	CD166 antigen	LVEDD acc. to HENRY	-0.060	0.062	0.695	-0.034	0.061	0.836	-0.133	0.120	0.872
AP_N	ANPEP	Aminopeptidase N	LVEDD acc. to HENRY	-0.055	0.061	0.706	-0.036	0.061	0.833	-0.116	0.122	0.938
AXL	AXL	Tyrosine-protein kinase receptor UFO	LVEDD acc. to HENRY	-0.041	0.060	0.793	-0.026	0.061	0.863	-0.078	0.121	0.945
AZU1	AZU1	Azurocidin	LVEDD acc. to HENRY	-0.083	0.051	0.455	-0.061	0.062	0.711	-0.210	0.121	0.753
BLM_hydrolase	BLMH	Bleomycin hydrolase	LVEDD acc. to HENRY	-0.092	0.060	0.491	-0.092	0.061	0.521	-0.035	0.121	0.959
CASP_3	CASP3	Caspase-3	LVEDD acc. to HENRY	-0.042	0.050	0.718	-0.059	0.062	0.711	0.008	0.125	0.991
CCL15	CCL15	C-C motif chemokine 15	LVEDD acc. to HENRY	0.004	0.055	0.974	0.013	0.061	0.920	-0.037	0.122	0.959
CCL16	CCL16	C-C motif chemokine 16	LVEDD acc. to HENRY	-0.020	0.053	0.883	-0.031	0.061	0.849	0.017	0.125	0.982
CCL24	CCL24	C-C motif chemokine 24	LVEDD acc. to HENRY	0.051	0.058	0.710	0.045	0.062	0.822	0.063	0.124	0.949
CD163	CD163	Scavenger receptor cysteine-rich type 1 protein M130	LVEDD acc. to HENRY	-0.027	0.057	0.853	-0.022	0.062	0.875	-0.041	0.123	0.959
CD93	CD93	Complement component C1q receptor	LVEDD acc. to HENRY	0.037	0.063	0.819	0.037	0.061	0.833	0.011	0.122	0.987
CDH5	CDH5	Cadherin-5	LVEDD acc. to HENRY	0.008	0.057	0.959	0.029	0.061	0.849	-0.072	0.123	0.949
CHI3L1	CHI3L1	Chitinase-3-like protein 1	LVEDD acc. to HENRY	-0.107	0.054	0.384	-0.091	0.061	0.521	0.879	0.543	0.702
			LVEDD acc. to HENRY'							-0.451	0.181	
			LVEDD acc. to HENRY''							1.446	0.554	
CHIT1	CHIT1	Chitotriosidase-1	LVEDD acc. to HENRY	-0.037	0.055	0.793	-0.006	0.062	0.966	-0.128	0.124	0.917
CNTN1	CNTN1	Contactin-1	LVEDD acc. to HENRY	-0.034	0.057	0.819	-0.020	0.061	0.875	-0.085	0.122	0.944
COL1A1	COL1A1	Collagen alpha-1	LVEDD acc. to HENRY	-0.020	0.059	0.902	0.011	0.062	0.926	-0.147	0.123	0.869
CPA1	CPA1	Carboxypeptidase A1	LVEDD acc. to HENRY	-0.080	0.056	0.539	-0.091	0.060	0.521	-0.016	0.125	0.982
CPB1	CPB1	Carboxypeptidase B	LVEDD acc. to HENRY	-0.056	0.057	0.695	-0.085	0.061	0.521	0.082	0.125	0.944
CSTB	CSTB	Cystatin-B	LVEDD acc. to HENRY	-0.079	0.063	0.614	-0.060	0.061	0.711	-0.106	0.124	0.944
CTSD	CTSD	Cathepsin D	LVEDD acc. to HENRY	-0.048	0.057	0.718	-0.004	0.062	0.966	-0.223	0.121	0.702
CTSZ	CTSZ	Cathepsin Z	LVEDD acc. to HENRY	-0.118	0.056	0.334	-0.103	0.060	0.506	-0.156	0.122	0.869
CXCL16	CXCL16	C-X-C motif chemokine 16	LVEDD acc. to HENRY	-0.060	0.061	0.695	-0.063	0.062	0.711	-0.024	0.123	0.970
DLK_1	DLK1	Protein delta homolog 1	LVEDD acc. to HENRY	-0.069	0.054	0.610	-0.084	0.060	0.526	-0.008	0.123	0.991
EGFR	EGFR	Epidermal growth factor receptor	LVEDD acc. to HENRY	-0.025	0.056	0.862	-0.016	0.062	0.902	-0.064	0.120	0.949
EPHB4	EPHB4	Ephrin type-B receptor 4	LVEDD acc. to HENRY	-0.072	0.061	0.646	-0.097	0.060	0.521	0.080	0.122	0.944
Ep_CAM	EPCAM	Epithelial cell adhesion molecule	LVEDD acc. to HENRY	0.010	0.053	0.938	0.014	0.061	0.911	-0.001	0.124	0.996
FABP4	FABP4	Fatty acid-binding protein, adipocyte	LVEDD acc. to HENRY	-0.038	0.059	0.804	-0.057	0.061	0.715	0.048	0.125	0.959
FAS	FAS	Tumor necrosis factor receptor superfamily member 6	LVEDD acc. to HENRY	-0.095	0.056	0.455	-0.090	0.061	0.521	-0.101	0.122	0.944
GDF_15	GDF15	Growth/differentiation factor 15	LVEDD acc. to HENRY	-0.015	0.057	0.917	-0.028	0.061	0.849	0.059	0.125	0.954
GP6	GP6	Platelet glycoprotein VI	LVEDD acc. to HENRY	-0.101	0.054	0.400	-0.106	0.062	0.506	-0.092	0.124	0.944

GRN	GRN	Granulins	LVEDD acc. to HENRY	-0.023	0.057	0.880	-0.001	0.061	0.994	-0.113	0.122	0.938
Gal_3	LGALS3	Galectin-3	LVEDD acc. to HENRY	-0.024	0.060	0.880	-0.018	0.062	0.898	-0.045	0.123	0.959
Gal_4	LGALS4	Galectin-4	LVEDD acc. to HENRY	-0.047	0.056	0.717	-0.059	0.061	0.711	0.929	0.554	0.702
			LVEDD acc. to HENRY'							-0.445	0.185	
			LVEDD acc. to HENRY''							1.493	0.566	
ICAM_2	ICAM2	Intercellular adhesion molecule 2	LVEDD acc. to HENRY	-0.063	0.055	0.664	-0.082	0.061	0.539	0.026	0.121	0.970
IGFBP_1	IGFBP1	Insulin-like growth factor-binding protein 1	LVEDD acc. to HENRY	0.086	0.052	0.455	0.104	0.060	0.506	0.043	0.556	0.810
			LVEDD acc. to HENRY'							0.150	0.186	
			LVEDD acc. to HENRY''							-0.669	0.568	
IGFBP_2	IGFBP2	Insulin-like growth factor-binding protein 2	LVEDD acc. to HENRY	-0.001	0.056	0.985	-0.009	0.061	0.945	0.036	0.124	0.959
IGFBP_7	IGFBP7	Insulin-like growth factor-binding protein 7	LVEDD acc. to HENRY	-0.002	0.062	0.985	-0.004	0.061	0.966	0.012	0.124	0.987
IL2_RA	IL2RA	Interleukin-2 receptor subunit alpha	LVEDD acc. to HENRY	0.014	0.057	0.917	0.030	0.061	0.849	-0.063	0.122	0.949
IL_17RA	IL17RA	Interleukin-17 receptor A	LVEDD acc. to HENRY	-0.071	0.056	0.610	-0.090	0.061	0.521	0.021	0.124	0.970
IL_18BP	IL18BP	Interleukin-18-binding protein	LVEDD acc. to HENRY	-0.112	0.060	0.400	-0.102	0.060	0.506	-0.097	0.122	0.944
IL_1RT1	IL1R1	Interleukin-1 receptor type 1	LVEDD acc. to HENRY	-0.042	0.062	0.793	-0.036	0.061	0.833	-0.040	0.117	0.959
IL_1RT2	IL1R2	Interleukin-1 receptor type 2	LVEDD acc. to HENRY	-0.128	0.058	0.322	-0.118	0.061	0.449	-0.136	0.122	0.872
IL_6RA	IL6R	Interleukin-6 receptor subunit alpha	LVEDD acc. to HENRY	-0.058	0.057	0.695	-0.067	0.061	0.678	-0.014	0.124	0.982
ITGB2	ITGB2	Integrin beta-2	LVEDD acc. to HENRY	-0.084	0.053	0.487	-0.064	0.061	0.700	-0.183	0.123	0.810
JAM_A	F11R	Junctional adhesion molecule A	LVEDD acc. to HENRY	-0.032	0.062	0.829	-0.043	0.061	0.823	0.042	0.123	0.959
KLK6	KLK6	Kallikrein-6	LVEDD acc. to HENRY	0.001	0.059	0.992	-0.043	0.062	0.823	0.175	0.119	0.810
LDL_receptor	LDLR	Low-density lipoprotein receptor	LVEDD acc. to HENRY	-0.042	0.058	0.791	-0.017	0.061	0.898	-0.128	0.124	0.917
LTBR	LTBR	Tumor necrosis factor receptor superfamily member 3	LVEDD acc. to HENRY	-0.146	0.061	0.285	-0.142	0.060	0.365	-0.058	0.123	0.954
MB	MB	Myoglobin	LVEDD acc. to HENRY	-0.085	0.055	0.487	-0.125	0.061	0.404	0.086	0.124	0.944
MCP_1	CCL2	C-C motif chemokine 2	LVEDD acc. to HENRY	-0.008	0.066	0.963	0.000	0.062	0.994	-0.032	0.124	0.961
MEPE	MEPE	Matrix extracellular phosphoglycoprotein	LVEDD acc. to HENRY	-0.114	0.053	0.334	-0.133	0.061	0.404	-0.048	0.125	0.959
MMP_2	MMP2	72 kDa type IV collagenase	LVEDD acc. to HENRY	0.129	0.062	0.334	0.146	0.060	0.365	-0.039	0.123	0.959
MMP_3	MMP3	Stromelysin-1	LVEDD acc. to HENRY	0.451	0.256	0.385	0.022	0.061	0.875	0.123	0.124	0.938
			LVEDD acc. to HENRY'	-0.191	0.085							
			LVEDD acc. to HENRY''	0.586	0.240							
MMP_9	MMP9	Matrix metalloproteinase-9	LVEDD acc. to HENRY	-0.126	0.060	0.334	-0.095	0.061	0.521	-0.198	0.121	0.786
MPO	MPO	Myeloperoxidase	LVEDD acc. to HENRY	-0.076	0.056	0.582	-0.053	0.062	0.763	-0.150	0.122	0.869
NT_proBNP	NPPB	Natriuretic peptides B	LVEDD acc. to HENRY	**0.182**	**0.041**	**0.002**	**0.219**	**0.060**	**0.027**	0.317	0.118	0.702
Notch_3	NOTCH3	Neurogenic locus notch homolog protein 3	LVEDD acc. to HENRY	-0.045	0.063	0.791	-0.039	0.061	0.833	-0.037	0.124	0.959
OPG	TNFRSF11B	Tumor necrosis factor receptor superfamily member 11B	LVEDD acc. to HENRY	-0.021	0.065	0.902	-0.021	0.061	0.875	0.002	0.125	0.996
OPN	SPP1	Osteopontin	LVEDD acc. to HENRY	-0.082	0.055	0.518	-0.089	0.060	0.521	-0.044	0.125	0.959
PAI	SERPINE1	Plasminogen activator inhibitor 1	LVEDD acc. to HENRY	-0.033	0.054	0.816	0.003	0.061	0.966	-0.186	0.122	0.810
PCSK9	PCSK9	Proprotein convertase subtilisin/kexin type 9	LVEDD acc. to HENRY	-0.087	0.053	0.455	-0.081	0.060	0.539	0.633	0.545	0.702
			LVEDD acc. to HENRY'							-0.380	0.182	
			LVEDD acc. to HENRY''							1.288	0.557	
PDGF_subunit_A	PDGFA	Platelet-derived growth factor subunit A	LVEDD acc. to HENRY	-0.086	0.055	0.487	-0.062	0.061	0.711	-0.178	0.123	0.810
PECAM_1	PECAM1	Platelet endothelial cell adhesion molecule	LVEDD acc. to HENRY	-0.030	0.066	0.862	-0.021	0.062	0.875	-0.041	0.123	0.959
PGLYRP1	PGLYRP1	Peptidoglycan recognition protein 1	LVEDD acc. to HENRY	-0.107	0.056	0.385	-0.094	0.060	0.521	-0.148	0.122	0.869
PI3	PI3	Elafin	LVEDD acc. to HENRY	-0.031	0.054	0.819	-0.017	0.062	0.902	-0.111	0.124	0.943
PLC	HSPG2	Basement membrane-specific heparan sulfate proteoglycan core protein	LVEDD acc. to HENRY	-0.049	0.061	0.740	-0.042	0.061	0.823	-0.050	0.124	0.959
PON3	PON3	Serum paraoxonase/lactonase 3	LVEDD acc. to HENRY	-0.123	0.051	0.285	-0.134	0.061	0.404	-1.719	0.539	0.702
			LVEDD acc. to HENRY'							0.566	0.180	
			LVEDD acc. to HENRY''							-1.687	0.550	
PRTN3	PRTN3	Myeloblastin	LVEDD acc. to HENRY	-0.143	0.060	0.285	-0.157	0.061	0.310	-0.022	0.123	0.970
PSP_D	SFTPD	Pulmonary surfactant-associated protein D	LVEDD acc. to HENRY	0.020	0.056	0.895	0.060	0.061	0.711	-0.143	0.124	0.869
RARRES2	RARRES2	Retinoic acid receptor responder protein 2	LVEDD acc. to HENRY	-0.099	0.052	0.385	-0.115	0.059	0.449	-0.053	0.124	0.959
RETN	RETN	Resistin	LVEDD acc. to HENRY	-0.075	0.057	0.606	-0.080	0.060	0.540	-0.038	0.125	0.959
SCGB3A2	SCGB3A2	Secretoglobin family 3A member 2	LVEDD acc. to HENRY	0.124	0.055	0.319	0.143	0.061	0.365	0.030	0.124	0.970
SELE	SELE	E-selectin	LVEDD acc. to HENRY	-0.073	0.056	0.610	-0.063	0.061	0.711	-0.112	0.123	0.943
SELP	SELP	P-selectin	LVEDD acc. to HENRY	-0.045	0.062	0.789	-0.029	0.062	0.849	-0.090	0.122	0.944
SHPS_1	SIRPA	Tyrosine-protein phosphatase non-receptor type substrate 1	LVEDD acc. to HENRY	-0.022	0.057	0.881	-0.035	0.061	0.833	0.039	0.121	0.959
SPON1	SPON1	Spondin-1	LVEDD acc. to HENRY	0.043	0.065	0.799	0.076	0.062	0.626	-0.110	0.122	0.943
ST2	IL1RL1	Interleukin-1 receptor-like 1	LVEDD acc. to HENRY	0.021	0.060	0.896	0.038	0.061	0.833	-0.062	0.125	0.949
TFF3	TFF3	Trefoil factor 3	LVEDD acc. to HENRY	0.032	0.057	0.819	0.047	0.060	0.804	0.012	0.124	0.987
TFPI	TFPI	Tissue factor pathway inhibitor	LVEDD acc. to HENRY	0.019	0.061	0.905	0.056	0.062	0.729	-0.116	0.124	0.938
TIMP4	TIMP4	Metalloproteinase inhibitor 4	LVEDD acc. to HENRY	-0.070	0.056	0.614	-0.066	0.061	0.680	-0.077	0.125	0.949
TLT_2	TREML2	Trem-like transcript 2 protein	LVEDD acc. to HENRY	-0.163	0.058	0.265	-0.164	0.060	0.229	-0.110	0.123	0.943
TNFRSF10C	TNFRSF10C	Tumor necrosis factor receptor superfamily member 10C	LVEDD acc. to HENRY	-0.136	0.056	0.285	-0.123	0.060	0.404	-0.161	0.119	0.857
TNFRSF14	TNFRSF14	Tumor necrosis factor receptor superfamily member 14	LVEDD acc. to HENRY	-0.091	0.062	0.521	-0.100	0.060	0.521	0.027	0.124	0.970
TNFSF13B	TNFSF13B	Tumor necrosis factor ligand superfamily member 13B	LVEDD acc. to HENRY	0.126	0.061	0.334	0.125	0.061	0.404	0.064	0.120	0.949
TNF_R1	TNFRSF1A	Tumor necrosis factor receptor superfamily member 1A	LVEDD acc. to HENRY	-0.106	0.060	0.420	-0.110	0.060	0.473	-0.023	0.124	0.970
TNF_R2	TNFRSF1B	Tumor necrosis factor receptor superfamily member 1B	LVEDD acc. to HENRY	-0.109	0.061	0.420	-0.106	0.060	0.496	-0.047	0.123	0.959
TR_AP	ACP5	Tartrate-resistant acid phosphatase type 5	LVEDD acc. to HENRY	-0.162	0.063	0.285	-0.143	0.061	0.365	-0.136	0.122	0.872
TR	TFRC	Transferrin receptor protein 1	LVEDD acc. to HENRY	0.024	0.061	0.881	0.039	0.062	0.833	-0.061	0.123	0.949
U_PAR	PLAUR	Urokinase plasminogen activator surface receptor	LVEDD acc. to HENRY	-0.040	0.056	0.791	-0.030	0.060	0.849	-0.074	0.123	0.949
t_PA	PLAT	Tissue-type plasminogen activator	LVEDD acc. to HENRY	0.048	0.058	0.721	0.074	0.061	0.626	-0.070	0.125	0.949
uPA	PLAU	Urokinase-type plasminogen activator	LVEDD acc. to HENRY	-0.055	0.060	0.701	-0.033	0.061	0.842	-0.112	0.122	0.940
vWF	VWF	von Willebrand factor	LVEDD acc. to HENRY	-0.032	0.056	0.819	-0.042	0.062	0.833	0.014	0.124	0.982
**Inflammation Panel**												
ADA	ADA	Adenosine deaminase	LVEDD acc. to HENRY	-0.083	0.064	0.610	-0.045	0.062	0.822	-0.232	0.120	0.702
ARTN	ARTN	Artemin	LVEDD acc. to HENRY	0.048	0.061	0.754	0.097	0.061	0.521	-0.166	0.113	0.810
AXIN1	AXIN1	Axin-1	LVEDD acc. to HENRY	-0.062	0.048	0.610	-0.087	0.062	0.525	-0.005	0.125	0.992
Beta_NGF	NGF	Beta-nerve growth factor	LVEDD acc. to HENRY	-0.048	0.045	0.692	-0.041	0.062	0.833	-0.137	0.124	0.872

CASP_8	CASP8	Caspase-8	LVEDD acc. to HENRY	-0.099	0.059	0.455	-0.093	0.062	0.521	-0.096	0.124	0.944
CCL3	CCL3	C-C motif chemokine 3	LVEDD acc. to HENRY	0.007	0.055	0.959	0.028	0.062	0.849	1.275	0.559	0.702
										-0.487	0.187	
										1.458	0.571	
CCL11	CCL11	Eotaxin	LVEDD acc. to HENRY	0.038	0.056	0.793	0.067	0.062	0.680	-0.080	0.124	0.945
CCL19	CCL19	C-C motif chemokine 19	LVEDD acc. to HENRY	-0.023	0.054	0.876	-0.068	0.062	0.678	0.174	0.122	0.819
CCL20	CCL20	C-C motif chemokine 20	LVEDD acc. to HENRY	-0.039	0.057	0.793	-0.025	0.062	0.866	1.107	0.560	0.702
			LVEDD acc. to HENRY'							-0.470	0.187	
			LVEDD acc. to HENRY''							1.463	0.571	
CCL23	CCL23	C-C motif chemokine 23	LVEDD acc. to HENRY	-0.014	0.051	0.917	-0.059	0.062	0.711	0.226	0.121	0.702
CCL25	CCL25	C-C motif chemokine 25	LVEDD acc. to HENRY	0.044	0.059	0.780	0.024	0.062	0.866	0.104	0.122	0.944
CCL28	CCL28	C-C motif chemokine 28	LVEDD acc. to HENRY	0.041	0.066	0.816	0.090	0.062	0.521	-0.156	0.119	0.862
CCL4	CCL4	C-C motif chemokine 4	LVEDD acc. to HENRY	-0.038	0.057	0.793	0.005	0.062	0.966	-0.247	0.120	0.702
CD244	CD244	Natural killer cell receptor 2B4	LVEDD acc. to HENRY	-0.130	0.060	0.330	-0.098	0.062	0.521	-0.227	0.122	0.702
CD40	CD40	Tumor necrosis factor receptor superfamily member 5	LVEDD acc. to HENRY	-0.060	0.060	0.695	-0.047	0.061	0.805	-0.093	0.124	0.944
CD5	CD5	T-cell surface glycoprotein CD5	LVEDD acc. to HENRY	-0.053	0.059	0.706	-0.038	0.061	0.833	-0.095	0.124	0.944
CD6	CD6	T-cell differentiation antigen CD6	LVEDD acc. to HENRY	-0.004	0.060	0.974	0.026	0.062	0.866	-0.138	0.124	0.872
CD8A	CD8A	T-cell surface glycoprotein CD8 alpha chain	LVEDD acc. to HENRY	0.012	0.054	0.923	0.032	0.061	0.849	-0.079	0.123	0.945
CDCP1	CDCP1	CUB domain-containing protein 1	LVEDD acc. to HENRY	-0.068	0.055	0.621	-0.036	0.062	0.833	1.171	0.549	0.702
			LVEDD acc. to HENRY'							-0.478	0.184	
			LVEDD acc. to HENRY''							1.409	0.561	
CSF_1	CSF1	Macrophage colony-stimulating factor 1	LVEDD acc. to HENRY	-0.115	0.059	0.385	-0.097	0.061	0.521	-0.142	0.122	0.869
CST5	CST5	Cystatin-D	LVEDD acc. to HENRY	-0.112	0.056	0.384	-0.119	0.062	0.449	-0.066	0.124	0.949
CX3CL1	CX3CL1	Fractalkine	LVEDD acc. to HENRY	-0.096	0.058	0.455	-0.095	0.061	0.521	-0.074	0.124	0.949
CXCL10	CXCL10	C-X-C motif chemokine 10	LVEDD acc. to HENRY	-0.034	0.059	0.819	0.014	0.062	0.909	-0.217	0.121	0.702
CXCL11	CXCL11	C-X-C motif chemokine 11	LVEDD acc. to HENRY	-0.060	0.053	0.668	-0.021	0.061	0.875	-0.230	0.122	0.702
CXCL1	CXCL1	Growth-regulated alpha protein	LVEDD acc. to HENRY	-0.043	0.046	0.701	-0.190	0.284	0.404	-0.182	0.123	0.810
			LVEDD acc. to HENRY'				0.136	0.092				
			LVEDD acc. to HENRY''				-0.508	0.275				
CXCL5	CXCL5	C-X-C motif chemokine 5	LVEDD acc. to HENRY	-0.074	0.052	0.539	-0.270	0.280	0.365	-0.164	0.123	0.857
			LVEDD acc. to HENRY'				0.149	0.091				
			LVEDD acc. to HENRY''				-0.547	0.272				
CXCL6	CXCL6	C-X-C motif chemokine 6	LVEDD acc. to HENRY	-0.073	0.053	0.556	-0.059	0.062	0.711	-0.149	0.123	0.869
CXCL9	CXCL9	C-X-C motif chemokine 9	LVEDD acc. to HENRY	0.016	0.055	0.910	0.057	0.062	0.715	-0.133	0.121	0.872
DNER	DNER	Delta and Notch-like epidermal growth factor-related receptor	LVEDD acc. to HENRY	-0.038	0.059	0.800	-0.028	0.061	0.849	-0.072	0.123	0.949
EN_RAGE	S100A12	Protein S100-A12	LVEDD acc. to HENRY	0.781	0.316	0.455	0.035	0.062	0.833	-0.061	0.124	0.949
			LVEDD acc. to HENRY'	-0.225	0.105							
			LVEDD acc. to HENRY''	0.563	0.294							
FGF_19	FGF19	Fibroblast growth factor 19	LVEDD acc. to HENRY	-0.038	0.053	0.791	-0.052	0.062	0.770	0.011	0.123	0.987
FGF_21	FGF21	Fibroblast growth factor 21	LVEDD acc. to HENRY	0.051	0.056	0.706	0.052	0.061	0.769	0.043	0.125	0.959
FGF_23	FGF23	Fibroblast growth factor 23	LVEDD acc. to HENRY	0.095	0.068	0.556	0.086	0.061	0.521	0.023	0.125	0.970
FGF_5	FGF5	Fibroblast growth factor 5	LVEDD acc. to HENRY	0.042	0.065	0.799	0.068	0.062	0.680	-0.082	0.114	0.944
Flt3L	FLT3LG	Fms-related tyrosine kinase 3 ligand	LVEDD acc. to HENRY	-0.071	0.055	0.610	-0.089	0.062	0.521	-0.005	0.124	0.992
GDNF	GDNF	Glial cell line-derived neurotrophic factor	LVEDD acc. to HENRY	-0.049	0.061	0.743	-0.025	0.062	0.866	-0.119	0.123	0.938
HGF	HGF	Hepatocyte growth factor	LVEDD acc. to HENRY	0.004	0.047	0.974	0.035	0.062	0.833	-0.106	0.122	0.944
IFN_gamma	IFNG	Interferon gamma	LVEDD acc. to HENRY	-0.041	0.059	0.793	-0.022	0.061	0.875	1.195	0.557	0.702
			LVEDD acc. to HENRY'							-0.494	0.186	
			LVEDD acc. to HENRY''							1.515	0.568	
IL10	IL10	Interleukin-10	LVEDD acc. to HENRY	0.011	0.048	0.917	0.072	0.061	0.647	-0.145	0.122	0.869
IL13	IL13	Interleukin-13	LVEDD acc. to HENRY	0.038	0.066	0.819	0.038	0.062	0.833	0.019	0.125	0.981
IL18	IL18	Interleukin-18	LVEDD acc. to HENRY	-0.123	0.054	0.319	-0.131	0.061	0.404	-0.090	0.124	0.944
IL2	IL2	Interleukin-2	LVEDD acc. to HENRY	-0.027	0.059	0.858	-0.024	0.062	0.866	-0.033	0.124	0.961
IL33	IL33	Interleukin-33	LVEDD acc. to HENRY	-0.042	0.048	0.717	-0.070	0.062	0.665	0.030	0.123	0.970
IL4	IL4	Interleukin-4	LVEDD acc. to HENRY	-0.015	0.059	0.917	-0.015	0.062	0.909	-0.014	0.120	0.982
IL5	IL5	Interleukin-5	LVEDD acc. to HENRY	-0.081	0.047	0.450	-0.109	0.062	0.496	-0.042	0.124	0.959
IL6	IL6	Interleukin-6	LVEDD acc. to HENRY	-0.029	0.058	0.830	-0.024	0.062	0.866	1.434	0.555	0.702
			LVEDD acc. to HENRY'							-0.502	0.186	
			LVEDD acc. to HENRY''							1.458	0.566	
IL7	IL7	Interleukin-7	LVEDD acc. to HENRY	-0.097	0.054	0.420	-0.075	0.062	0.626	-0.218	0.122	0.702
IL8	CXCL8	Interleukin-8	LVEDD acc. to HENRY	-0.066	0.054	0.623	-0.042	0.062	0.830	1.246	0.552	0.702
			LVEDD acc. to HENRY'							-0.495	0.184	
			LVEDD acc. to HENRY''							1.465	0.563	
IL_10RA	IL10RA	Interleukin-10 receptor subunit alpha	LVEDD acc. to HENRY	-0.053	0.053	0.695	-0.094	0.062	0.521	0.093	0.124	0.944
IL_10RB	IL10RB	Interleukin-10 receptor subunit beta	LVEDD acc. to HENRY	-0.158	0.057	0.265	-0.166	0.060	0.229	-0.087	0.124	0.944
IL_12B	IL12B	Interleukin-12 subunit beta	LVEDD acc. to HENRY	-0.018	0.058	0.902	-0.039	0.062	0.833	0.076	0.124	0.949
IL_15RA	IL15RA	Interleukin-15 receptor subunit alpha	LVEDD acc. to HENRY	-0.012	0.058	0.933	-0.012	0.061	0.926	-0.011	0.124	0.987
IL_17A	IL17A	Interleukin-17A	LVEDD acc. to HENRY	0.014	0.060	0.923	0.057	0.062	0.722	-0.154	0.122	0.869
IL_17C	IL17C	Interleukin-17C	LVEDD acc. to HENRY	0.054	0.056	0.699	0.052	0.062	0.769	0.059	0.124	0.954
IL_18R1	IL18R1	Interleukin-18 receptor 1	LVEDD acc. to HENRY	-0.132	0.056	0.285	-0.115	0.062	0.467	-0.191	0.121	0.810
IL_1_alpha	IL1A	Interleukin-1 alpha	LVEDD acc. to HENRY	0.027	0.052	0.829	0.031	0.062	0.849	0.023	0.125	0.970
IL_20RA	IL20RA	Interleukin-20 receptor subunit alpha	LVEDD acc. to HENRY	0.012	0.060	0.935	0.045	0.062	0.822	-0.165	0.123	0.857
IL_20	IL20	Interleukin-20	LVEDD acc. to HENRY	-0.139	0.056	0.285	-0.119	0.062	0.449	-0.258	0.120	0.702
IL_22_RA1	IL22RA1	Interleukin-22 receptor subunit alpha-1	LVEDD acc. to HENRY	-0.017	0.054	0.905	-0.024	0.062	0.866	0.003	0.125	0.996
IL_24	IL24	Interleukin-24	LVEDD acc. to HENRY	-0.061	0.057	0.677	-0.028	0.063	0.849	-0.167	0.126	0.857
IL_2RB	IL2RB	Interleukin-2 receptor subunit beta	LVEDD acc. to HENRY	-0.053	0.057	0.701	-0.016	0.061	0.902	-0.179	0.123	0.810
LAP_TGF_beta_1	TGFB1	Transforming growth factor beta-1 proprotein	LVEDD acc. to HENRY	-0.135	0.056	0.285	-0.130	0.062	0.404	-0.146	0.124	0.869
LIF	LIF	Leukemia inhibitory factor	LVEDD acc. to HENRY	0.060	0.065	0.701	0.044	0.062	0.823	0.119	0.124	0.938
LIF_R	LIFR	Leukemia inhibitory factor receptor	LVEDD acc. to HENRY	-0.093	0.060	0.487	-0.088	0.061	0.521	-0.069	0.125	0.949
MCP_1	CCL2	C-C motif chemokine 2	LVEDD acc. to HENRY	-0.053	0.059	0.706	-0.048	0.062	0.805	-0.058	0.125	0.954
MCP_2	CCL8	C-C motif chemokine 8	LVEDD acc. to HENRY	-0.024	0.057	0.876	-0.006	0.062	0.966	-0.096	0.124	0.944
MCP_3	CCL7	C-C motif chemokine 7	LVEDD acc. to HENRY	-0.091	0.054	0.455	-0.078	0.062	0.594	-0.158	0.123	0.869
MCP_4	CCL13	C-C motif chemokine 13	LVEDD acc. to HENRY	-0.072	0.057	0.614	-0.044	0.062	0.823	-0.174	0.123	0.829
MMP_10	MMP10	Stromelysin-2	LVEDD acc. to HENRY	0.032	0.057	0.819	0.060	0.061	0.711	-0.084	0.125	0.944
MMP_1	MMP1	Interstitial collagenase	LVEDD acc. to HENRY	-0.059	0.056	0.692	-0.275	0.283	0.404	-0.195	0.123	0.810
			LVEDD acc. to HENRY'				0.157	0.092				

			LVEDD acc. to HENRY''				-0.563	0.274				
NRTN	NRTN	Neurturin	LVEDD acc. to HENRY	-0.028	0.055	0.829	-0.024	0.062	0.866	-0.046	0.124	0.959
NT_3	NTF3	Neurotrophin-3	LVEDD acc. to HENRY	-0.042	0.290	0.420	0.092	0.062	0.521	-0.109	0.124	0.944
			LVEDD acc. to HENRY'	0.108	0.096							
			LVEDD acc. to HENRY''	-0.386	0.270							
OPG	TNFRSF11B	Tumor necrosis factor receptor superfamily member 11B	LVEDD acc. to HENRY	-0.038	0.066	0.819	-0.035	0.062	0.833	-0.021	0.124	0.970
OSM	OSM	Oncostatin-M	LVEDD acc. to HENRY	-0.144	0.059	0.285	-0.127	0.062	0.404	-0.162	0.122	0.857
PD_L1	CD274	Programmed cell death 1 ligand 1	LVEDD acc. to HENRY	-0.066	0.058	0.666	-0.085	0.062	0.533	0.037	0.124	0.959
SCF	KITLG	Kit ligand	LVEDD acc. to HENRY	-0.122	0.060	0.359	-0.141	0.062	0.365	0.018	0.125	0.982
SIRT2	SIRT2	NAD-dependent protein deacetylase sirtuin-2	LVEDD acc. to HENRY	-0.082	0.057	0.538	-0.088	0.062	0.521	-0.043	0.125	0.959
SLAMF1	SLAMF1	Signaling lymphocytic activation molecule	LVEDD acc. to HENRY	-0.106	0.058	0.420	-0.097	0.062	0.521	-0.117	0.124	0.938
ST1A1	SULT1A1	Sulfotransferase 1A1	LVEDD acc. to HENRY	-0.093	0.045	0.334	-0.120	0.062	0.449	-0.089	0.124	0.944
STAMBP	STAMBP	STAM-binding protein	LVEDD acc. to HENRY	-0.071	0.061	0.646	-0.075	0.062	0.626	-0.014	0.125	0.982
TGF_alpha	TGFA	Protransforming growth factor alpha [Cleaved into: Transforming growth factor alpha	LVEDD acc. to HENRY	-0.120	0.057	0.334	-0.118	0.061	0.449	-0.106	0.124	0.944
TNFB	LTA	Lymphotoxin-alpha	LVEDD acc. to HENRY	0.001	0.059	0.985	0.035	0.062	0.833	-0.145	0.120	0.869
TNFRSF9	TNFRSF9	Tumor necrosis factor receptor superfamily member 9	LVEDD acc. to HENRY	-0.054	0.055	0.695	-0.039	0.060	0.833	-0.101	0.124	0.944
TNFSF14	TNFSF14	Tumor necrosis factor ligand superfamily member 14	LVEDD acc. to HENRY	-0.125	0.057	0.330	-0.102	0.062	0.521	-0.206	0.122	0.786
TNF	TNF	Tumor necrosis factor	LVEDD acc. to HENRY	-0.053	0.054	0.695	-0.025	0.062	0.866	1.512	0.538	0.702
			LVEDD acc. to HENRY'							-0.541	0.180	
			LVEDD acc. to HENRY''							1.536	0.549	
TRAIL	TNFSF10	Tumor necrosis factor ligand superfamily member 10	LVEDD acc. to HENRY	-0.035	0.061	0.819	-0.008	0.062	0.956	-0.127	0.124	0.917
TRANCE	TNFSF11	Tumor necrosis factor ligand superfamily member 11	LVEDD acc. to HENRY	-0.019	0.060	0.902	-0.014	0.062	0.909	-0.033	0.124	0.961
TSLP	TSLP	Thymic stromal lymphopoietin	LVEDD acc. to HENRY	-0.107	0.057	0.400	-0.094	0.062	0.521	-0.153	0.123	0.869
TWEAK	TNFSF12	Tumor necrosis factor ligand superfamily member 12	LVEDD acc. to HENRY	0.021	0.064	0.902	0.050	0.062	0.781	-0.096	0.120	0.944
uPA	PLAU	Urokinase-type plasminogen activator	LVEDD acc. to HENRY	-0.065	0.060	0.672	-0.045	0.062	0.822	-0.120	0.124	0.938
VEGFA	VEGFA	Vascular endothelial growth factor A	LVEDD acc. to HENRY	-0.089	0.061	0.521	-0.083	0.061	0.539	-0.071	0.125	0.949
4E_BP1	EIF4EBP1	Eukaryotic translation initiation factor 4E-binding protein 1	LVEDD acc. to HENRY	-0.089	0.055	0.466	-0.092	0.062	0.521	-0.077	0.125	0.949

Supplemental Table S6. Associations of left ventricular ejection fraction (LVEF) and the metabolites.
Results from regression models adjusted for sex, age and BMI for the whole study population and from sex-specific models adjusted for age and BMI.
Metabolites were log2-transformed. Estimates are presented for z-transformed outcome and exposure variables.
* Biocrates updated the nomenclature for fatty acid and lipid classes in 2023 to the standard nomenclature used by LIPID MAPS
stderr, standard error; FDR, false discovery rate

				All subjects			Men			Women		
**Outcome**	**Biochemical**	**New nomenclatur***	**Exposure**	**beta**	**stderr**	**FDR**	**beta**	**stderr**	**FDR**	**beta**	**stderr**	**FDR**
**Acylcarnitines**												
C0	Carnitine	-	LVEF	-0.017	0.050	0.849	-0.082	0.058	0.344	0.203	0.108	0.938
C10	Decanoylcarnitine	-	LVEF	**-0.465**	**0.213**	**0.025**	**-0.155**	**0.058**	**0.039**	-0.020	0.120	0.990
			LVEF'	**0.044**	**0.057**							
			LVEF''	**-0.043**	**0.196**							
C10:1	Decenoylcarnitine	-	LVEF	**-0.488**	**0.212**	**0.032**	**-0.190**	**0.058**	**0.009**	0.136	0.119	0.946
			LVEF'	**0.055**	**0.057**							
			LVEF''	**-0.082**	**0.195**							
C10:2	Decadienylcarnitine	-	LVEF	-0.063	0.052	0.461	-0.107	0.058	0.184	0.122	0.122	0.946
C12	Dodecanoylcarnitine	-	LVEF	-0.428	0.216	0.126	-0.052	0.059	0.645	-0.125	0.122	0.946
			LVEF'	0.053	0.058							
			LVEF''	-0.074	0.199							
C12-DC	Dodecanedioylcarnitine	-	LVEF	0.071	0.052	0.394	0.057	0.058	0.622	0.136	0.121	0.946
C14	Tetradecanoylcarnitine	-	LVEF	-0.374	0.217	0.225	0.016	0.059	0.872	-0.050	0.123	0.961
			LVEF'	0.061	0.058							
			LVEF''	-0.105	0.200							
C14:2	Tetradecadienylcarnitine	-	LVEF	**-0.479**	**0.214**	**0.019**	**-0.446**	**0.230**	**0.023**	-0.118	0.122	0.946
			LVEF'	**0.046**	**0.058**		**0.030**	**0.065**				
			LVEF''	**-0.059**	**0.197**		**0.011**	**0.226**				
C16	Hexadecanoylcarnitine	-	LVEF	**-0.523**	**0.214**	**0.022**	**-0.534**	**0.230**	**0.025**	-0.115	0.123	0.946
			LVEF'	**0.070**	**0.057**		**0.079**	**0.065**				
			LVEF''	**-0.093**	**0.197**		**-0.104**	**0.227**				
C16:1	Hexadecenoylcarnitine	-	LVEF	-0.092	0.053	0.225	-0.104	0.058	0.197	-0.061	0.122	0.956
C16:2	Hexadecadienylcarnitine	-	LVEF	-0.053	0.053	0.560	-0.054	0.059	0.638	-0.083	0.123	0.956
C18	Octadecanoylcarnitine	-	LVEF	-0.326	0.216	0.197	-0.004	0.059	0.953	-0.056	0.123	0.961
			LVEF'	0.037	0.058							
			LVEF''	-0.015	0.199							
C18:1	Octadecenoylcarnitine	-	LVEF	**-0.587**	**0.208**	**0.003**	**-0.566**	**0.224**	**0.004**	-0.123	0.119	0.946
			LVEF'	**0.069**	**0.056**		**0.058**	**0.064**				
			LVEF''	**-0.137**	**0.192**		**-0.086**	**0.221**				
C2	Acetylcarnitine	-	LVEF	**-0.238**	**0.050**	**<0.001**	**-0.249**	**0.055**	**<0.001**	-0.250	0.107	0.810
C3	Propionylcarnitine	-	LVEF	0.009	0.049	0.897	-0.036	0.057	0.731	0.210	0.114	0.938
C4	Butyrylcarnitine	-	LVEF	-0.129	0.051	0.056	**-0.193**	**0.057**	**0.008**	0.128	0.116	0.946
C5	Valerylcarnitine	-	LVEF	-0.086	0.051	0.247	-0.127	0.058	0.110	0.081	0.115	0.956
C6 /C4:1-DC	Hexanoylcarnitine (Fumarylcarnitine)	-	LVEF	-0.062	0.053	0.470	-0.084	0.059	0.344	0.018	0.122	0.990
**Amino acids**												
Ala	Alanine	-	LVEF	-0.021	0.050	0.815	-0.053	0.058	0.638	0.137	0.120	0.946
Arg	Arginine	-	LVEF	**0.201**	**0.051**	**0.002**	**0.185**	**0.058**	**0.012**	0.269	0.115	0.810
Asn	Asparagine	-	LVEF	-0.011	0.052	0.886	-0.033	0.059	0.750	0.010	0.113	0.995
Asp	Aspartate	-	LVEF	0.033	0.053	0.744	-0.007	0.059	0.938	0.255	0.112	0.810
Cit	Citrulline	-	LVEF	0.010	0.050	0.886	-0.014	0.055	0.875	0.107	0.115	0.946
Gln	Glutamine	-	LVEF	0.062	0.052	0.461	-0.021	0.059	0.830	**0.433**	**0.106**	**0.019**
Glu	Glutamate	-	LVEF	-0.125	0.050	0.056	**-0.159**	**0.056**	**0.025**	0.011	0.116	0.995
Gly	Glycine	-	LVEF	-0.087	0.052	0.247	-0.087	0.058	0.305	-0.040	0.121	0.961
His	Histidine	-	LVEF	0.116	0.052	0.107	0.087	0.058	0.308	0.201	0.116	0.938
Ile	Isoleucine	-	LVEF	-0.035	0.049	0.716	-0.051	0.057	0.645	-1.018	0.464	0.946
			LVEF'							0.367	0.151	
			LVEF''							-1.016	0.430	
Leu	Leucine	-	LVEF	0.056	0.049	0.477	0.052	0.057	0.638	0.049	0.120	0.961
Lys	Lysine	-	LVEF	0.043	0.052	0.653	0.033	0.059	0.750	0.070	0.123	0.956
Met	Methionine	-	LVEF	-0.083	0.050	0.247	-0.143	0.058	0.065	0.167	0.121	0.946
Orn	Ornithine	-	LVEF	**-0.477**	**0.204**	**0.003**	**-0.263**	**0.056**	**<0.001**	0.109	0.116	0.946
			LVEF'	**0.034**	**0.055**							
			LVEF''	**-0.018**	**0.188**							
Phe	Phenylalanine	-	LVEF	-0.078	0.050	0.304	-0.139	0.058	0.069	0.190	0.118	0.946
Pro	Proline	-	LVEF	0.076	0.051	0.342	0.037	0.059	0.738	0.302	0.117	0.810
Ser	Serine	-	LVEF	**-0.157**	**0.053**	**0.022**	**-0.180**	**0.058**	**0.016**	-0.035	0.122	0.961
Thr	Threonine	-	LVEF	0.032	0.053	0.747	0.001	0.059	0.984	0.086	0.117	0.956
Trp	Tryptophan	-	LVEF	-0.011	0.050	0.886	-0.049	0.059	0.651	0.164	0.120	0.946
Tyr	Tyrosine	-	LVEF	**-0.172**	**0.049**	**0.005**	**-0.252**	**0.057**	**<0.001**	0.106	0.118	0.955
Val	Valine	-	LVEF	0.034	0.050	0.722	0.032	0.057	0.750	0.017	0.117	0.990
**Biogenic amines**												
ADMA	Asymmetric dimethylarginine	-	LVEF	-0.064	0.052	0.456	-0.114	0.058	0.150	0.165	0.118	0.946
Ac-Orn	Acetylornithine	-	LVEF	-0.112	0.053	0.126	-0.118	0.059	0.147	-0.078	0.124	0.956
Creatinine	Creatinine	-	LVEF	-0.103	0.048	0.119	-0.116	0.057	0.136	-0.088	0.116	0.956
DOPA	Dopamine	-	LVEF	-0.107	0.082	0.409	-0.079	0.094	0.651			
Histamine	Histamine	-	LVEF	0.050	0.055	0.610	0.048	0.061	0.669	0.080	0.127	0.956
Kynurenine	Kynurenine	-	LVEF	**-0.218**	**0.048**	**0.001**	**-0.301**	**0.055**	**<0.001**	0.151	0.104	0.946
Met-SO	Methionine sulfoxide	-	LVEF	**-0.153**	**0.052**	**0.022**	**-0.176**	**0.057**	**0.016**	-0.056	0.123	0.961
Putrescine	Putrescine	-	LVEF	-0.081	0.059	0.392	-0.087	0.065	0.391			
SDMA	Symmetric dimethylarginine	-	LVEF	**-0.409**	**0.209**	**0.012**	**-0.171**	**0.058**	**0.023**	-0.040	0.106	0.961
			LVEF'	**0.021**	**0.056**							
			LVEF''	**0.035**	**0.193**							
Serotonin	Serotonin	-	LVEF	-0.003	0.054	0.966	0.021	0.060	0.830	-0.115	0.125	0.955
Spermidine	Spermidine	-	LVEF	-0.092	0.052	0.225	-0.118	0.058	0.142	0.047	0.122	0.961

Spermine	Spermine	-	LVEF	0.023	0.053	0.815	0.006	0.059	0.938	0.120	0.122	0.946
Taurine	Taurine	-	LVEF	-0.116	0.052	0.107	-0.125	0.058	0.113	-0.063	0.115	0.956
alpha-AAA	Alpha-Aminoadipic acid	-	LVEF	-0.052	0.053	0.565	-0.040	0.060	0.722	-0.146	0.116	0.946
t4-OH-Pro	Trans-4-Hydroxyproline	-	LVEF	**-0.136**	**0.051**	**0.043**	**-0.166**	**0.057**	**0.023**	0.035	0.120	0.961
total DMA	Total Dimethylarginine	-	LVEF	**-0.183**	**0.048**	**0.003**	**-0.213**	**0.054**	**0.002**	-0.026	0.110	0.990
Arg/ADMA	Arginine/Asymmetric dimethylarginine	-	LVEF	**0.204**	**0.051**	**0.002**	**0.227**	**0.057**	**0.002**	0.060	0.117	0.956
Arg/SDMA	Arginine/Symmetric dimethylarginine	-	LVEF	**0.443**	**0.208**	**0.002**	**0.214**	**0.058**	**0.003**	0.159	0.107	0.946
			LVEF'	**-0.015**	**0.056**							
			LVEF''	**-0.054**	**0.193**							
Kynurenine/Trp	Kynurenine/Tryptophan	-	LVEF	**-0.219**	**0.049**	**0.001**	**-0.270**	**0.055**	**<0.001**	0.050	0.102	0.956
**Phosphatidylcholines**												
PC aa C24:0	-	PC 24:0	LVEF	0.023	0.053	0.815	0.031	0.058	0.772	-0.002	0.123	0.996
PC aa C26:0	-	PC 26:0	LVEF	0.006	0.053	0.955	0.030	0.059	0.780	-0.079	0.122	0.956
PC aa C28:1	-	PC 28:1	LVEF	0.045	0.050	0.612	0.047	0.056	0.651	0.070	0.116	0.956
PC aa C30:0	-	PC 30:0	LVEF	0.021	0.051	0.815	0.014	0.057	0.875	0.065	0.121	0.956
PC aa C30:2	-	PC 30:2	LVEF	-0.068	0.051	0.397	-0.097	0.057	0.230	0.083	0.116	0.956
PC aa C32:0	-	PC 32:0	LVEF	-0.105	0.051	0.130	-0.135	0.057	0.072	0.008	0.116	0.995
PC aa C32:1	-	PC 32:1	LVEF	0.034	0.052	0.730	0.034	0.057	0.750	0.005	0.122	0.995
PC aa C32:2	-	PC 32:2	LVEF	**0.148**	**0.050**	**0.022**	**0.158**	**0.056**	**0.025**	0.099	0.119	0.956
PC aa C32:3	-	PC 32:3	LVEF	0.105	0.048	0.115	0.113	0.057	0.147	0.122	0.116	0.946
PC aa C34:1	-	PC 34:1	LVEF	-0.022	0.052	0.815	-0.044	0.057	0.669	0.048	0.120	0.961
PC aa C34:2	-	PC 34:2	LVEF	0.042	0.052	0.656	0.045	0.057	0.669	-0.002	0.122	0.996
PC aa C34:3	-	PC 34:3	LVEF	0.091	0.050	0.213	0.096	0.056	0.229	0.046	0.119	0.961
PC aa C34:4	-	PC 34:4	LVEF	**0.175**	**0.051**	**0.007**	**0.194**	**0.057**	**0.007**	0.072	0.119	0.956
PC aa C36:0	-	PC 36:0	LVEF	-0.002	0.051	0.966	0.049	0.057	0.651	-0.219	0.109	0.938
PC aa C36:1	-	PC 36:1	LVEF	0.005	0.051	0.955	-0.021	0.057	0.830	0.156	0.116	0.946
PC aa C36:2	-	PC 36:2	LVEF	0.004	0.052	0.962	-0.006	0.057	0.938	0.068	0.122	0.956
PC aa C36:3	-	PC 36:3	LVEF	0.067	0.051	0.409	0.059	0.057	0.571	0.058	0.119	0.956
PC aa C36:4	-	PC 36:4	LVEF	0.105	0.052	0.137	0.125	0.057	0.108	-0.037	0.121	0.961
PC aa C36:5	-	PC 36:5	LVEF	0.123	0.052	0.079	0.145	0.057	0.054	-0.015	0.122	0.995
PC aa C36:6	-	PC 36:6	LVEF	**0.613**	**0.210**	**0.004**	**0.217**	**0.057**	**0.003**	-0.011	0.123	0.995
			LVEF'	**-0.083**	**0.056**							
			LVEF''	**0.191**	**0.194**							
PC aa C38:0	-	PC 38:0	LVEF	0.013	0.051	0.876	0.032	0.057	0.750	-0.073	0.110	0.956
PC aa C38:3	-	PC 38:3	LVEF	0.046	0.051	0.616	0.034	0.056	0.748	0.100	0.123	0.956
PC aa C38:4	-	PC 38:4	LVEF	0.052	0.052	0.564	0.061	0.057	0.569	-0.021	0.119	0.990
PC aa C38:5	-	PC 38:5	LVEF	0.098	0.052	0.182	0.112	0.058	0.156	-0.018	0.119	0.990
PC aa C38:6	-	PC 38:6	LVEF	**0.155**	**0.050**	**0.017**	**0.203**	**0.055**	**0.003**	-0.147	0.118	0.946
PC aa C40:1	-	PC 40:1	LVEF	-0.025	0.051	0.791	-0.015	0.057	0.872	-0.064	0.117	0.956
PC aa C40:2	-	PC 40:2	LVEF	-0.136	0.051	0.043	-0.139	0.057	0.069	-0.163	0.110	0.946
PC aa C40:3	-	PC 40:3	LVEF	-0.062	0.052	0.461	-0.051	0.058	0.645	-0.147	0.111	0.946
PC aa C40:4	-	PC 40:4	LVEF	-0.012	0.052	0.886	-0.021	0.057	0.830	0.017	0.119	0.990
PC aa C40:5	-	PC 40:5	LVEF	0.057	0.052	0.499	0.048	0.058	0.651	0.075	0.118	0.956
PC aa C40:6	-	PC 40:6	LVEF	0.102	0.051	0.144	0.129	0.057	0.092	-0.056	0.113	0.956
PC aa C42:0	-	PC 42:0	LVEF	-0.034	0.051	0.722	-0.042	0.058	0.693	0.023	0.119	0.990
PC aa C42:1	-	PC 42:1	LVEF	-0.036	0.052	0.716	-0.033	0.057	0.750	-0.043	0.120	0.961
PC aa C42:2	-	PC 42:2	LVEF	-0.021	0.051	0.815	-0.001	0.056	0.984	-0.113	0.116	0.946
PC aa C42:4	-	PC 42:4	LVEF	-0.134	0.052	0.053	**-0.150**	**0.058**	**0.047**	-0.089	0.118	0.956
PC aa C42:5	-	PC 42:5	LVEF	-0.109	0.052	0.128	-0.113	0.058	0.153	-0.115	0.119	0.946
PC aa C42:6	-	PC 42:6	LVEF	-0.021	0.052	0.822	-0.020	0.058	0.836	-0.053	0.118	0.961
PC ae C30:0	-	PC O-30:0	LVEF	-0.021	0.050	0.815	-0.051	0.055	0.638	0.125	0.119	0.946
PC ae C30:2	-	PC O-30:2	LVEF	0.026	0.048	0.773	0.006	0.054	0.938	0.152	0.113	0.946
PC ae C32:1	-	PC O-32:1	LVEF	-0.129	0.051	0.056	**-0.163**	**0.056**	**0.024**	0.031	0.121	0.983
PC ae C32:2	-	PC O-32:2	LVEF	-0.069	0.048	0.361	-0.093	0.055	0.230	0.041	0.113	0.961
PC ae C34:0	-	PC O-34:0	LVEF	-0.003	0.049	0.966	-0.026	0.054	0.793	0.111	0.115	0.946
PC ae C34:1	-	PC O-34:1	LVEF	-0.054	0.049	0.495	-0.088	0.054	0.249	0.095	0.117	0.956
PC ae C34:2	-	PC O-34:2	LVEF	0.014	0.049	0.876	0.013	0.054	0.876	0.040	0.121	0.961
PC ae C34:3	-	PC O-34:3	LVEF	-0.030	0.050	0.747	-0.022	0.054	0.830	-0.045	0.121	0.961
PC ae C36:0	-	PC O-36:0	LVEF	**-0.191**	**0.049**	**0.003**	**-0.208**	**0.054**	**0.003**	-0.094	0.117	0.956
PC ae C36:1	-	PC O-36:1	LVEF	0.002	0.049	0.966	-0.024	0.054	0.819	0.126	0.115	0.946
PC ae C36:2	-	PC O-36:2	LVEF	0.026	0.048	0.773	0.006	0.053	0.938	0.123	0.118	0.946
PC ae C36:3	-	PC O-36:3	LVEF	0.015	0.050	0.872	0.010	0.056	0.902	0.038	0.119	0.961
PC ae C36:4	-	PC O-36:4	LVEF	0.015	0.053	0.876	0.006	0.059	0.938	0.065	0.119	0.956
PC ae C36:5	-	PC O-36:5	LVEF	-0.030	0.052	0.756	-0.023	0.058	0.830	-0.055	0.115	0.956
PC ae C38:0	-	PC O-38:0	LVEF	**0.155**	**0.051**	**0.019**	**0.190**	**0.057**	**0.008**	-0.047	0.123	0.961
PC ae C38:1	-	PC O-38:1	LVEF	-0.110	0.052	0.126	**-0.151**	**0.057**	**0.044**	0.036	0.122	0.961
PC ae C38:2	-	PC O-38:2	LVEF	0.017	0.051	0.849	0.018	0.056	0.850	0.007	0.116	0.995
PC ae C38:3	-	PC O-38:3	LVEF	0.038	0.052	0.704	0.012	0.058	0.890	0.140	0.119	0.946
PC ae C38:4	-	PC O-38:4	LVEF	0.011	0.052	0.886	-0.021	0.058	0.830	0.148	0.117	0.946
PC ae C38:5	-	PC O-38:5	LVEF	-0.041	0.052	0.668	-0.065	0.059	0.540	0.065	0.113	0.956
PC ae C38:6	-	PC O-38:6	LVEF	0.043	0.052	0.653	0.064	0.058	0.540	-0.056	0.115	0.956
PC ae C40:1	-	PC O-40:1	LVEF	**0.139**	**0.052**	**0.042**	**0.165**	**0.057**	**0.024**	-0.021	0.119	0.990
PC ae C40:2	-	PC O-40:2	LVEF	0.049	0.049	0.565	0.037	0.055	0.718	0.090	0.112	0.956
PC ae C40:3	-	PC O-40:3	LVEF	-0.021	0.051	0.815	-0.045	0.057	0.669	0.091	0.118	0.956
PC ae C40:4	-	PC O-40:4	LVEF	-0.026	0.052	0.790	-0.071	0.057	0.444	0.154	0.116	0.946
PC ae C40:5	-	PC O-40:5	LVEF	-0.030	0.051	0.747	-0.060	0.057	0.570	0.097	0.115	0.956
PC ae C40:6	-	PC O-40:6	LVEF	0.063	0.050	0.423	0.062	0.056	0.537	0.075	0.113	0.956
PC ae C42:0	-	PC O-42:0	LVEF	-0.051	0.052	0.565	-0.037	0.057	0.731	0.875	0.453	0.938
			LVEF'							-0.287	0.147	
			LVEF''							0.679	0.419	
PC ae C42:1		PC O-42:1	LVEF	-0.023	0.052	0.815	-0.026	0.058	0.819	-0.020	0.120	0.990
PC ae C42:2	-	PC O-42:2	LVEF	0.027	0.051	0.781	0.041	0.057	0.693	-0.051	0.120	0.961
PC ae C42:3	-	PC O-42:3	LVEF	0.014	0.051	0.876	0.033	0.056	0.750	-0.075	0.123	0.956

PC ae C42:4	-	PC O-42:4	LVEF	-0.058	0.052	0.479	-0.093	0.057	0.247	0.103	0.120	0.956
PC ae C42:5	-	PC O-42:5	LVEF	-0.071	0.052	0.393	-0.112	0.058	0.156	0.118	0.119	0.946
PC ae C44:3	-	PC O-44:3	LVEF	0.014	0.053	0.876	0.017	0.059	0.864	0.006	0.123	0.995
PC ae C44:4	-	PC O-44:4	LVEF	-0.041	0.051	0.667	-0.053	0.057	0.638	0.012	0.120	0.995
PC ae C44:5	-	PC O-44:5	LVEF	-0.079	0.053	0.332	-0.111	0.058	0.161	0.060	0.122	0.956
PC ae C44:6	-	PC O-44:6	LVEF	-0.070	0.052	0.394	-0.097	0.058	0.235	0.077	0.121	0.956
**Lysophosphatidylcholines**												
lysoPC a C14:0	-	LPC 14:0	LVEF	**0.192**	**0.052**	**0.003**	**0.210**	**0.057**	**0.003**	0.136	0.122	0.946
lysoPC a C16:0	-	LPC 16:0	LVEF	**0.161**	**0.052**	**0.016**	**0.169**	**0.058**	**0.023**	0.135	0.121	0.946
lysoPC a C16:1	-	LPC 16:1	LVEF	0.116	0.051	0.103	0.120	0.057	0.120	0.094	0.120	0.956
lysoPC a C17:0	-	LPC 17:0	LVEF	**0.137**	**0.051**	**0.042**	0.123	0.057	0.112	0.238	0.119	0.938
lysoPC a C18:0	-	LPC 18:0	LVEF	**0.127**	**0.052**	**0.062**	0.131	0.057	0.090	0.158	0.122	0.946
lysoPC a C18:1	-	LPC 18:1	LVEF	0.107	0.051	0.128	0.094	0.057	0.247	0.180	0.120	0.946
lysoPC a C18:2	-	LPC 18:2	LVEF	**0.192**	**0.051**	**0.003**	**0.194**	**0.057**	**0.007**	0.197	0.119	0.946
lysoPC a C20:3	-	LPC 20:3	LVEF	**0.208**	**0.050**	**0.002**	**0.206**	**0.056**	**0.003**	0.203	0.117	0.938
lysoPC a C20:4	-	LPC 20:4	LVEF	**0.180**	**0.052**	**0.006**	**0.190**	**0.058**	**0.009**	0.133	0.122	0.946
lysoPC a C24:0	-	LPC 24:0	LVEF	0.004	0.053	0.962	0.011	0.059	0.902	-0.012	0.123	0.995
lysoPC a C26:0	-	LPC 26:0	LVEF	0.061	0.053	0.477	0.076	0.059	0.407	-0.025	0.123	0.990
lysoPC a C26:1	-	LPC 26:1	LVEF	0.038	0.053	0.708	0.050	0.059	0.651	-0.018	0.120	0.990
lysoPC a C28:0	-	PC O-28:0	LVEF	0.033	0.053	0.744	0.040	0.059	0.718	0.005	0.122	0.995
lysoPC a C28:1	-	PC O-28:1	LVEF	0.087	0.051	0.234	0.076	0.057	0.391	0.171	0.117	0.946
**Sphingolipids**												
SM C16:0	-	SM 34:1	LVEF	-0.058	0.051	0.479	-0.088	0.057	0.283	0.117	0.112	0.946
SM C16:1	-	SM 34:2	LVEF	-0.034	0.050	0.722	-0.039	0.058	0.718	0.026	0.108	0.989
SM C18:0	-	SM 36:1	LVEF	0.047	0.051	0.598	0.039	0.058	0.718	0.087	0.112	0.956
SM C18:1	-	SM 36:2	LVEF	0.043	0.049	0.619	0.044	0.058	0.669	0.067	0.110	0.956
SM C20:2	-	SM 38:3	LVEF	0.056	0.050	0.487	0.063	0.056	0.537	0.007	0.121	0.995
SM C22:3	-	SM 40:4	LVEF	0.087	0.052	0.247	0.104	0.057	0.195	-0.041	0.122	0.961
SM C24:0	-	SM 42:1	LVEF	0.105	0.052	0.138	0.121	0.058	0.125	0.001	0.120	0.996
SM C24:1	-	SM 42:2	LVEF	-0.031	0.053	0.747	-0.025	0.059	0.830	-0.079	0.118	0.956
SM C26:0	-	SM 44:1	LVEF	0.070	0.052	0.394	0.047	0.058	0.669	0.204	0.118	0.938
SM C26:1	-	SM 44:2	LVEF	0.029	0.052	0.773	0.021	0.058	0.830	0.073	0.118	0.956
SM (OH) C14:1	-	SM 33:1	LVEF	0.012	0.048	0.876	-0.020	0.054	0.830	0.201	0.109	0.938
SM (OH) C16:1	-	SM 35:1	LVEF	0.017	0.048	0.849	-0.019	0.055	0.830	0.214	0.106	0.938
SM (OH) C22:1	-	SM 41:1	LVEF	0.114	0.051	0.107	0.128	0.058	0.106	0.035	0.121	0.961
SM (OH) C22:2	-	SM 41:2	LVEF	0.014	0.049	0.876	0.020	0.057	0.830	-0.001	0.119	0.996
SM (OH) C24:1	-	SM 43:1	LVEF	0.072	0.052	0.392	0.088	0.058	0.302	-0.014	0.122	0.995
**Sugars**												
H1	Hexoses (including glucose)	-	LVEF	-0.034	0.050	0.722	-0.054	0.057	0.638	0.121	0.107	0.946

Supplemental Table S7. Associations of left ventricular end diastolic diameter (LVEDD$^{acc. to HENRY}$) and the metabolites.

Results from regression models adjusted for sex, age and BMI for the whole study population and from sex-specific models adjusted for age and BMI.

Metabolites were log2-transformed. Estimates are presented for z-transformed outcome and exposure variables.

* Biocrates updated the nomenclature for fatty acid and lipid classes in 2023 to the standard nomenclature used by LIPID MAPS

stderr, standard error; FDR, false discovery rate

				All subjects			Men			Women		
**Outcome**	**Biochemical**	**New nomenclatur***	**Exposure**	**beta**	**stderr**	**FDR**	**beta**	**stderr**	**FDR**	**beta**	**stderr**	**FDR**
**Acylcarnitines**												
C0	Carnitine	-	LVEDD acc. to HENRY	0.064	0.050	0.532	0.050	0.057	0.989	0.124	0.120	0.968
C10	Decanoylcarnitine	-	LVEDD acc. to HENRY	0.060	0.052	0.650	0.038	0.058	0.989	0.151	0.119	0.968
C10:1	Decenoylcarnitine	-	LVEDD acc. to HENRY	0.053	0.052	0.753	0.072	0.058	0.907	-0.019	0.121	0.968
C10:2	Decadienylcarnitine	-	LVEDD acc. to HENRY	0.027	0.052	0.944	0.044	0.059	0.989	-0.041	0.121	0.968
C12	Dodecanoylcarnitine	-	LVEDD acc. to HENRY	0.023	0.053	0.944	-0.019	0.058	0.989	0.204	0.119	0.968
C12-DC	Dodecanedioylcarnitine	-	LVEDD acc. to HENRY	-0.020	0.052	0.952	-0.020	0.058	0.989	-0.018	0.121	0.968
C14	Tetradecanoylcarnitine	-	LVEDD acc. to HENRY	-0.016	0.053	0.982	-0.044	0.059	0.989	0.108	0.121	0.968
C14:2	Tetradecadienylcarnitine	-	LVEDD acc. to HENRY	0.020	0.053	0.952	0.023	0.059	0.989	0.006	0.121	0.980
C16	Hexadecanoylcarnitine	-	LVEDD acc. to HENRY	-0.038	0.053	0.944	-0.070	0.059	0.907	0.105	0.121	0.968
C16:1	Hexadecenoylcarnitine	-	LVEDD acc. to HENRY	0.012	0.053	0.982	0.008	0.059	0.995	0.028	0.121	0.968
C16:2	Hexadecadienylcarnitine	-	LVEDD acc. to HENRY	0.002	0.053	0.988	0.000	0.059	0.997	0.012	0.121	0.968
C18	Octadecanoylcarnitine	-	LVEDD acc. to HENRY	0.004	0.053	0.988	-0.022	0.059	0.989	0.114	0.120	0.968
C18:1	Octadecenoylcarnitine	-	LVEDD acc. to HENRY	0.012	0.052	0.982	0.004	0.059	0.997	0.052	0.121	0.968
C2	Acetylcarnitine	-	LVEDD acc. to HENRY	0.067	0.052	0.528	0.013	0.057	0.989	0.270	0.117	0.968
C3	Propionylcarnitine	-	LVEDD acc. to HENRY	0.050	0.048	0.741	0.072	0.056	0.907	-0.018	0.120	0.968
C4	Butyrylcarnitine	-	LVEDD acc. to HENRY	0.047	0.052	0.846	0.029	0.058	0.989	0.120	0.120	0.968
C5	Valerylcarnitine	-	LVEDD acc. to HENRY	0.006	0.051	0.988	-0.012	0.059	0.989	0.074	0.119	0.968
C6 / C4:1-DC	Hexanoylcarnitine (Fumarylcarnitine)	-	LVEDD acc. to HENRY	-0.025	0.053	0.944	-0.035	0.059	0.989	0.018	0.121	0.968
**Amino acids**												
Ala	Alanine	-	LVEDD acc. to HENRY	0.024	0.050	0.944	0.041	0.058	0.989	-0.043	0.121	0.968
Arg	Arginine	-	LVEDD acc. to HENRY	0.000	0.052	0.997	0.007	0.059	0.995	-0.032	0.121	0.968
Asn	Asparagine	-	LVEDD acc. to HENRY	0.010	0.052	0.982	0.019	0.059	0.989	-0.026	0.121	0.968
Asp	Aspartate	-	LVEDD acc. to HENRY	-0.113	0.053	0.214	-0.143	0.058	0.554	0.048	0.120	0.968
Cit	Citrulline	-	LVEDD acc. to HENRY	0.010	0.052	0.988	0.000	0.058	0.997	0.052	0.121	0.968
Gln	Glutamine	-	LVEDD acc. to HENRY	0.007	0.052	0.988	0.034	0.058	0.989	-0.111	0.119	0.968
Glu	Glutamate	-	LVEDD acc. to HENRY	-0.029	0.051	0.944	-0.038	0.058	0.989	0.006	0.120	0.980
Gly	Glycine	-	LVEDD acc. to HENRY	-0.030	0.052	0.944	-0.045	0.058	0.989	0.029	0.121	0.968
His	Histidine	-	LVEDD acc. to HENRY	-0.717	0.251	0.222	-0.649	0.273	0.710	-0.118	0.119	0.968
			LVEDD acc. to HENRY'	0.207	0.084		0.196	0.090				
			LVEDD acc. to HENRY''	-0.508	0.230		-0.514	0.259				
Ile	Isoleucine	-	LVEDD acc. to HENRY	0.011	0.050	0.982	-0.012	0.058	0.989	0.111	0.120	0.968
Leu	Leucine	-	LVEDD acc. to HENRY	-0.025	0.049	0.944	-0.055	0.058	0.989	0.108	0.120	0.968
Lys	Lysine	-	LVEDD acc. to HENRY	-0.604	0.249	0.214	-0.753	0.271	0.554	-0.066	0.121	0.968
			LVEDD acc. to HENRY'	0.152	0.083		0.216	0.089				
			LVEDD acc. to HENRY''	-0.315	0.227		-0.520	0.258				
Met	Methionine	-	LVEDD acc. to HENRY	0.009	0.050	0.988	0.019	0.059	0.989	-0.039	0.121	0.968
Orn	Ornithine	-	LVEDD acc. to HENRY	0.071	0.051	0.484	0.086	0.058	0.846	0.025	0.121	0.968
Phe	Phenylalanine	-	LVEDD acc. to HENRY	0.002	0.051	0.988	0.014	0.059	0.989	-0.053	0.121	0.968
Pro	Proline	-	LVEDD acc. to HENRY	-0.025	0.051	0.944	-0.017	0.059	0.989	-0.067	0.121	0.968
Ser	Serine	-	LVEDD acc. to HENRY	0.069	0.053	0.528	0.044	0.059	0.989	0.184	0.119	0.968
Thr	Threonine	-	LVEDD acc. to HENRY	-0.672	0.254	0.214	-0.657	0.275	0.615	0.057	0.120	0.968
			LVEDD acc. to HENRY'	0.171	0.085		0.172	0.091				
			LVEDD acc. to HENRY''	-0.380	0.232		-0.424	0.261				
Trp	Tryptophan	-	LVEDD acc. to HENRY	-0.029	0.050	0.944	-0.032	0.059	0.989	-0.025	0.121	0.968
Tyr	Tyrosine	-	LVEDD acc. to HENRY	0.029	0.049	0.944	0.034	0.058	0.989	0.017	0.121	0.968
Val	Valine	-	LVEDD acc. to HENRY	-0.639	0.242	0.214	-0.669	0.271	0.554	0.079	0.119	0.968
			LVEDD acc. to HENRY'	0.179	0.081		0.176	0.089				
			LVEDD acc. to HENRY''	-0.417	0.221		-0.421	0.257				
**Biogenic amines**		-										
ADMA	Asymmetric dimethylarginine	-	LVEDD acc. to HENRY	-0.039	0.052	0.944	-0.052	0.058	0.989	0.018	0.121	0.968
Ac-Orn	Acetylornithine	-	LVEDD acc. to HENRY	-0.022	0.054	0.945	0.009	0.061	0.995	-0.139	0.122	0.968
Creatinine	Creatinine	-	LVEDD acc. to HENRY	-0.085	0.049	0.323	-0.107	0.058	0.590	-0.013	0.120	0.968
DOPA	Dopamine	-	LVEDD acc. to HENRY	0.148	0.094	0.382	0.150	0.107	0.875			
Histamine	Histamine	-	LVEDD acc. to HENRY	-0.039	0.056	0.944	-0.065	0.061	0.989	0.078	0.131	0.968
Kynurenine	Kynurenine	-	LVEDD acc. to HENRY	-0.007	0.050	0.988	0.001	0.058	0.997	-0.040	0.120	0.968
Met-SO	Methionine sulfoxide	-	LVEDD acc. to HENRY	-0.029	0.053	0.944	-0.038	0.059	0.989	0.013	0.121	0.968
Putrescine	Putrescine	-	LVEDD acc. to HENRY	-0.081	0.287	0.323	0.096	0.309	0.554			
			LVEDD acc. to HENRY'	0.096	0.097		0.056	0.103				
			LVEDD acc. to HENRY''	-0.359	0.267		-0.298	0.300				
SDMA	Symmetric dimethylarginine	-	LVEDD acc. to HENRY	-0.051	0.053	0.806	-0.075	0.059	0.907	0.097	0.121	0.968
Serotonin	Serotonin	-	LVEDD acc. to HENRY	-0.037	0.055	0.944	-0.076	0.061	0.907	0.120	0.123	0.968
Spermidine	Spermidine	-	LVEDD acc. to HENRY	-0.019	0.052	0.954	-0.002	0.059	0.997	-0.106	0.119	0.968
Spermine	Spermine	-	LVEDD acc. to HENRY	-0.071	0.053	0.513	-0.081	0.059	0.875	-0.021	0.121	0.968
Taurine	Taurine	-	LVEDD acc. to HENRY	-0.006	0.053	0.988	-0.025	0.059	0.989	0.073	0.121	0.968
alpha-AAA	Alpha-Aminoadipic acid	-	LVEDD acc. to HENRY	0.031	0.053	0.944	0.001	0.060	0.997	0.172	0.120	0.968
t4-OH-Pro	Trans-4-Hydroxyproline	-	LVEDD acc. to HENRY	0.088	0.052	0.323	0.073	0.058	0.907	0.165	0.118	0.968
total DMA	Total Dimethylarginine	-	LVEDD acc. to HENRY	-0.024	0.051	0.944	-0.043	0.057	0.989	0.067	0.121	0.968
Arg/ADMA	Arginine/Asymmetric dimethylarginine	-	LVEDD acc. to HENRY	0.032	0.053	0.944	0.047	0.058	0.989	-0.041	0.120	0.968
Arg/SDMA	Arginine/Symmetric dimethylarginine	-	LVEDD acc. to HENRY	0.045	0.053	0.874	0.069	0.059	0.929	-0.104	0.121	0.968
Kynurenine/Trp	Kynurenine/Tryptophan	-	LVEDD acc. to HENRY	0.013	0.052	0.982	0.022	0.058	0.989	-0.024	0.121	0.968
**Phosphatidylcholines**												
PC aa C24:0	-	PC 24:0	LVEDD acc. to HENRY	0.048	0.053	0.846	0.043	0.059	0.989	0.069	0.121	0.968
PC aa C26:0	-	PC 26:0	LVEDD acc. to HENRY	0.034	0.053	0.944	0.040	0.059	0.989	0.709	0.558	0.968
			LVEDD acc. to HENRY'							-0.342	0.175	
			LVEDD acc. to HENRY''							1.111	0.504	
PC aa C28:1	-	PC 28:1	LVEDD acc. to HENRY	-0.036	0.051	0.944	-0.038	0.058	0.989	-0.029	0.121	0.968
PC aa C30:0	-	PC 30:0	LVEDD acc. to HENRY	-0.041	0.052	0.921	-0.036	0.058	0.989	-0.066	0.121	0.968
PC aa C30:2	-	PC 30:2	LVEDD acc. to HENRY	0.045	0.052	0.872	0.044	0.058	0.989	0.048	0.119	0.968
PC aa C32:0	-	PC 32:0	LVEDD acc. to HENRY	-0.034	0.052	0.944	-0.060	0.058	0.989	0.074	0.120	0.968
PC aa C32:1	-	PC 32:1	LVEDD acc. to HENRY	-0.130	0.051	0.214	-0.124	0.056	0.554	-0.156	0.120	0.968
PC aa C32:2	-	PC 32:2	LVEDD acc. to HENRY	-0.059	0.051	0.650	-0.034	0.057	0.989	-0.171	0.119	0.968
PC aa C32:3	-	PC 32:3	LVEDD acc. to HENRY	-0.432	0.234	0.214	-0.527	0.266	0.554	0.097	0.121	0.968

			LVEDD acc. to HENRY'	0.069	0.078		0.093	0.088				
			LVEDD acc. to HENRY''	-0.082	0.214		-0.149	0.253				
PC aa C34:1	-	PC 34:1	LVEDD acc. to HENRY	-0.097	0.051	0.267	-0.100	0.057	0.613	-0.081	0.121	0.968
PC aa C34:2	-	PC 34:2	LVEDD acc. to HENRY	-0.624	0.249	0.214	-0.669	0.268	0.554	-0.028	0.121	0.968
			LVEDD acc. to HENRY'	0.142	0.083		0.159	0.088				
			LVEDD acc. to HENRY''	-0.312	0.228		-0.382	0.254				
PC aa C34:3	-	PC 34:3	LVEDD acc. to HENRY	-0.076	0.051	0.414	-0.056	0.056	0.989	-0.167	0.120	0.968
PC aa C34:4	-	PC 34:4	LVEDD acc. to HENRY	-0.022	0.052	0.944	-0.004	0.058	0.997	-0.105	0.120	0.968
PC aa C36:0	-	PC 36:0	LVEDD acc. to HENRY	-0.405	0.254	0.214	0.061	0.058	0.989	0.139	0.119	0.968
			LVEDD acc. to HENRY'	0.109	0.085							
			LVEDD acc. to HENRY''	-0.214	0.232							
PC aa C36:1	-	PC 36:1	LVEDD acc. to HENRY	-0.042	0.052	0.886	-0.022	0.057	0.989	-0.152	0.119	0.968
PC aa C36:2	-	PC 36:2	LVEDD acc. to HENRY	-0.675	0.250	0.222	0.003	0.057	0.997	-0.058	0.121	0.968
			LVEDD acc. to HENRY'	0.191	0.084							
			LVEDD acc. to HENRY''	-0.456	0.229							
PC aa C36:3	-	PC 36:3	LVEDD acc. to HENRY	-0.080	0.051	0.382	-0.074	0.057	0.907	-0.106	0.120	0.968
PC aa C36:4	-	PC 36:4	LVEDD acc. to HENRY	-0.029	0.052	0.944	-0.059	0.058	0.989	0.102	0.119	0.968
PC aa C36:5	-	PC 36:5	LVEDD acc. to HENRY	-0.004	0.052	0.988	0.000	0.058	0.997	-0.020	0.121	0.968
PC aa C36:6	-	PC 36:6	LVEDD acc. to HENRY	-0.147	0.252	0.337	-0.004	0.058	0.997	-0.108	0.121	0.968
			LVEDD acc. to HENRY'	-0.024	0.084							
			LVEDD acc. to HENRY''	0.147	0.230							
PC aa C38:0	-	PC 38:0	LVEDD acc. to HENRY	-0.448	0.253	0.238	0.045	0.059	0.989	0.076	0.120	0.968
			LVEDD acc. to HENRY'	0.117	0.085							
			LVEDD acc. to HENRY''	-0.241	0.231							
PC aa C38:3	-	PC 38:3	LVEDD acc. to HENRY	-0.076	0.052	0.426	-0.051	0.058	0.989	-0.803	0.549	0.968
			LVEDD acc. to HENRY'							0.053	0.173	
			LVEDD acc. to HENRY''							0.055	0.496	
PC aa C38:4	-	PC 38:4	LVEDD acc. to HENRY	0.004	0.052	0.988	-0.015	0.058	0.989	0.002	0.548	0.968
			LVEDD acc. to HENRY'							-0.116	0.172	
			LVEDD acc. to HENRY''							0.534	0.495	
PC aa C38:5	-	PC 38:5	LVEDD acc. to HENRY	-0.008	0.052	0.988	-0.022	0.058	0.989	0.061	0.120	0.968
PC aa C38:6	-	PC 38:6	LVEDD acc. to HENRY	-0.555	0.253	0.214	-0.603	0.272	0.554	0.077	0.121	0.968
			LVEDD acc. to HENRY'	0.106	0.085		0.118	0.090				
			LVEDD acc. to HENRY''	-0.201	0.231		-0.252	0.259				
PC aa C40:1	-	PC 40:1	LVEDD acc. to HENRY	0.032	0.052	0.944	0.045	0.058	0.989	-0.028	0.118	0.968
PC aa C40:2	-	PC 40:2	LVEDD acc. to HENRY	-0.410	0.253	0.214	-0.308	0.273	0.590	0.117	0.120	0.968
			LVEDD acc. to HENRY'	0.091	0.085		0.048	0.090				
			LVEDD acc. to HENRY''	-0.154	0.231		-0.035	0.259				
PC aa C40:3	-	PC 40:3	LVEDD acc. to HENRY	0.014	0.052	0.982	0.007	0.058	0.995	0.049	0.119	0.968
PC aa C40:4	-	PC 40:4	LVEDD acc. to HENRY	-0.011	0.052	0.982	-0.011	0.058	0.995	-0.802	0.560	0.968
			LVEDD acc. to HENRY'							0.127	0.176	
			LVEDD acc. to HENRY''							-0.169	0.507	
PC aa C40:5	-	PC 40:5	LVEDD acc. to HENRY	-0.054	0.052	0.741	-0.044	0.058	0.989	-0.109	0.118	0.968
PC aa C40:6	-	PC 40:6	LVEDD acc. to HENRY	-0.596	0.255	0.222	-0.083	0.058	0.875	-0.035	0.121	0.968
			LVEDD acc. to HENRY'	0.135	0.085							
			LVEDD acc. to HENRY''	-0.299	0.232							
PC aa C42:0	-	PC 42:0	LVEDD acc. to HENRY	-0.023	0.052	0.944	-0.022	0.058	0.989	-0.025	0.121	0.968
PC aa C42:1	-	PC 42:1	LVEDD acc. to HENRY	-0.008	0.053	0.988	-0.008	0.059	0.995	-0.005	0.120	0.981
PC aa C42:2	-	PC 42:2	LVEDD acc. to HENRY	0.014	0.052	0.982	0.012	0.058	0.989	0.023	0.119	0.968
PC aa C42:4	-	PC 42:4	LVEDD acc. to HENRY	-0.574	0.253	0.214	0.017	0.058	0.989	-0.012	0.121	0.968
			LVEDD acc. to HENRY'	0.136	0.085							
			LVEDD acc. to HENRY''	-0.276	0.231							
PC aa C42:5	-	PC 42:5	LVEDD acc. to HENRY	-0.004	0.053	0.988	0.000	0.059	0.997	-0.020	0.119	0.968
PC aa C42:6	-	PC 42:6	LVEDD acc. to HENRY	0.026	0.053	0.944	0.031	0.058	0.989	0.002	0.121	0.987
PC ae C30:0	-	PC O-30:0	LVEDD acc. to HENRY	0.002	0.053	0.988	0.019	0.058	0.989	-0.071	0.121	0.968
PC ae C30:2	-	PC O-30:2	LVEDD acc. to HENRY	-0.011	0.052	0.982	-0.022	0.059	0.989	0.040	0.121	0.968
PC ae C32:1	-	PC O-32:1	LVEDD acc. to HENRY	-0.014	0.052	0.982	-0.015	0.058	0.989	-0.010	0.121	0.971
PC ae C32:2	-	PC O-32:2	LVEDD acc. to HENRY	0.022	0.051	0.944	0.007	0.058	0.995	0.088	0.120	0.968
PC ae C34:0	-	PC O-34:0	LVEDD acc. to HENRY	-0.004	0.053	0.988	-0.013	0.058	0.989	0.035	0.120	0.968
PC ae C34:1	-	PC O-34:1	LVEDD acc. to HENRY	-0.023	0.052	0.944	-0.014	0.058	0.989	-0.065	0.120	0.968
PC ae C34:2	-	PC O-34:2	LVEDD acc. to HENRY	-0.368	0.248	0.222	0.063	0.058	0.972	0.009	0.121	0.974
			LVEDD acc. to HENRY'	0.085	0.083							
			LVEDD acc. to HENRY''	-0.146	0.227							
PC ae C34:3	-	PC O-34:3	LVEDD acc. to HENRY	0.033	0.052	0.944	0.037	0.057	0.989	0.017	0.121	0.968
PC ae C36:0	-	PC O-36:0	LVEDD acc. to HENRY	0.069	0.052	0.513	0.036	0.057	0.989	0.230	0.118	0.968
PC ae C36:1	-	PC O-36:1	LVEDD acc. to HENRY	-0.480	0.251	0.214	0.012	0.058	0.989	-0.401	0.553	0.968
			LVEDD acc. to HENRY'	0.090	0.084					-0.049	0.174	
			LVEDD acc. to HENRY''	-0.143	0.229					0.359	0.500	
PC ae C36:2	-	PC O-36:2	LVEDD acc. to HENRY	-0.583	0.248	0.214	-0.553	0.270	0.554	-0.022	0.121	0.968
			LVEDD acc. to HENRY'	0.143	0.083		0.147	0.089				
			LVEDD acc. to HENRY''	-0.290	0.227		-0.332	0.257				
PC ae C36:3	-	PC O-36:3	LVEDD acc. to HENRY	-0.457	0.250	0.222	0.069	0.058	0.907	-0.029	0.121	0.968
			LVEDD acc. to HENRY'	0.118	0.084							
			LVEDD acc. to HENRY''	-0.241	0.228							
PC ae C36:4	-	PC O-36:4	LVEDD acc. to HENRY	0.081	0.052	0.387	0.071	0.058	0.907	0.122	0.118	0.968
PC ae C36:5	-	PC O-36:5	LVEDD acc. to HENRY	-0.198	0.253	0.218	0.064	0.058	0.972	0.185	0.115	0.968
			LVEDD acc. to HENRY'	0.036	0.085							
			LVEDD acc. to HENRY''	-0.014	0.231							
PC ae C38:0	-	PC O-38:0	LVEDD acc. to HENRY	-0.465	0.249	0.214	-0.520	0.270	0.554	0.012	0.121	0.968
			LVEDD acc. to HENRY'	0.082	0.084		0.104	0.089				
			LVEDD acc. to HENRY''	-0.121	0.228		-0.198	0.256				
PC ae C38:1	-	PC O-38:1	LVEDD acc. to HENRY	0.097	0.053	0.283	0.051	0.059	0.989	0.274	0.117	0.968
PC ae C38:2	-	PC O-38:2	LVEDD acc. to HENRY	-0.477	0.252	0.238	0.009	0.058	0.995	0.056	0.121	0.968
			LVEDD acc. to HENRY'	0.110	0.084							
			LVEDD acc. to HENRY''	-0.214	0.230							
PC ae C38:3	-	PC O-38:3	LVEDD acc. to HENRY	-0.518	0.252	0.222	-0.001	0.058	0.997	-0.594	0.558	0.968
			LVEDD acc. to HENRY'	0.112	0.084					0.015	0.175	
			LVEDD acc. to HENRY''	-0.216	0.230					0.174	0.505	
PC ae C38:4	-	PC O-38:4	LVEDD acc. to HENRY	0.083	0.052	0.379	0.080	0.058	0.880	-0.074	0.548	0.968
			LVEDD acc. to HENRY'							-0.096	0.173	
			LVEDD acc. to HENRY''							0.479	0.496	

PC ae C38:5	-	PC O-38:5	LVEDD acc. to HENRY	-0.468	0.252	0.214	0.082	0.058	0.875	0.161	0.116	0.968
			LVEDD acc. to HENRY'	0.141	0.084							
			LVEDD acc. to HENRY''	-0.303	0.230							
PC ae C38:6	-	PC O-38:6	LVEDD acc. to HENRY	-0.338	0.252	0.214	-0.332	0.274	0.554	0.114	0.120	0.968
			LVEDD acc. to HENRY'	0.067	0.084		0.066	0.090				
			LVEDD acc. to HENRY''	-0.082	0.231		-0.089	0.260				
PC ae C40:1	-	PC O-40:1	LVEDD acc. to HENRY	-0.410	0.254	0.323	-0.025	0.058	0.989	0.106	0.120	0.968
			LVEDD acc. to HENRY'	0.080	0.085							
			LVEDD acc. to HENRY''	-0.136	0.232							
PC ae C40:2	-	PC O-40:2	LVEDD acc. to HENRY	0.004	0.052	0.988	-0.015	0.059	0.989	0.086	0.121	0.968
PC ae C40:3	-	PC O-40:3	LVEDD acc. to HENRY	-0.510	0.251	0.222	0.015	0.058	0.989	0.045	0.121	0.968
			LVEDD acc. to HENRY'	0.125	0.084							
			LVEDD acc. to HENRY''	-0.255	0.229							
PC ae C40:4	-	PC O-40:4	LVEDD acc. to HENRY	0.046	0.052	0.872	0.042	0.058	0.989	0.060	0.120	0.968
PC ae C40:5	-	PC O-40:5	LVEDD acc. to HENRY	-0.525	0.252	0.214	-0.459	0.274	0.606	0.144	0.118	0.968
			LVEDD acc. to HENRY'	0.137	0.084		0.119	0.090				
			LVEDD acc. to HENRY''	-0.277	0.230		-0.254	0.260				
PC ae C40:6	-	PC O-40:6	LVEDD acc. to HENRY	-0.511	0.253	0.214	0.028	0.059	0.989	-0.712	0.559	0.968
			LVEDD acc. to HENRY'	0.125	0.085					0.111	0.176	
			LVEDD acc. to HENRY''	-0.249	0.231					-0.109	0.506	
PC ae C42:0	-	PC O-42:0	LVEDD acc. to HENRY	-0.712	0.252	0.214	-0.775	0.271	0.554	0.118	0.119	0.968
			LVEDD acc. to HENRY'	0.229	0.085		0.238	0.089				
			LVEDD acc. to HENRY''	-0.568	0.231		-0.619	0.258				
PC ae C42:1	-	PC O-42:1	LVEDD acc. to HENRY	-0.649	0.252	0.244	-0.644	0.270	0.684	0.015	0.121	0.968
			LVEDD acc. to HENRY'	0.196	0.084		0.201	0.089				
			LVEDD acc. to HENRY''	-0.477	0.230		-0.530	0.257				
PC ae C42:2	-	PC O-42:2	LVEDD acc. to HENRY	0.011	0.052	0.982	0.007	0.058	0.995	0.029	0.119	0.968
PC ae C42:3	-	PC O-42:3	LVEDD acc. to HENRY	-0.453	0.252	0.323	-0.444	0.270	0.590	0.074	0.121	0.968
			LVEDD acc. to HENRY'	0.101	0.084		0.078	0.089				
			LVEDD acc. to HENRY''	-0.195	0.230		-0.121	0.257				
PC ae C42:4	-	PC O-42:4	LVEDD acc. to HENRY	0.000	0.052	0.997	0.009	0.058	0.995	-0.041	0.121	0.968
PC ae C42:5	-	PC O-42:5	LVEDD acc. to HENRY	0.023	0.052	0.944	0.013	0.058	0.989	0.060	0.120	0.968
PC ae C44:3	-	PC O-44:3	LVEDD acc. to HENRY	-0.585	0.254	0.214	-0.566	0.274	0.554	-0.052	0.121	0.968
			LVEDD acc. to HENRY'	0.149	0.085		0.141	0.090				
			LVEDD acc. to HENRY''	-0.321	0.232		-0.305	0.260				
PC ae C44:4	-	PC O-44:4	LVEDD acc. to HENRY	-0.021	0.052	0.945	-0.006	0.058	0.997	-0.093	0.120	0.968
PC ae C44:5	-	PC O-44:5	LVEDD acc. to HENRY	0.003	0.053	0.988	0.021	0.058	0.989	-0.071	0.120	0.968
PC ae C44:6	-	PC O-44:6	LVEDD acc. to HENRY	0.002	0.053	0.988	0.026	0.058	0.989	-0.106	0.120	0.968
**Lysophosphatidylcholines**												
lysoPC a C14:0	-	LPC 14:0	LVEDD acc. to HENRY	-0.011	0.053	0.982	0.013	0.058	0.989	-0.125	0.120	0.968
lysoPC a C16:0	-	LPC 16:0	LVEDD acc. to HENRY	-0.067	0.052	0.528	-0.069	0.058	0.907	-0.061	0.121	0.968
lysoPC a C16:1	-	LPC 16:1	LVEDD acc. to HENRY	-0.105	0.052	0.222	-0.093	0.057	0.684	-0.165	0.120	0.968
lysoPC a C17:0	-	LPC 17:0	LVEDD acc. to HENRY	-0.309	0.256	0.330	0.018	0.059	0.989	-0.053	0.121	0.968
			LVEDD acc. to HENRY'	0.044	0.086							
			LVEDD acc. to HENRY''	-0.032	0.233							
lysoPC a C18:0	-	LPC 18:0	LVEDD acc. to HENRY	0.003	0.052	0.988	0.014	0.058	0.989	-0.048	0.121	0.968
lysoPC a C18:1	-	LPC 18:1	LVEDD acc. to HENRY	-0.038	0.052	0.944	-0.028	0.058	0.989	-0.087	0.121	0.968
lysoPC a C18:2	-	LPC 18:2	LVEDD acc. to HENRY	-0.034	0.052	0.944	-0.016	0.058	0.989	-0.110	0.120	0.968
lysoPC a C20:3	-	LPC 20:3	LVEDD acc. to HENRY	-0.084	0.052	0.350	-0.052	0.058	0.989	-0.225	0.116	0.968
lysoPC a C20:4	-	LPC 20:4	LVEDD acc. to HENRY	-0.009	0.053	0.988	-0.003	0.058	0.997	-0.035	0.121	0.968
lysoPC a C24:0	-	LPC 24:0	LVEDD acc. to HENRY	0.045	0.053	0.872	0.055	0.059	0.989	-0.004	0.121	0.981
lysoPC a C26:0	-	LPC 26:0	LVEDD acc. to HENRY	0.120	0.254	0.214	0.013	0.274	0.615	0.011	0.121	0.968
			LVEDD acc. to HENRY'	-0.113	0.085		-0.073	0.090				
			LVEDD acc. to HENRY''	0.397	0.232		0.305	0.260				
lysoPC a C26:1	-	LPC 26:1	LVEDD acc. to HENRY	0.029	0.052	0.944	-0.015	0.058	0.989	0.214	0.118	0.968
lysoPC a C28:0	-	PC O-28:0	LVEDD acc. to HENRY	0.034	0.053	0.944	0.047	0.059	0.989	-0.033	0.121	0.968
lysoPC a C28:1	-	PC O-28:1	LVEDD acc. to HENRY	0.024	0.052	0.944	0.026	0.059	0.989	0.016	0.121	0.968
**Sphingolipids**												
SM C16:0	-	SM 34:1	LVEDD acc. to HENRY	0.021	0.052	0.945	0.020	0.058	0.989	0.024	0.121	0.968
SM C16:1	-	SM 34:2	LVEDD acc. to HENRY	-0.486	0.243	0.214	-0.553	0.271	0.554	0.023	0.121	0.968
			LVEDD acc. to HENRY'	0.094	0.081		0.119	0.089				
			LVEDD acc. to HENRY''	-0.155	0.222		-0.238	0.257				
SM C18:0	-	SM 36:1	LVEDD acc. to HENRY	-0.037	0.051	0.944	-0.063	0.058	0.972	0.088	0.121	0.968
SM C18:1	-	SM 36:2	LVEDD acc. to HENRY	-0.610	0.240	0.214	-0.682	0.271	0.554	0.117	0.120	0.968
			LVEDD acc. to HENRY'	0.154	0.080		0.174	0.089				
			LVEDD acc. to HENRY''	-0.335	0.219		-0.417	0.258				
SM C20:2	-	SM 38:3	LVEDD acc. to HENRY	-0.055	0.050	0.686	-0.038	0.056	0.989	-0.132	0.120	0.968
SM C22:3	-	SM 40:4	LVEDD acc. to HENRY	-0.018	0.052	0.964	-0.037	0.058	0.989	0.062	0.120	0.968
SM C24:0	-	SM 42:1	LVEDD acc. to HENRY	-0.013	0.052	0.982	0.015	0.058	0.989	-0.154	0.120	0.968
SM C24:1	-	SM 42:2	LVEDD acc. to HENRY	-0.581	0.253	0.297	-0.016	0.058	0.989	0.033	0.121	0.968
			LVEDD acc. to HENRY'	0.152	0.085							
			LVEDD acc. to HENRY''	-0.344	0.231							
SM C26:0	-	SM 44:1	LVEDD acc. to HENRY	0.032	0.052	0.944	0.021	0.058	0.989	0.088	0.120	0.968
SM C26:1	-	SM 44:2	LVEDD acc. to HENRY	-0.010	0.053	0.982	-0.024	0.059	0.989	0.059	0.121	0.968
SM (OH) C14:1	-	SM 33:1	LVEDD acc. to HENRY	0.015	0.052	0.982	0.027	0.058	0.989	-0.041	0.121	0.968
SM (OH) C16:1	-	SM 35:1	LVEDD acc. to HENRY	0.029	0.052	0.944	0.027	0.058	0.989	0.043	0.121	0.968
SM (OH) C22:1	-	SM 41:1	LVEDD acc. to HENRY	-0.265	0.250	0.323	0.022	0.058	0.989	-0.075	0.121	0.968
			LVEDD acc. to HENRY'	0.028	0.084							
			LVEDD acc. to HENRY''	0.010	0.228							
SM (OH) C22:2	-	SM 41:2	LVEDD acc. to HENRY	-0.422	0.243	0.214	-0.309	0.274	0.554	-0.038	0.121	0.968
			LVEDD acc. to HENRY'	0.080	0.081		0.044	0.090				
			LVEDD acc. to HENRY''	-0.117	0.222		-0.021	0.260				
SM (OH) C24:1	-	SM 43:1	LVEDD acc. to HENRY	0.023	0.053	0.944	0.018	0.059	0.989	0.050	0.121	0.968
**Sugars**												
H1	Hexoses (including glucose)		LVEDD acc. to HENRY	0.013	0.052	0.982	0.021	0.059	0.989	-0.034	0.121	0.968
